# Construction of congested Csp^3^–Csp^3^ bonds by a formal Ni-catalyzed alkylboration†

**DOI:** 10.1039/d1sc00900a

**Published:** 2021-03-09

**Authors:** Amit Kumar Simlandy, Stephen R. Sardini, M. Kevin Brown

**Affiliations:** Department of Chemistry, Indiana University 800E. Kirkwood Ave Bloomington IN 47401 USA brownmkb@indiana.edu

## Abstract

Through the combination of a Ni-catalyzed alkene alkenylboration followed by hydrogenation, the synthesis of congested Csp^3^–Csp^3^-bonds can be achieved. Conditions have been identified that allow for the use of both alkenyl-bromides and -triflates. In addition, the hydrogenation creates another opportunity for stereocontrol, thus allowing access to multiple stereoisomers of the product. Finally, the method is demonstrated in the streamlined synthesis of a biologically relevant molecule.

## Abstract 2

Through the combination of a Ni-catalyzed alkene alkenylboration followed by hydrogenation, the synthesis of congested Csp^3^–Csp^3^-bonds can be achieved.

## Graphical abstract

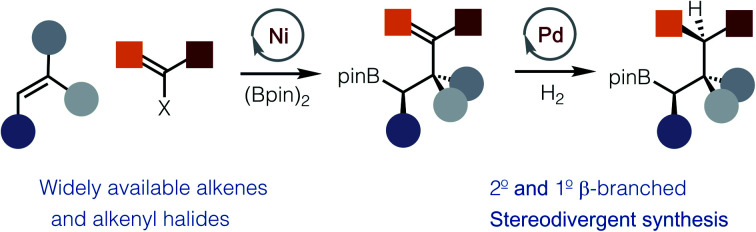

## Introduction

**Scheme 1:**
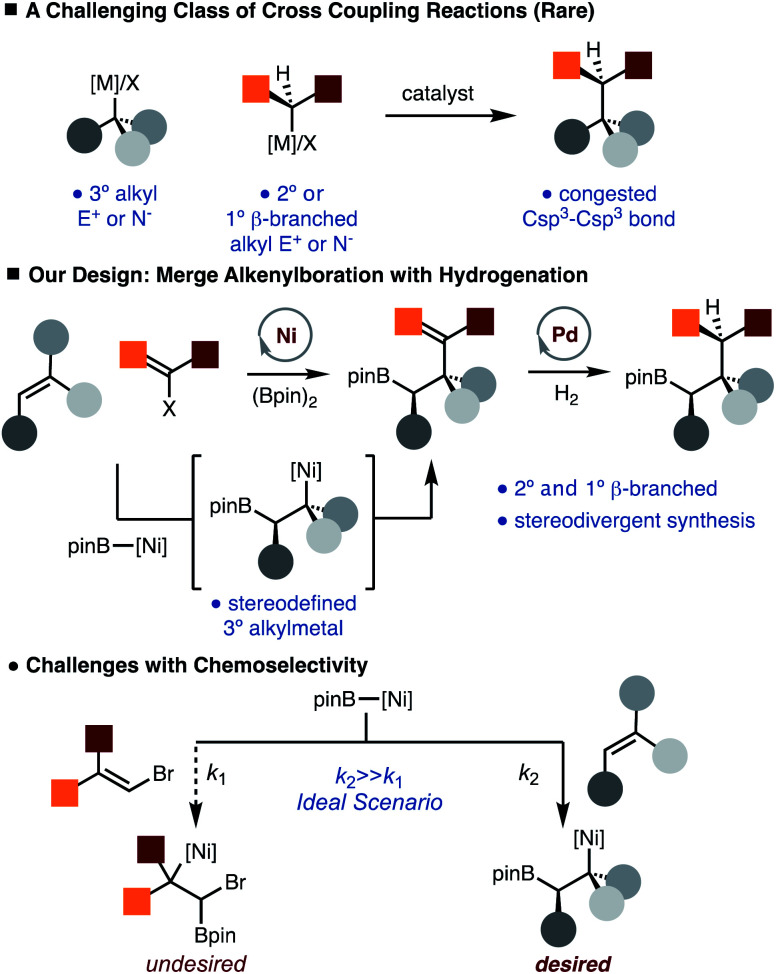
Congested bond synthesis by cross coupling.

Recent studies have demonstrated that molecules with an increased proportion of Csp^3^ centers can often result in improved pharmacological properties (*i.e.* “escape from flatland”).^1^ Therefore, development of methods that facilitate the synthesis of Csp^3^–Csp^3^ bonds is of value. While much progress has been made in the development of alkyl–alkyl cross-coupling reactions, synthesis of congested Csp^3^–Csp^3^ bonds remains a formidable challenge (Scheme 1).^2^ In particular, generation of products that would arise from the direct coupling of 2° and 3° alkyl fragments is not known. In addition, use of 1° β-branched alkyl electrophiles is also known to be challenging.^3^ Therefore, introduction of protocols that would achieve the synthesis of these congested bonds would provide an important tool in the construction of complex Csp^3^ rich molecules.

Our lab has recently disclosed the Ni-catalyzed arylboration of unactivated alkenes.^4,5^ The value of these methods lies in that simple starting materials (alkenes, diboron reagents, and carbon-based electrophiles) are converted to more complex structures with control of diastereoselectivity and regioselectivity in one step, and the reactivity of the resulting C–B bond, which can be easily transformed into C–O, C–N and C–C bonds thus allowing for diverse product formation.^6^ One of the key aspects of the Ni-catalyzed arylboration reaction is that sterically demanding di- and tri-substituted unactivated alkenes can be used. In these reactions, a stereodefined tertiary alkyl-Ni-complex is generated by borylnickelation of an alkene, which undergoes facile reaction with an arylbromide.^5^ To address the aforementioned challenge of making Csp^3^–Csp^3^ bonds, we envisioned a net alkylboration that would merge an alkenylboration and subsequent hydrogenation (Scheme 1).^7–9^ This strategy is appealing as the hydrogenation event offers an additional point of stereocontrol such that diverse products can be generated from a common set of starting materials. While coupling of 3° alkyl and aryl fragments is known,^10^ the use of alkenyl partners that would allow for synthesis of congested Csp^3^–Csp^3^ bonds by subsequent hydrogenation is exceedingly rare.^11,12^ The outlined approach does bring to light a chemoselectivity challenge in that conditions must be tuned to favor borylnickelation of the alkene rather than alkenyl bromide (Scheme 1).

Under conditions optimized for arylboration of alkenes,^5*c*^ it was identified that alkenyl halides could be used to deliver the desired alkenylboration products. Furthermore, hydrogenation with Pd/C proceeded in high yield to generate the products of a net alkylboration. The use of 1-bromo-2-methylpropene allowed for the introduction of an isobutyl group with a variety of alkenes (products **1–14**). Both sterically demanding trisubstituted and 1,1-disubstituted alkenes function well in the process to generate a congested C–C bond between a quaternary carbon and a β-branched primary carbon. A focus of these efforts was on the synthesis of saturated nitrogen containing heterocycles, which are of value in medicinal chemistry.^13^ Particularly notable are examples **12** and **13** as these are generated as single observable diastereomers. In addition, reaction to produce **6**, occurred with high selectivity for addition on the face opposite the ester. Reactions of more strained alkenes generally result in higher yield (compare product **11** with **13**/**14**). Finally, with 1-bromo-2-methylpropene the reaction of cyclic 1,2-disubstiuted alkenes were also investigated, which allowed for the synthesis of *syn*-1,2-substituted 5-membered ring carbo- and heterocycles.

With respect to the alkenyl bromide component, the use of all substitution patterns worked. In the case of **15–19** the coupling proceeded to prepare the sterically congested bond of coupling between 2° and 3° fragments. In the case of products **23** and **26**, the hydrogenation reaction led to formation of diastereomers with variable selectivity. The formal introduction of a 1° alkyl group can also be achieved with use of bromides **20a**/**b** and **21**. With the former example, both *E* and *Z*-alkenyl bromide work with equal efficiency. In addition, functional groups such as acetals (product **2**) and silyl ethers were tolerated (product **28**). Finally, the reaction can be performed on gram scale as demonstrated in the synthesis of **4**.

**Scheme 2:**
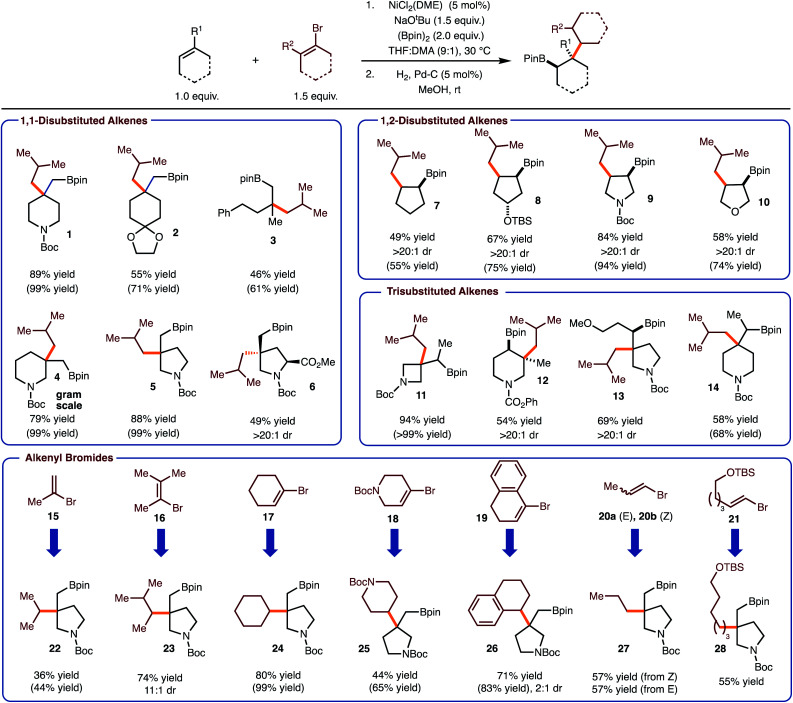
Ni-catalyzed formal alkylboration: scope. Reactions run on 0.3 mmol. Yield of isolated product after silica gel column chromatography (average of 2 or more runs). Yield in parentheses was determined by ^1^H NMR analysis of the unpurified reaction mixture after the first step. In some cases accurate NMR yield was not possible due to overlapping signals.

With the data gathered in Scheme 2, some general trends are revealed regarding chemoselectivity of [Ni]-Bpin addition. In general, the use of sterically demanding tri- and tetra-substituted alkenyl bromides led to formation of the product in the highest yields. In these cases, it is likely that *k*_1_ is reduced due to steric hindrance, thus allowed *k*_2_ to predominate (Scheme 1). For reactions with disubstituted alkenyl bromides **15**, **20–21**, competitive borylation of the alkene was observed. Despite this challenge, products **22** and **27–28** could still be formed in acceptable yields. However, it is notable that in most cases the addition of [Ni]-Bpin proceeds with high chemoselectivity for addition to the unactivated alkene in preference to the alkenyl bromide.

**Scheme 3:**
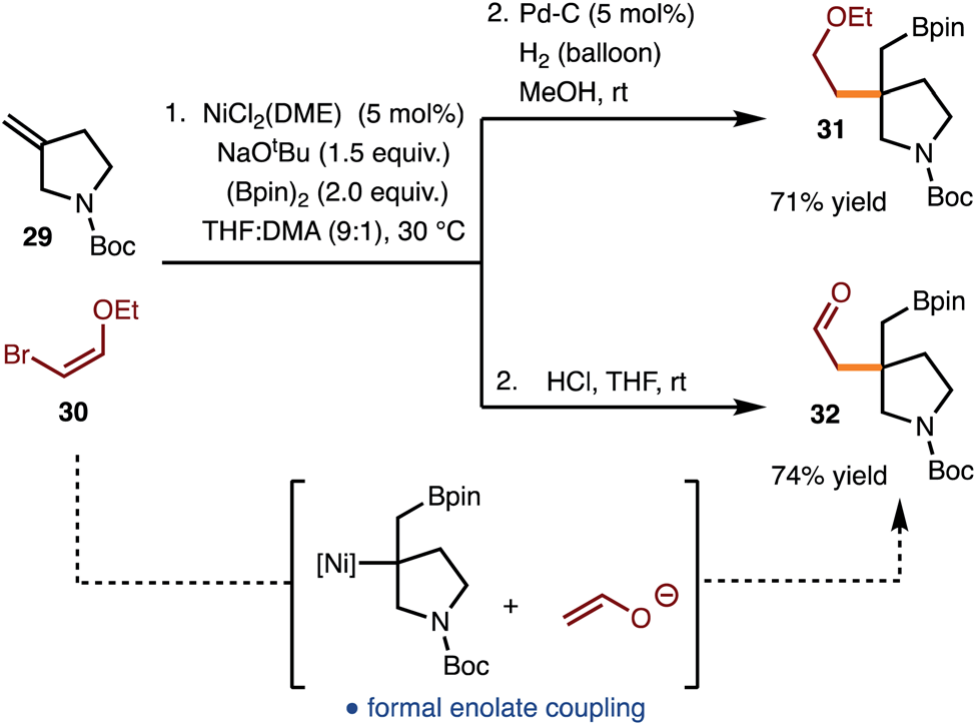
Reaction with bromo enol ether **30**.

The reaction of commercially available bromo enol ether **30** was also investigated (Scheme 3). Standard hydrogenation of the alkenylboration product led to the formation of **31**. Alternatively, hydrolysis of the alkenylboration product allowed for the formation of aldehyde **32**, which represents the formal coupling of an enolate with the generated alkyl Ni-complex.

While the use of alkenyl bromides is convenient due to commercial availability, the use of enol-triflates would also be of value as they are readily prepared from the corresponding aldehydes or ketones. Under the optimized conditions, however, only 60% of **35** was observed (Table 1, entry 1). We hypothesized that bromide ion may be important for reactivity. Thus, sodium bromide was added, however, only a modest increase in yield was observed (Table 1, entry 2). Interestingly, when NiBr_2_(DME) was evaluated, a significant increase in yield relative to use of NiCl_2_(DME) was observed (Table 1, entry 3). Based on the observation that additives impacted the reaction yield, other salts were investigated, which led to the finding that using NaBF_4_ led to the highest yield of product (Table 1, entry 9). At this stage, the role of NaBF_4_ is not clear. It should also be noted that the use of NaBF_4_ was explored in reactions of the alkenyl bromides; however, no beneficial effect was observed.

**Table 1:** Reaction of enol triflates

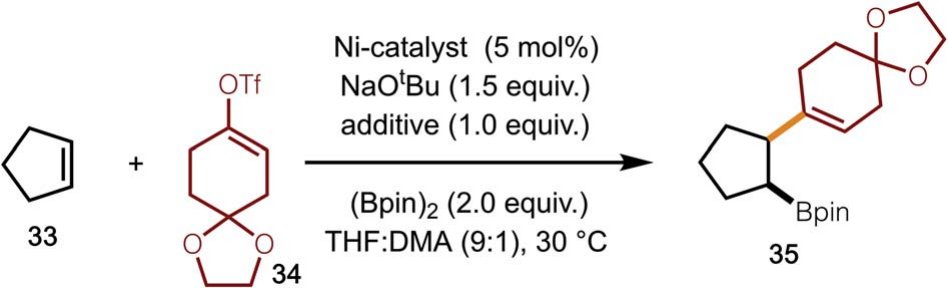			
Entry	Ni-catalyst	Additive	Yield^a^ (%)
1	NiCl_2_(DME)	None	60
2	NiCl_2_(DME)	NaBr^b^	64
3	NiBr_2_(DME)	None	77
4	NiBr_2_(DME)	NaCl^c^	79
5	NiBr_2_(DME)	NaBr^c^	21
6	NiBr_2_(DME)	NaOTf	87
7	NiBr_2_(DME)	NaPF_6_	52
8	NiBr_2_(DME)	NaSbF_6_	19
**9**	***NiBr***_***2***_ ***(DME)***	***NaBF***_***4***_	***91 (70)*** d

a Yield determined by analysis of the unpurified reaction mixture with an internal standard.

b 30 mol % additive.

c 60 mol % additive.

d Yield in parentheses is of isolated product after silica gel column chromatography.

For hydrogenation of **35** with Pd/C, the formation of isomers **36**, **37** and **38** were observed (Table 2, entry 1).^14^ It is likely that *anti*-isomer **37** is formed after hydrogenation of an *in situ* generated tetrasubstituted alkene. Use of Crabtree's catalyst did lead to suppressed formation of *anti*-isomer **37**, however alkene isomers (**38**) were still observed (Table 2, entry 2). Finally, it was discovered that HAT hydrogenation allowed for exclusive formation of **36**, without undesired isomerization (Table 2, entry 3).^15^

**Table 2:** Alkene reduction

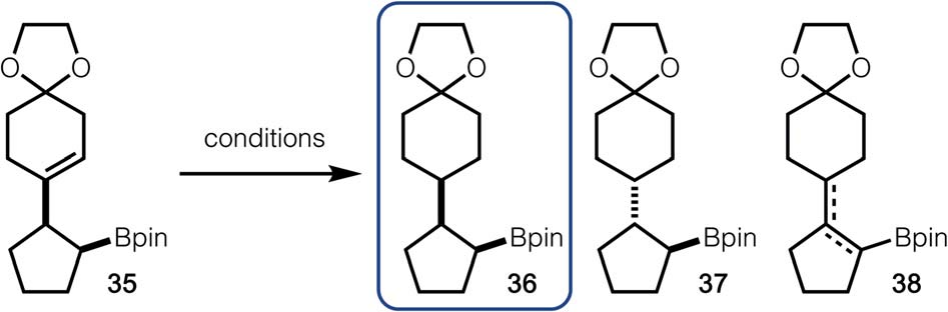		
Entry	Conditions	Yield^a^ (%) (**36** : **37** : **38**)
1	5 mol % Pd–C, H_2_ (balloon), EtOAc, rt	95% yield (1 : 1 : 6 : 0)
2	5 mol % Crabtree's cat., H_2_ (balloon), CH_2_Cl_2_, 0 °C	84% yield (5 : 0 : 1)
3	Mn(dpm)_3_, PhSiH_2_(Oi-Pr), TBHP, hexanes, rt	66% yield^b^ (1 : 0 : 0)

a Yield determined by analysis of the unpurified reaction mixture by ^1^H NMR with an internal standard.

b Yield of isolated product after silica gel column chromatography.

**Scheme 4:**
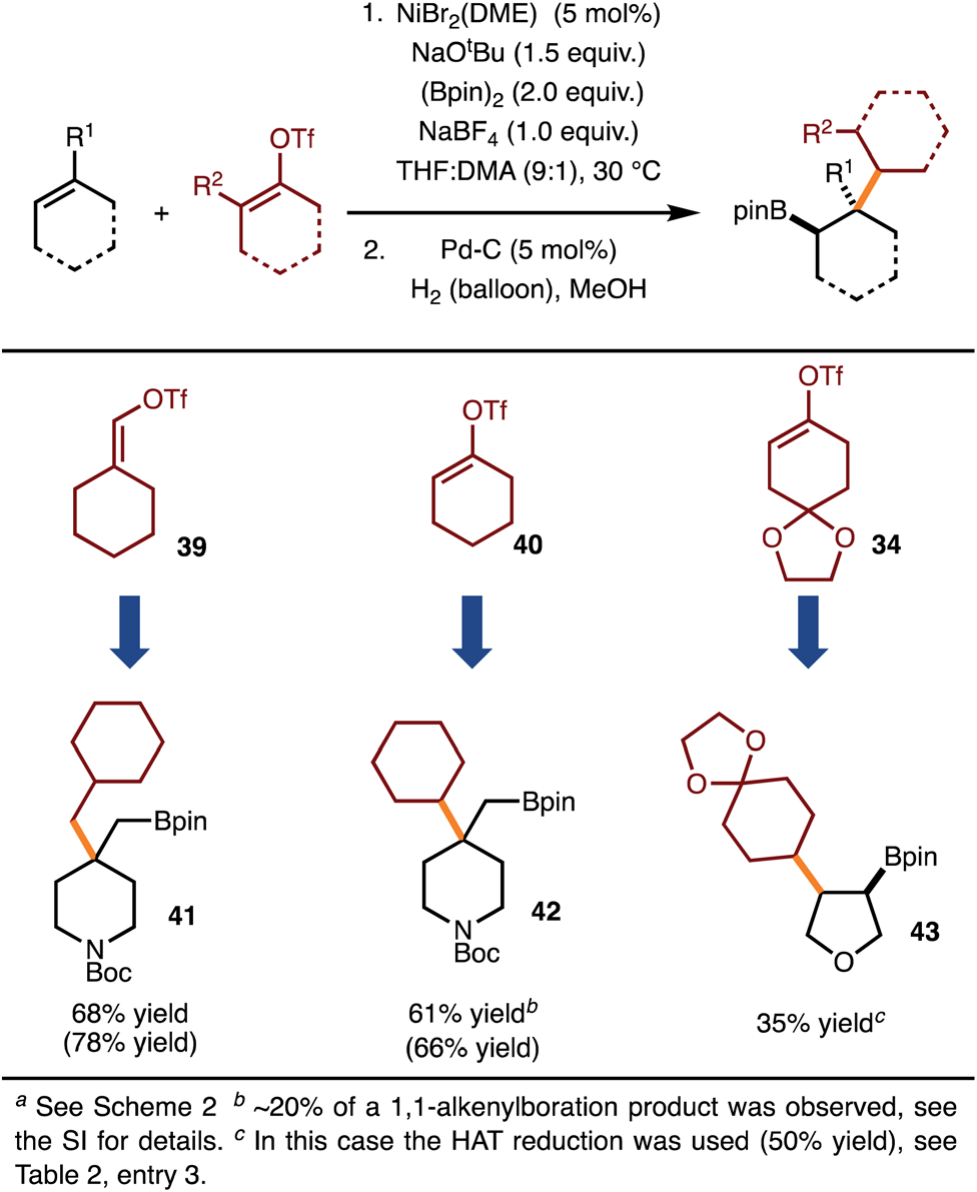
Reaction with alkenyl triflates.

Under the optimal conditions for coupling with alkenyl triflates, several examples were investigated (Scheme 4). The use of cyclohexenyl triflate **39** allowed for the formation of **41**, whereas use of triflate **40** led to synthesis of **42**. For the synthesis of **43**, the moderate yield was the result of a 50% yield in the hydrogenation step.

**Scheme 5:**
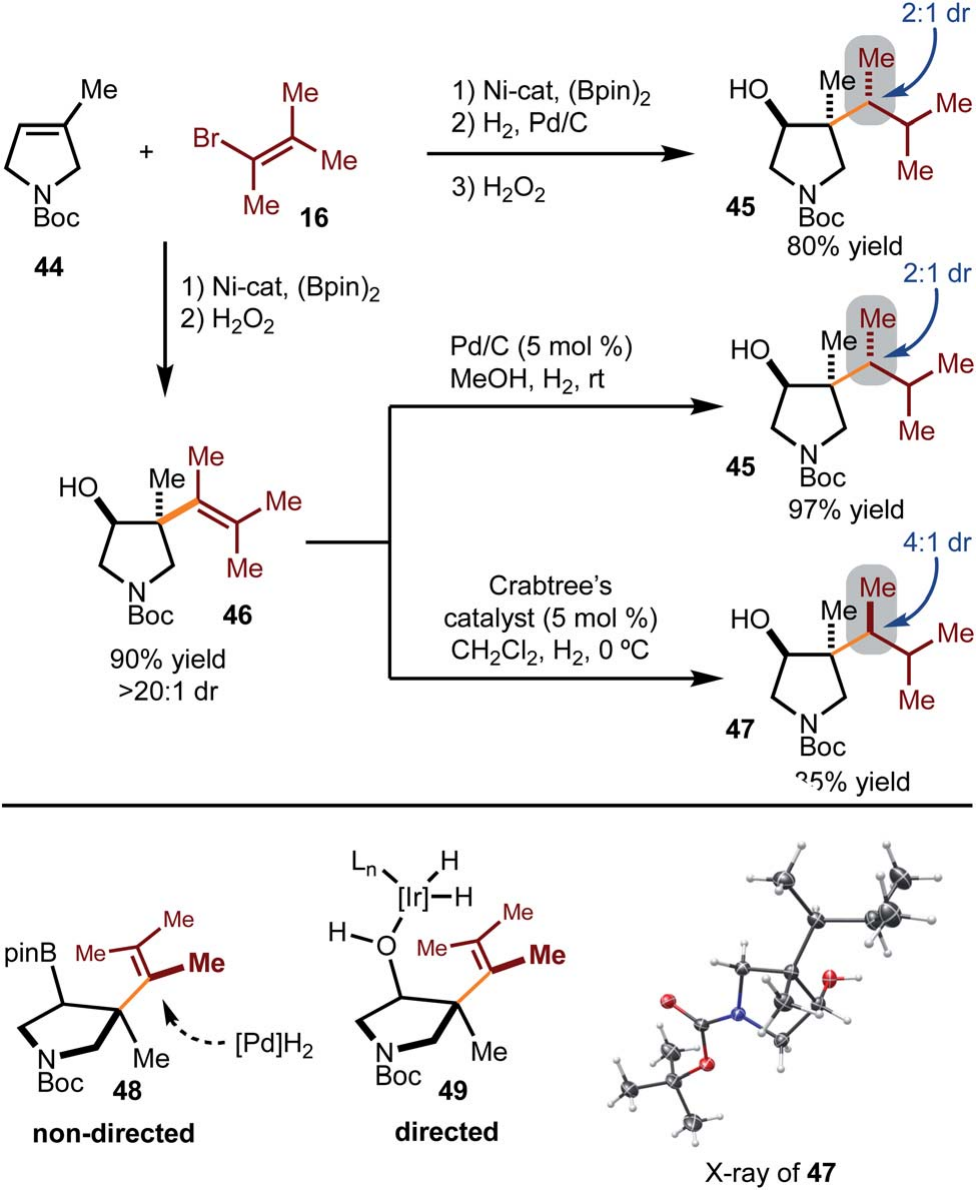
Diastereoselective hydrogenation.

Hydrogenation with substrates that would result in diastereomers was probed more deeply (Scheme 5). Hydrogenation of the alkenylboration product derived from **44** and **16** gave rise to **45** as the major diastereomer in 2 : 1 dr. Other hydrogenation conditions were attempted, but poor reactivity was observed. Hydrogenation of the corresponding alcohol **46** was also probed. Under heterogeneous conditions, the same major diastereomer (**45**) was formed as that observed in the reduction of the Bpin-derived substrate. Based on the stereochemistry of product **45**, reduction likely occurs from the least hindered face, as shown in model **48**. On the other hand, directed hydrogenation of the alcohol with Crabtree's catalyst led to formation of the opposite diastereomer (**47**), likely *via* intermediate **49**.^16^ It is important to note that while the selectivities are modest, this strategy demonstrates that tuning of conditions can allow for stereodivergent synthesis. In addition, these examples demonstrate that three contiguous stereogenic centers can be prepared from simple components in a modular fashion.

**Scheme 6:**
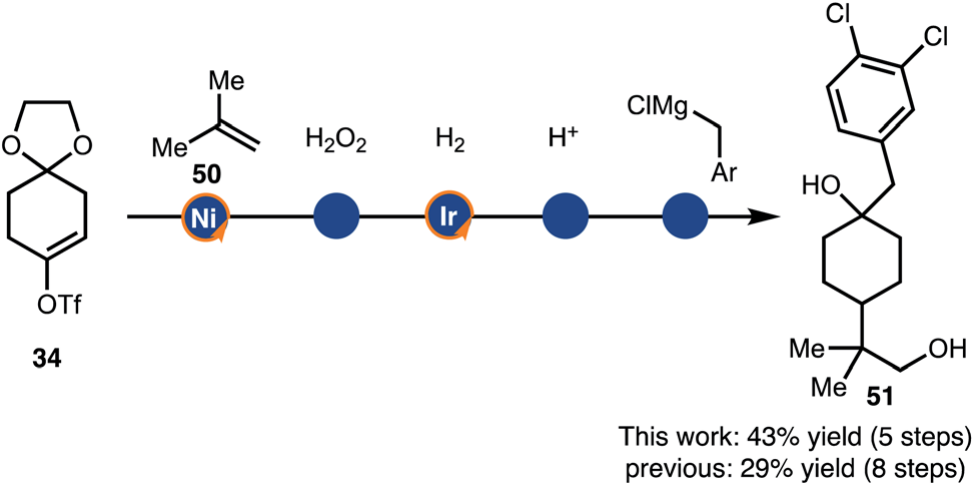
Streamlined synthesis of **51**.

Finally, the alkenylboration/hydrogenation sequence was used in the synthesis of drug like intermediates (Scheme 6). Compound **51** was prepared though a brief sequence of five steps in 43% overall starting from **34** and **50**. Thus, the demonstrated strategy offers an alternative to the established route that required eight steps.^17^

## Conclusions

In summary, the synthesis of sterically congested Csp^3^–Csp^3^ bonds by a formal alkylboration of unactivated alkenes is reported. The process was made possible by combining a Ni-catalyzed alkenylboration followed by hydrogenation. In addition, the hydrogenation could be tuned to achieve stereodivergent synthesis. Through the development of this process, we have demonstrated the utility of Ni-catalyzed carboboration for the generation of molecular complexity with high Csp^3^ content.

## Author contributions

A. K. S., S. R. S and M. K. B. designed the study. A. K. S. and S. R. S. performed the experiements. A. K. S. and M. K. B. wrote the paper with input from S. R. S.

## Conflicts of interest

There are no conflicts to declare.

## Supplementary Material

SC-012-D1SC00900A-s001

SC-012-D1SC00900A-s002
