# Supplementary material for: Construction of congested Csp3–Csp3 bonds by a formal Ni-catalyzed alkylboration
Source: Chem Sci. 2021 Mar 9;12(15):5517–21. doi: 10.1039/d1sc00900a (PMC8179681; doi:10.1039/d1sc00900a)
Supplement: SC-012-D1SC00900A-s001 [file SC-012-D1SC00900A-s001.pdf]

Electronic Supplementary Material (ESI) for Chemical Science.
This journal is © The Royal Society of Chemistry 2021

Construction of Congested Csp^3-Csp^3 Bond by a Formal Ni-Catalyzed Alkylboration

Amit Kumar Simlandy, Stephen R. Sardini, M. Kevin Brown*

Department of Chemistry, Indiana University, 800 E. Kirkwood Ave, Bloomington, IN 47405

1. General Information S2
2. Reagents and Catalysts S3
3. Substrate Synthesis S5
4. General Procedure for Vinyl Bromides S6
5. Characterization Data for Vinyl Bromide Derived Products S7
6. Optimization Table for Vinyl Triflate S23
7. General Procedure for Vinyl Triflates S23
8. Optimization of Reduction Conditions for 1,2 Alkenylboration Product **35** S24
9. Characterization Data for Vinyl Triflate Derived Products S25
10. Synthesis of *Tamoxilog* Analog S29
11. Single Crystal X-ray Diffraction Analysis of **47** S33
12. Spectra S35
13. References S70

1. General information:

Infrared (IR) spectra were recorded on a Bruker Tensor II FT-IR Spectrometer, ν_{max} in cm^{-1}. Bands are characterized as broad (br), strong (s), medium (m), and weak (w). ^1H NMR spectra were recorded at room temperature unless otherwise noted on a Varian I400 (400 MHz), Varian VXR400 (400 MHz), Varian I500 (500 MHz), or a Varian I600 (600 MHz) spectrometer. Chemical shifts are reported in ppm from tetramethylsilane with the residual solvent resonance as the internal standard ($CHCl_3$: δ 7.26 ppm). Data are reported as follows: chemical shift, multiplicity (s = singlet, d = doublet, t = triplet, q = quartet, br = broad, m = multiplet), coupling constants (Hz), and integration. ^{13}C NMR spectra were recorded on a Varian I400 (100 MHz), Varian VXR400 (100 MHz), Varian I500 (125 MHz), or a Varian I600 (150 MHz) spectrometer with complete proton decoupling. Chemical shifts are reported in ppm from tetramethylsilane with the solvent resonance as the internal standard ($CDCl_3$: δ 77.16 ppm). ^{19}F NMR spectra were recorded on Varian VXR400 (375 MHz) spectrometer. High-resolution mass spectrometry (HRMS) was performed on either a Waters/Micromass LCT Classic (ESI-TOF) or a Thermo Electron Corporation MAT 95XP-Trap (GC/MS). The diastereomeric and regioisomeric ratios were determined using NMR analysis of unpurified reaction mixtures. GC analyses were performed by means of Agilent 6850 Gas Chromatograph equipped with Agilent 19091Z-413E, 30 m x 320 μm x 0.25 μm column. Helium was used as the GC carrier gas and maintained at a constant flow rate of 25.0 mL/min. The capillary column was held for 1.0 minutes at the initial temperature (60 °C) and subsequently ramped at a rate of 25 °C /min to a final temperature of 300 °C. Total run time was 9.60 min.

Unless otherwise noted, all reactions have been carried out with distilled and degassed solvents under an atmosphere of dry N_2 in oven- (135 °C) and flame-dried glassware with standard vacuum-line techniques. Tetrahydrofuran and *N*,*N*-Dimethylformamide were purified under a positive pressure of dry argon by passage through two columns of activated alumina. Methanol was purchased from Macron Fine Chemicals and used as received. Toluene was purified under a positive pressure of dry argon by passage through columns of activated alumina and Q5 (Grubbs apparatus). All work-up and purification procedures were carried out with reagent grade solvents (purchased from Sigma-Aldrich) in air. Standard column chromatography techniques using ZEOprep 60/40-63 μm silica gel was used for purification.

2. Reagents and Catalysts:

(3-Methoxypropyl)triphenylphosphonium bromide was synthesized in accordance with literature procedure.[1]

***tert*-Butyl 3-oxopyrrolidine-1-carboxylate** was purchased from Combi-Blocks and used as received.

***tert*-Butyl(cyclopent-3-en-1-yloxy)dimethylsilane** was synthesized in accordance with literature procedure.[2]

Cyclopentene was purchased from Alfa Aesar and purified via neat filtration through a 2-cm pad of dry silica in a 5.75-inch pipet prior to use.

2,5-Dihydrofuran was purchased from Sigma-Aldrich and purified via neat filtration through a 2-cm pad of dry silica in a 5.75-inch pipet prior to use.

Phenyl 5-methyl-3,6-dihydropyridine-1(2*H*)-carboxylate was synthesized in accordance with literature procedure.[3]

***tert*-Butyl 3-ethylideneazetidine-1-carboxylate** was synthesized in accordance with literature procedure.[4]

1-(*tert*-Butyl) 2-methyl (*S*)-4-methylenepyrrolidine-1,2-dicarboxylate was synthesized in accordance with literature procedure.[3]

***tert*-Butyl 2,5-dihydro-1*H*-pyrrole-1-carboxylate** was purchased from Combi-Blocks and used as received.

***tert*-Butyl 3-methyl-2,5-dihydro-1*H*-pyrrole-1-carboxylate** was synthesized in accordance with literature procedure.[5]

***tert*-Butyl 3-methylenepiperidine-1-carboxylate** was synthesized in accordance with literature procedure.[6]

***tert*-Butyl 4-ethylidenepiperidine-1-carboxylate** was synthesized in accordance with literature procedure.[7]

***tert*-Butyl 4-methylenepiperidine-1-carboxylate** was synthesized in accordance with literature procedure.[8]

***tert*-Butyl 4-ethylidenepiperidine-1-carboxylate** was synthesized in accordance with literature procedure.[7]

8-Methylene-1,4-dioxaspiro[4.5]decane was synthesized in accordance with literature procedure.[3]

1-Bromo-2-methylprop-1-ene was purchased from Combi-Blocks and purified via neat filtration through a 2-cm pad of dry silica in a 5.75-inch pipet prior to use.

2-Bromo-3-methylbut-2-ene was purchased from Sigma-Aldrich and purified via neat filtration through a 2-cm pad of dry silica in a 5.75-inch pipet prior to use.

2-Bromoprop-1-ene was purchased from Oakwood and purified via neat filtration through a 2-cm pad of dry silica in a 5.75-inch pipet prior to use.

(*Z*)-1-Bromoprop-1-ene was purchased from Sigma-Aldrich and purified via neat filtration through a 2-cm pad of dry silica in a 5.75-inch pipet prior to use.

(*E*)-1-Bromoprop-1-ene was purchased from Sigma-Aldrich and purified via neat filtration through a 2-cm pad of dry silica in a 5.75-inch pipet prior to use.

(*E*)-((6-Bromohex-5-en-1-yl)oxy)(*tert*-butyl)dimethylsilane was synthesized in accordance with literature procedure.[9]

4-Bromo-1,2-dihydronaphthalene was synthesized in accordance with literature procedure.[10]

***tert*-Butyl 4-bromo-3,6-dihydropyridine-1(2*H*)-carboxylate** was synthesized in accordance with literature procedure.[11]

(3-Methylbut-3-en-1-yl)benzene was synthesized in accordance with literature procedure.[12]

***tert*-Butyl 3-methylenepyrrolidine-1-carboxylate** was synthesized in accordance with literature procedure.[13]

Bis(pinacolato)diboron was purchased from Oakwood and purified via recrystallization from pentane prior to use.

Dodecane was purchased from Sigma-Aldrich and used as received.

Di-*tert*-butyl dicarbonate was purchased from Oakwood and used as received.

Methyltriphenylphosphonium bromide was purchased from TCI and used as received.

***n*-Butyl lithium (2.5 M in hexane)** was purchased from Sigma-Aldrich in a Sure-Seal™ bottle and titrated prior to use.

1,3,5-Trimethylbenzene was purchased from Sigma-Aldrich and purified via column chromatography (100% pentane) and stored over activated 4Å molecular sieves.

***N*,*N*-Dimethylacetamide (DMA)** was purchased from Sigma-Aldrich in a Sure-Seal™ bottle and used as received.

Nickel(II) chloride ethylene glycol dimethyl ether complex (Ni(DME)Cl_2) was purchased from Strem and used as received.

Potassium *tert*-butoxide was purchased from Oakwood and used as received.

Sodium *tert*-butoxide was purchased from Strem and used as received.

Cyclohex-1-en-1-yl trifluoromethanesulfonate was synthesized in accordance with literature procedure.[14]

1,4-Dioxaspiro[4.5]dec-7-en-8-yl trifluoromethanesulfonate was synthesized in accordance with literature procedure.[15]

Cyclohexylidenemethyl trifluoromethanesulfonate was synthesized in accordance with literature procedure.[16]

Crabtree's catalyst was purchased from Strem and used as received.

Pd-C was purchased from Strem and used as received.

$Mn(dpm)_3$ was purchased from Combi-Blocks and used as received.

***tert*-Butyl hydroperoxide solution (5.5 M in nonane)** was purchased from Sigma-Aldrich and used as received.

Isopropoxy(phenyl)silane was synthesized in accordance with literature procedure.[17]

3. Substrate Synthesis:

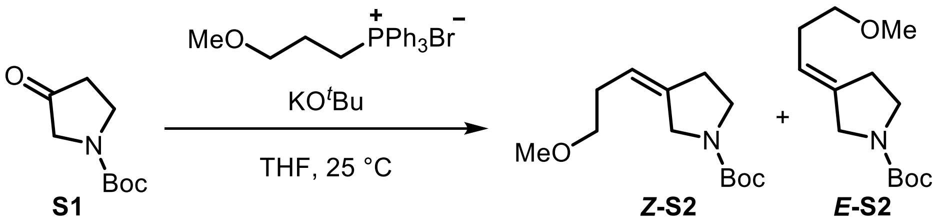

S1 **Z-S2** **E-S2**

***tert*-Butyl (*Z*)-3-(3-methoxypropylidene)pyrrolidine-1-carboxylate (S2):** To a flame-dried round bottom flask was added (3-methoxypropyl)triphenylphosphonium bromide (5.8 g, 14 mmol, 1.4 equiv.) The flask was then inerted with three evacuation/backfill (N_2) cycles before the addition of THF (25 mL) via syringe. The suspension was cooled to 0 ºC and potassium *tert*-butoxide (1.6 g, 14 mmol, 1.4 equiv.) was added. The orange solution was allowed to stir for 30 min. at 0 ºC (ice-bath). The solution of *tert*-butyl 3-oxopyrrolidine-1-carboxylate **S1** (1.85 g, 10.0 mmol, 1.00 equiv.) in THF (5 mL) was then added dropwise via syringe. The reaction was allowed to stir for 18 h at ambient temperature. Pentane (50 mL) was then added, and the suspension was filtered over a glass frit via suction filtration. The filtrate was then concentrated under reduced pressure. Purification via silica gel chromatography (Gradient: 12% to 18% EtOAc:hexane) yields

isomerically pure *Z*-alkene **S2**: yield of (*Z*)-isomer (400 mg, 17% yield). Low yields are due to difficult separation of the alkene isomers.

IR (neat): 2977 (m), 2930 (w), 2879 (w), 1700 (s), 1643 (m), 1479 (m), 1367 (s), 1332 (w), 1245 (w), 1166 (s), 1115 (s), 742 (m) cm^{-1}; **^{1}H NMR (400 MHz, $CDCl_3$, *Z isomer*):** δ 5.49 – 5.11 (m, 1H), 3.91 (s, 2H), 3.49 – 3.36 (m, 4H), 3.34 (s, 3H), 2.53 (t, *J* = 6.9 Hz, 2H), 2.32 – 2.16 (m, 2H), 1.47 (s, 9H); **^{13}C NMR (126 MHz, $CDCl_3$, *Z isomer, mixture of rotamers*):** δ 154.8, 138.8, 137.8, 118.6, 118.4, 79.4, 72.1, 58.8, 47.7, 47.5, 45.6, 45.1, 32.2, 31.5, 30.0, 29.9, 28.7; **HRMS (ESI+):** Calculated for $C_{13}H_{23}O_3NNa$ [M+Na]$^+$: 264.1570, Found: 264.1572.

4. General Procedure for Formal Alkylboration with Vinyl Bromides (General Procedure A):

In an N_2-filled glovebox, to an oven-dried 13 x 100 mm screw-capped vial was added bis(pinacolato)diboron (152 mg, 0.600 mmol, 2.00 equiv.) and sodium *tert*-butoxide (43 mg, 0.45 mmol, 1.5 equiv.). In a separate oven-dried 2-dram vial, Ni(DME)Cl_2 (8.20 mg, 0.0375 mmol) was added. Both vials were sealed with septa and removed from the glovebox. THF (2.7 mL) was added to the reaction vial, followed by alkene (0.3 mmol, 1.0 equiv.) and vinyl bromide (0.45 mmol, 1.5 equiv.). DMA (0.75 mL) was then added to the vial containing Ni(DME)Cl_2 (0.05 M in Ni(DME)Cl_2) to prepare the catalyst solution. The catalyst solution (0.30 mL) was then added to the reaction vial (5 mol% catalyst loading). The septum was then quickly replaced by a Teflon-lined screw cap and the reaction was stirred at 30 °C for 18 h in a preheated metal block. The reaction was quenched upon the addition of 1 M HCl (5.0 mL), and the mixture was extracted with EtOAc (3 x 5.0 mL). The organic layer was washed with 1 M KOH (2 x 5.0 mL), dried over Na_2SO_4, gravity filtered, and concentrated. 1,3,5-Trimethylbenzene was added as an internal standard and a small aliquot was analyzed via ^{1}H-NMR. Purified by silica-gel column chromatography. In an oven dried 10-mL round-bottom flask, alkene was taken in MeOH (3.0 mL) or EtOAc (3.0 mL) under positive pressure of N_2. Pd-C (5 mol%) was added and hydrogen gas was bubbled through the solution for 5 min. The heterogeneous mixture was stirred at room temperature until TLC shows complete consumption of starting alkene. The reaction contents were filtered over celite and washed with EtOAc. The crude reaction mixture was purified by silica-gel column chromatography.

5. Characterization Data for Vinyl Bromide Derived Products:

In ^{13}C NMR spectra, signals of carbons directly bonded to boron were not detected because of quadrupolar relaxation.

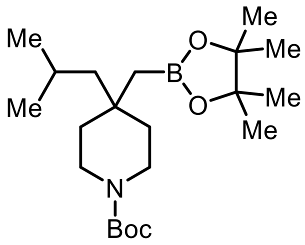

***Tert*-butyl-4-isobutyl-4-((4,4,5,5-tetramethyl-1,3,2-dioxaborolan-2-yl)methyl)piperidine-1-carboxylate (1):** The title compound was prepared according to General Procedure A. NMR yield of the unpurified reaction mixture was determined using 1,3,5-trimethylbenzene as an NMR standard. Hydrogenation was performed using MeOH as solvent. Purification for both the steps by silica gel column chromatography (Gradient: 6% to 9% EtOAc:hexanes) yields **1** as a colorless oil.

Average over 2 runs: 99% NMR yield (alkenylboration step), 89% isolated yield (over 2 steps).

IR (neat): 2976 (w), 2929 (w), 2869 (w), 1692 (s), 1422 (w), 1364 (s), 1320 (m), 1276 (m), 1244 (m), 1144 (s), 1106 (w), 970 (w), 769 (w) cm^{-1}; **^{1}H NMR (400 MHz, CDCl$_3$):** δ 3.47 – 3.41 (m, 2H), 3.25 – 3.18 (m, 2H), 1.71 – 1.64 (m, 1H), 1.41 (s, 13H), 1.26 (d, J = 5.4 Hz, 2H), 1.19 (s, 12H), 0.88 (d, J = 6.5 Hz, 8H); **^{13}C NMR (101 MHz, CDCl$_3$, *mixture of rotamers*):** δ 155.2, 82.9, 79.1, 49.0, 40.5, 39.7, 37.4, 36.8, 34.1, 28.6, 25.6, 25.0, 23.8; **HRMS (APCI+):** Calculated for $C_{21}H_{41}O_4NB$ [M+H]$^+$: 382.3123, Found: 382.3124.

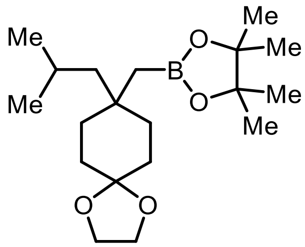

2-((8-Isobutyl-1,4-dioxaspiro[4.5]decan-8-yl)methyl)-4,4,5,5-tetramethyl-1,3,2-dioxaborolane (2): The title compound was prepared according to General Procedure A. The reaction mixture was taken in 20 mL EtOAc and washed with brine. NMR yield of the unpurified reaction mixture was determined using 1,3,5-trimethylbenzene as an NMR standard.

Hydrogenation was performed using EtOAc as solvent. Purification for both the steps by silica gel column chromatography (Gradient: 6% to 8% EtOAc:hexanes) yields **2** as a colorless oil.

Average over 2 runs: 71% NMR yield (alkenylboration step), 55% isolated yield (over 2 steps).

IR (neat): 2976 (w), 2931 (s), 2869 (s), 1468 (w), 1366 (s), 1319 (s), 1273 (w), 1214 (w), 1183 (m), 1144 (s), 1104 (s), 1037 (m), 966 (m), 937 (m), 897 (m), 847 (m) cm^{-1}; **^{1}H NMR (400 MHz, $CDCl_3$):** δ 3.90 (s, 4H), 1.69 – 1.64 (m, 1H), 1.62 – 1.58 (m, 4H), 1.53 – 1.49 (m, 4H), 1.28 (d, *J* = 5.3 Hz, 2H), 1.20 (s, 12H), 0.89 (d, *J* = 6.5 Hz, 8H); **^{13}C NMR (101 MHz, $CDCl_3$):** δ 109.4, 82.8, 64.2, 64.2, 48.6, 35.7, 34.8, 31.0, 25.7, 25.0, 24.0; **HRMS (APCI+):** Calculated for $C_{19}H_{36}O_4B$ $[M+H]^+$: 339.2705, Found: 339.2705.

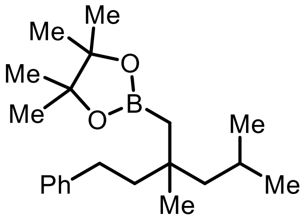

2-(2,4-Dimethyl-2-phenethylpentyl)-4,4,5,5-tetramethyl-1,3,2-dioxaborolane (3): The title compound was prepared according to a modified version of General Procedure A using 1.0 equiv. of vinyl bromide and 1.5 equiv. of alkene. NMR yield of the unpurified reaction mixture was determined using 1,3,5-trimethylbenzene as an NMR standard. Hydrogenation was performed using MeOH as solvent. Purification for both the steps by silica gel column chromatography (Gradient: 1% to 3% Et_2O:hexanes) yields **3** as a colorless oil.

Average over 2 runs: 61% NMR yield (alkenylboration step), 46% isolated yield (over 2 steps).

IR (neat): 3026 (m), 2976 (m), 2929 (m), 1468 (m), 1317 (s), 1142 (s), 969 (m), 697 (s) cm^{-1}; **^{1}H NMR (500 MHz, $CDCl_3$):** δ 7.26 – 7.23 (m, 2H), 7.18 (d, *J* = 7.6 Hz, 2H), 7.14 (t, *J* = 7.3 Hz, 1H), 2.57 – 2.53 (m, 2H), 1.74 – 1.66 (m, 1H), 1.60 – 1.57 (m, 2H), 1.28 (d, *J* = 5.2 Hz, 2H), 1.23 (d, *J* = 1.2 Hz, 12H), 1.01 (s, 3H), 0.92 (d, *J* = 6.6 Hz, 6H), 0.87 (s, 2H); **^{13}C NMR (126 MHz, $CDCl_3$):** δ 144.1, 128.6, 128.4, 125.5, 82.9, 51.0, 45.0, 35.9, 31.0, 27.8, 25.8, 25.7, 25.1, 24.3; **HRMS (EI+):** Calculated for $C_{21}H_{35}BO_2$ $[M]^+$: 329.2766, Found: 329.2765.

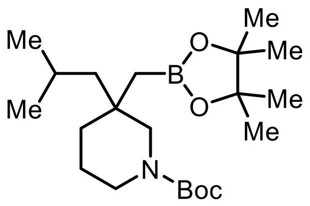

***Tert*-butyl 3-isobutyl-3-((4,4,5,5-tetramethyl-1,3,2-dioxaborolan-2-yl)methyl)piperidine-1-carboxylate (4):** The title compound was prepared according to General Procedure A. NMR yield of the unpurified reaction mixture was determined using 1,3,5-trimethylbenzene as an NMR standard. Hydrogenation was performed using MeOH as solvent. Purification for both the steps by silica gel column chromatography (Gradient: 6% to 8% EtOAc:hexanes) yields **4** as a colorless oil.

Average over 2 runs: 99% NMR yield (alkenylboration step), 79% isolated yield (over 2 steps).

IR (neat): 2976 (w), 2930 (w), 2867 (w), 1692 (s), 1427 (m), 1364 (s), 1321 (m), 1273 (m), 1216 (m), 1160 (s), 1144 (s), 969(w), 848 (w), 766 (w) cm^{-1}; **^{1}H NMR (400 MHz, CDCl$_3$):** δ 3.51 – 3.00 (m, 4H), 1.74 – 1.64 (m, 1H), 1.60 – 1.50 (m, 4H), 1.44 (s, 9H), 1.35-1.30 (m, 2H), 1.23 (s, 12H), 0.92 (d, J = 5.4 Hz, 3H), 0.90 (d, J = 5.4 Hz, 3H), 0.88 – 0.75 (m, 2H); **^{13}C NMR (101 MHz, CDCl$_3$):** δ 155.3, 82.9, 79.0, 55.5, 46.0, 44.2, 36.7, 36.0, 28.7, 28.7, 25.5, 25.5, 25.1, 25.0, 24.1, 21.8; **HRMS (ESI+):** Calculated for $C_{21}H_{40}O_4NBNa$ [M+Na]$^+$: 404.2943, Found: 404.2944.

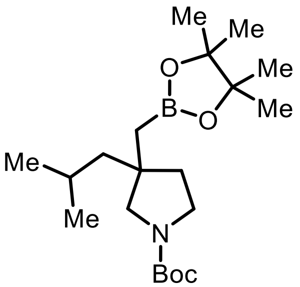

***Tert*-butyl 3-isobutyl-3-((4,4,5,5-tetramethyl-1,3,2-dioxaborolan-2-yl)methyl)pyrrolidine-1-carboxylate (5):** The title compound was prepared according to General Procedure A. NMR yield of the unpurified reaction mixture was determined using 1,3,5-trimethylbenzene as an NMR standard. Hydrogenation was performed using MeOH as solvent. Purification for both the steps by silica gel column chromatography (Gradient: 6% to 12% EtOAc:hexanes) yields **5** as a colorless oil.

Average over 2 runs: 99% NMR yield (alkenylboration step), 88% isolated yield (over 2 steps).

IR (neat): 2975 (w), 2955 (w), 2931 (w), 2871 (w), 1694 (s), 1479 (w), 1398 (s), 1363 (s), 1321 (m), 1251 (w), 1159 (m), 1141 (s), 1109 (s), 969 (m), 885 (w), 848 (m), 772 (m) cm^{-1}; **^{1}H NMR (400 MHz, $CDCl_3$):** δ 3.32 – 3.14 (m, 3H), 3.05 – 2.97 (m, 1H), 1.78 – 1.73 (m, 1H), 1.69 – 1.56 (m, 2H), 1.39 (s, 9H), 1.32 – 1.23 (m, 2H), 1.17 (s, 12H), 0.89 (s, 2H), 0.86 (d, *J* = 2.0 Hz, 3H), 0.83 (d, *J* = 4.6 Hz, 3H); **^{13}C NMR (101 MHz, $CDCl_3$, *mixture of rotamers*):** δ 154.9, 154.8, 82.9, 82.9, 78.7, 58.9, 47.8, 47.6, 44.5, 44.3, 43.4, 42.5, 38.8, 38.1, 28.6, 25.1, 24.9, 24.9, 24.5, 24.1; **HRMS (ESI+):** Calculated for $C_{40}H_{76}O_8N_2B_2Na$ [2M+Na]$^+$: 757.5680, Found: 757.5697.

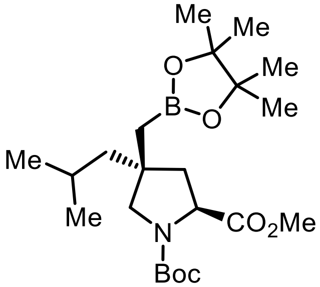

1-(*Tert*-butyl) 2-methyl (2*S*,4*R*)-4-isobutyl-4-((4,4,5,5-tetramethyl-1,3,2-dioxaborolan-2-yl)methyl)pyrrolidine-1,2-dicarboxylate (6): The title compound was prepared according to General Procedure A. Hydrogenation was performed using MeOH as solvent. Purification for both the steps by silica gel column chromatography (Gradient: 16% to 20% EtOAc:hexanes) yields **6** as a colorless oil.

Average over 2 runs: 49% isolated yield (over 2 steps), >20:1 dr.

IR (neat): 2976 (w), 2956 (w), 2929 (w), 1755 (m), 1703 (s), 1396 (s), 1366 (s), 1326 (w), 1205 (m), 1165 (m), 1144 (m), 1114 (m), 969(w), 847 (w) cm^{-1}; **1H NMR (400 MHz, $CDCl_3$, *ratio of rotamers 60:40*):** δ 4.25 (t, *J* = 8.2 Hz, 1H *minor*), 4.15 (t, *J* = 8.2 Hz, 1H, *major*), 3.68 (s, 3H), 3.49 (d, *J* = 10.8 Hz, 1H, *major*), 3.44 (d, *J* = 10.7 Hz, 1H, *minor*), 3.20 (d, *J* = 10.8 Hz, 1H, *major*), 3.12 (d, *J* = 10.7 Hz, 1H, *minor*), 2.22 – 2.14 (m, 1H), 1.83-1.76 (m, 1H), 1.65 – 1.59 (m, 1H), 1.42 (s, 9H, *minor*), 1.37 (s, 9H, *major*), 1.29 (d, *J* = 6.0 Hz, 2H), 1.20 (s, 12H, *minor*), 1.18 (s, 12H, *major*), 1.06 – 0.94 (m, 2H), 0.89 (d, *J* = 6.9 Hz, 3H), 0.87 (d, *J* = 6.9 Hz, 3H); **^{13}C NMR (101 MHz, $CDCl_3$, *mixture of rotamers*):** δ 174.0, 173.7, 154.8, 154.0, 83.2, 83.2, 79.8, 79.8, 58.8, 58.8, 58.3, 52.1, 51.9, 46.4, 46.3, 43.5, 43.4, 42.9, 42.6, 36.8, 28.5, 28.4, 25.2, 25.1, 25.1, 24.9, 24.9, 24.8, 24.7; **HRMS (ESI+):** Calculated for $C_{22}H_{40}O_6NBNa$ [M+Na]$^+$: 448.2841, Found: 448.2843.

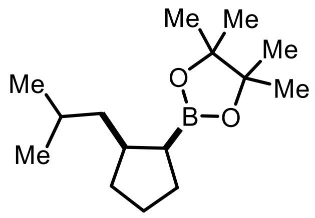

2-(2-Isobutylcyclopentyl)-4,4,5,5-tetramethyl-1,3,2-dioxaborolane (7): The title compound was prepared according to a modified version of General Procedure A using 1.0 equiv. of vinyl bromide and 1.5 equiv. of alkene NMR yield of the unpurified reaction mixture was determined using 1,3,5-trimethylbenzene as an NMR standard. Hydrogenation was performed using MeOH as solvent. Purification for both the steps by silica gel column chromatography (Gradient: hexanes to 20:1 hexanes:Et_2O) yields **7** as a colorless oil.

Average over 2 runs: 55% NMR yield (alkenylboration step), 49% isolated yield (over 2 steps), >20:1 d.r.

IR (neat): 2977 (m), 2867 (m), 1467 (m), 1311 (m), 1143 (s), 972 (m), 854 (m) cm^{-1}; **^{1}H NMR (600 MHz, $CDCl_3$):** δ 2.13 – 2.07 (m, 1H), 1.74 – 1.53 (m, 5H), 1.52 – 1.47 (m, 1H), 1.44 –1.38 (m, 1H), 1.31 – 1.13 (m, 15H), 0.87 (d, J = 6.0 Hz, 3H), 0.86 (d, J = 6.3 Hz, 3H); **^{13}C NMR (101 MHz, $CDCl_3$):** δ 82.8, 43.4, 40.4, 32.6, 27.5, 27.3, 27.0, 25.3, 25.1, 25.0, 23.5, 22.5; **HRMS (EI+):** Calculated for $C_{15}H_{29}BO_2$ $[M]^+$: 252.2261, Found: 252.2257.

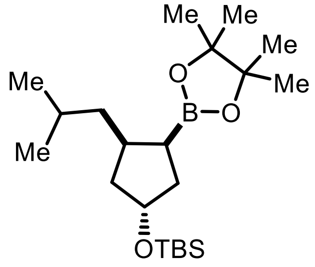

***Tert*-butyl((3-isobutyl-4-(4,4,5,5-tetramethyl-1,3,2-dioxaborolan-2-yl)cyclopentyl)oxy) dimethylsilane (8):** The title compound was prepared according to a modified version of General Procedure A using 1.0 equiv. of vinyl bromide and 1.5 equiv. of alkene. After the alkenylboration, the reaction mixture was taken in 20 mL EtOAc and washed with brine. NMR yield of the unpurified reaction mixture was determined using 1,3,5-trimethylbenzene as an NMR standard. Hydrogenation was performed using EtOAc as solvent. Purification for both the steps by silica gel column chromatography (gradient: hexanes to 20:1 hexanes:Et_2O) yields **8** as a colorless oil.

Average over 2 runs: 75% NMR yield (alkenylboration step), 67% isolated yield (over 2 steps), >20:1 d.r.

IR (neat): 2952 (s), 2897 (m), 1468 (m), 1369 (m), 1104 (s), 832 (s), 772 (s) cm^{-1}; **^{1}H NMR (400 MHz, $CDCl_3$):** δ 4.36 – 4.32 (m, 1H), 2.45 – 2.35 (m, 1H), 1.88 – 1.82 (m, 1H), 1.70 – 1.50 (m, 4H), 1.48 – 1.37 (m, 1H), 1.29 – 1.22 (m, 13H), 1.16 – 1.11 (m, 1H), 0.89 – 0.83 (m, 15H), 0.02 (s, 6H); **^{13}C NMR (101 MHz, $CDCl_3$):** δ 82.9, 74.3, 43.8, 42.8, 38.2, 37.6, 26.9, 26.1, 25.2, 25.0, 23.5, 22.3, 18.3, -4.5, -4.5; **HRMS (APCI+):** Calculated for $C_{21}H_{44}O_3BSi$ $[M+H]^+$: 383.3147, Found: 383.3150.

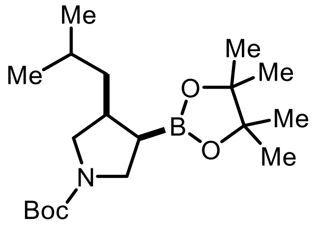

***Tert*-butyl 3-isobutyl-4-(4,4,5,5-tetramethyl-1,3,2-dioxaborolan-2-yl)pyrrolidine-1-carboxylate (9):** The title compound was prepared according to General Procedure A. NMR yield of the unpurified reaction mixture was determined using 1,3,5-trimethylbenzene as an NMR standard. Hydrogenation was performed using MeOH as solvent. Purification for both the steps by silica gel column chromatography (Gradient: 10% to 12% EtOAc:hexanes) yields **9** as a colorless oil.

Average over 2 runs: 94% NMR yield (alkenylboration step), 84% isolated yield (over 2 steps), >20:1 d.r.

IR (neat): 2976 (w), 2957 (w), 2931 (w), 2870 (w), 1695 (s), 1379 (s), 1325 (s), 1253 (w), 1233 (w), 1165 (m), 1143 (s), 1104 (m), 974 (w), 867 (w), 770 (w) cm^{-1}; **^{1}H NMR (400 MHz, $CDCl_3$):** δ 3.34 (d, J = 7.4 Hz, 2H), 3.28 (dd, J = 10.4, 6.5 Hz, 1H), 3.08 (dd, J = 11.0, 5.7 Hz, 1H), 2.33 – 2.25 (m, 1H), 1.65 – 1.59 (m, 1H), 1.57 – 1.49 (m, 1H), 1.41 (s, 9H), 1.19 (d, J = 3.5 Hz, 13H), 1.15 – 1.08 (m, 1H), 0.84 (d, J = 6.6 Hz, 6H); **^{13}C NMR (101 MHz, $CDCl_3$):** δ 154.9, 83.4, 78.8, 51.3, 47.1, 40.5, 38.3, 28.7, 26.7, 25.0, 24.8, 23.3, 22.3; **HRMS (ESI+):** Calculated for $C_{19}H_{36}O_4NBNa$ $[M+Na]^+$: 376.2641, Found: 376.2631.

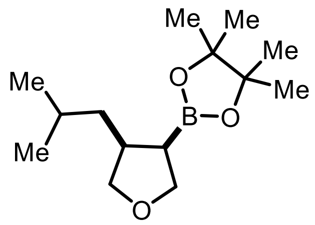

2-(4-Isobutyltetrahydrofuran-3-yl)-4,4,5,5-tetramethyl-1,3,2-dioxaborolane (10): The title compound was prepared according to General Procedure A. After the alkenylboration, the reaction mixture was taken in 20 mL EtOAc and washed with brine. NMR yield of the unpurified reaction mixture was determined using 1,3,5-trimethylbenzene as an NMR standard. Hydrogenation was performed using EtOAc as solvent. Purification for both the steps by silica gel column chromatography (Gradient: 7% to 9% EtOAc: hexanes) yields **10** as a colorless oil.

Average over 2 runs: 74% NMR yield (alkenylboration step), 58% isolated yield (over 2 steps), >20:1 d.r.

IR (neat): 2976 (w), 2957 (w), 2930 (w), 2869 (w), 1468 (m), 1381 (s), 1371 (s), 1317 (s), 1142 (s), 974 (w), 850 (m) cm^{-1}; **^{1}H NMR (400 MHz, $CDCl_3$):** δ 3.91 (t, *J* = 8.1 Hz, 1H), 3.81 – 3.79 (m, 1H), 3.77 – 3.75 (m, 1H), 3.45 (dd, *J* = 8.0, 5.8 Hz, 1H), 2.42 (ddt, *J* = 14.7, 8.5, 6.2 Hz, 1H), 1.72 (dd, *J* = 16.4, 8.2 Hz, 1H), 1.58 – 1.48 (m, 1H), 1.23 (d, *J* = 2.4 Hz, 14H), 0.87 (d, *J* = 3.1 Hz, 3H), 0.85 (d, *J* = 3.0 Hz, 3H); **^{13}C NMR (126 MHz, $CDCl_3$):** δ 83.4, 73.2, 69.9, 40.3, 39.7, 27.0, 25.1, 24.9, 23.5, 22.1; **HRMS (APCI+):** Calculated for $C_{14}H_{28}O_3B$ $[M+H]^+$: 255.2126, Found: 255.2128.

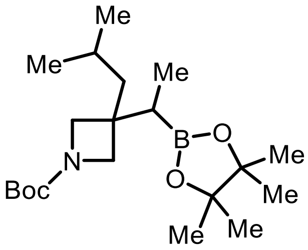

***Tert*-butyl-3-isobutyl-3-(1-(4,4,5,5-tetramethyl-1,3,2-dioxaborolan-2-yl)ethyl)azetidine-1-carboxylate (11):** The title compound was prepared according to a modified version of General Procedure A using 3.0 equiv. of vinyl bromide, 3.0 equiv. of NaO^tBu and 4.0 equiv. of B_2pin_2 in 4:1 THF:DMA. NMR yield of the unpurified reaction mixture was determined using 1,3,5-trimethylbenzene as an NMR standard. Hydrogenation was performed using MeOH as solvent.

Purification for both the steps by silica gel column chromatography (Gradient: 6% to 10% EtOAc:hexanes) yields **11** as a colorless oil.

Average over 2 runs: 99% NMR yield (alkenylboration step), 94% isolated yield (over 2 steps), >10:1 rr.

IR (neat): 2975 (s), 2874 (m), 1700 (s), 1457 (s), 1320 (m), 1142 (s), 855 (m), 731 (s) cm^{-1}; **^{1}H NMR (600 MHz, $CDCl_3$):** δ 3.84 (d, *J* = 8.4 Hz, 1H), 3.78 (d, *J* = 8.4 Hz, 1H), 3.57 – 3.49 (m, 2H), 1.68 (hept, *J* = 6.7 Hz, 1H), 1.57 (dd, *J* = 14.2, 6.7 Hz, 1H), 1.47 – 1.42 (m, 10H), 1.33 (q, *J* = 7.4 Hz, 1H), 1.23 (s, 12H), 0.95 (d, *J* = 7.4 Hz, 3H), 0.88 (d, *J* = 5.3 Hz, 3H), 0.87 (d, *J* = 5.3 Hz, 3H); **^{13}C NMR (126 MHz, $CDCl_3$):** δ 156.4, 83.2, 78.9, 46.3, 42.8, 38.2, 28.6, 28.6, 25.1, 24.9, 24.8, 23.6, 10.5; **HRMS (APCI+):** Calculated for $C_{20}H_{39}O_4NB$ $[M+H]^+$: 368.2967, Found: 368.2974.

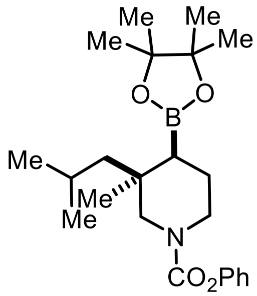

Phenyl 3-isobutyl-3-methyl-4-(4,4,5,5-tetramethyl-1,3,2-dioxaborolan-2-yl)piperidine-1-carboxylate (12): The title compound was prepared according to a modified version of General Procedure A using 3.0 equiv. of vinyl bromide, 3.0 equiv. of NaO^{t}Bu and 4.0 equiv. of B_2pin_2 in 4:1 THF:DMA. NMR yield of the unpurified reaction mixture was determined using 1,3,5-trimethylbenzene as an NMR standard. Hydrogenation was performed using MeOH as solvent. Purification for both the steps by silica gel column chromatography (Gradient: 6% to 10% EtOAc:hexanes) yields **12** as a colorless oil.

Average over 2 runs: 66% NMR yield (alkenylboration step), 54% isolated yield (over 2 steps).

IR (neat): 2976 (w), 2954 (w), 2929 (w), 2360 (w), 2160 (m), 2031 (m), 1977 (m), 1720 (s), 1458 (m), 1372 (w), 1205 (s), 1143 (m), 1067 (m), 994 (w), 848 (w), 669 (w) cm^{-1}; **^{1}H NMR (400 MHz, $CDCl_3$, *mixture of rotamers*):** δ 7.34 (t, *J* = 7.8 Hz, 2H), 7.17 (t, *J* = 7.5 Hz, 1H), 7.08 (d, *J* = 7.8 Hz, 2H), 3.95 – 3.73 (m, 2H), 3.29 – 3.17 (m, 1H), 2.96 (d, *J* = 13.2 Hz, 1H, *major*), 2.83 (d, *J* = 13.1 Hz, 1H, *minor*), 1.78 – 1.67 (m, 3H), 1.47 – 1.41 (m, 1H), 1.25 (s, 12H), 1.20 – 1.15 (m, 1H), 1.08 – 1.02 (m, 4H), 0.98 – 0.92 (m, 6H); **^{13}C NMR (101 MHz, $CDCl_3$, *mixture of rotamers*):** δ

154.3, 151.8, 129.3, 125.1, 125.0, 121.9, 121.8, 83.2, 54.2, 53.7, 44.8, 44.5, 44.4, 44.3, 35.5, 25.6, 25.6, 25.0, 24.9, 24.4, 24.3, 24.1, 23.5, 22.9; **HRMS (APCI+):** Calculated for $C_{23}H_{37}O_4NB$ $[M+H]^+$: 402.2810, Found: 402.2814.

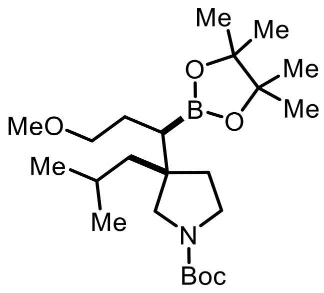

***Tert*-butyl-3-isobutyl-3-(3-methoxy-1-(4,4,5,5-tetramethyl-1,3,2-dioxaborolan-2-yl)propyl) pyrrolidine-1-carboxylate (13):** The title compound was prepared according to a modified version of General Procedure A using 3.0 equiv. of vinyl bromide, 3.0 equiv. of NaO^{t}Bu and 4.0 equiv. of B_2pin_2 in 4:1 THF:DMA. Hydrogenation was performed using MeOH as solvent. Purification for both the steps by silica gel column chromatography (Gradient: 15% to 18% EtOAc:hexanes) yields **13** as a colorless oil.

Average over 2 runs: 69% isolated yield (over 2 steps), >20:1 d.r.

IR (neat): 2975 (w), 2954 (w), 2929 (w0, 2870 (w), 1694 (s), 1453 (m), 1391 (s), 1365 (s), 1320 (m), 1143 (s), 1109 (s), 967 (w), 882 (m), 851 (m), 771 (w) cm^{-1}; **^{1}H NMR (400 MHz, $CDCl_3$, *mixture of rotamers*):** δ 3.39 – 3.26 (m, 7H), 3.22 – 3.11 (m, 2H), 1.96 – 1.87 (m, 1H), 1.73 – 1.55 (m, 4H), 1.41 (s, 9H), 1.37 – 1.33 (m, 1H), 1.29 – 1.25 (m, 1H), 1.21 (s, 12H), 1.13 (dd, J = 12.1, 2.8 Hz, 1H), 0.87 (d, J = 6.6 Hz, 6H); **^{13}C NMR (126 MHz, $CDCl_3$, *mixture of rotamers*):** δ 154.9, 154.8, 83.2, 83.2, 78.9, 78.8, 73.3, 58.6, 58.5, 56.1, 55.4, 46.9, 46.0, 45.4, 45.3, 45.1, 44.7, 35.1, 34.7, 28.7, 27.6, 27.4, 25.1, 25.1, 24.6, 24.6; **HRMS (APCI+):** Calculated for $C_{23}H_{45}O_5NB$ $[M+H]^+$: 426.3385, Found: 426.3388.

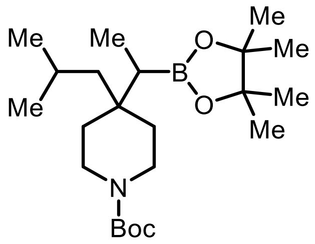

***Tert*-butyl 4-isobutyl-4-(1-(4,4,5,5-tetramethyl-1,3,2-dioxaborolan-2-yl)ethyl)piperidine-1-carboxylate (14):** The title compound was prepared according to a modified version of General

Procedure A using 3.0 equiv. of vinyl bromide, 3.0 equiv. of NaO^{t}Bu and 4.0 equiv. of B_2pin_2 in 4:1 THF:DMA. NMR yield of the unpurified reaction mixture was determined using 1,3,5-trimethylbenzene as an NMR standard. Hydrogenation was performed using MeOH as solvent. Purification for both the steps by silica gel column chromatography (Gradient: 6% to 10% EtOAc:hexanes) yields **14** as a colorless oil.

Average over 2 runs: 68% NMR yield (alkenylboration step), 58% isolated yield (over 2 steps).

IR (neat): 2975 (w), 2953 (w), 2930 (w), 1693 (s), 1468 (m), 1379 (m), 1242 (m), 1145 (s), 978 (w), 867 (w) cm^{-1}; **^{1}H NMR (400 MHz, $CDCl_3$):** δ 3.51 (s, 1H), 3.46 – 3.40 (m, 1H), 3.25 – 3.19 (m, 2H), 1.69 – 1.57 (m, 2H), 1.42 (bs, 11H), 1.39 – 1.34 (m, 2H), 1.31 – 1.29 (m, 2H), 1.20 (s, 12H), 0.89 (d, J = 6.6 Hz, 6H), 0.84 (d, J = 7.5 Hz, 3H); **^{13}C NMR (126 MHz, $CDCl_3$):** δ 155.2, 82.9, 79.0, 43.0, 40.3, 39.4, 36.2, 33.1, 28.6, 25.7, 25.7, 24.9, 24.9, 23.4, 9.8; **HRMS (APCI+):** Calculated for $C_{22}H_{43}O_4NB$ [M+H]$^+$: 396.3280, Found: 396.3280.

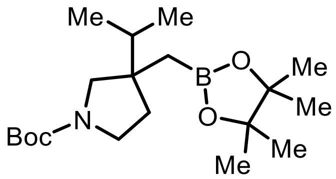

***Tert*-butyl 3-isopropyl-3-((4,4,5,5-tetramethyl-1,3,2-dioxaborolan-2-yl)methyl)pyrrolidine-1-carboxylate (22):** The title compound was prepared according to General Procedure A. NMR yield of the unpurified reaction mixture was determined using 1,3,5-trimethylbenzene as an NMR standard. Hydrogenation was performed using MeOH as solvent. Purification for both the steps by silica gel column chromatography (Gradient: 6% to 10% EtOAc:hexanes) yields **22** as a colorless oil.

Average over 2 runs: 44% NMR yield (alkenylboration step), 36% isolated yield (over 2 steps).

IR (neat): 2974 (w), 2933 (w), 2877 (w), 1696 (s), 1479 (w), 1397 (s), 1363 (s), 1324 (m), 1167 (m), 1144 (s), 1110 (m), 970 (w), 882 (w), 858 (w), 772 (w) cm^{-1}; **^{1}H NMR (600 MHz, $CDCl_3$):** δ 3.37 (s, 2H), 3.25 (d, J = 10.8 Hz, 1H), 3.11 – 2.94 (m, 1H), 1.85 (dt, J = 11.6, 5.5 Hz, 1H), 1.69 – 1.61 (m, 2H), 1.43 (s, 9H), 1.20 (d, J = 4.0 Hz, 12H), 0.86 (dd, J = 6.9, 1.7 Hz, 6H), 0.83 (s, 2H); **^{13}C NMR (126 MHz, $CDCl_3$):** δ 155.0, 83.0, 78.8, 57.1, 46.9, 45.3, 36.4, 35.9, 28.7, 25.0, 24.9, 18.7, 18.5; **HRMS (ESI+):** Calculated for $C_{19}H_{36}O_4NBNa$ [M+Na]$^+$: 376.2630, Found: 376.2633.

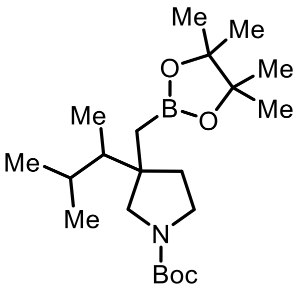

***Tert*-butyl 3-(3-methylbutan-2-yl)-3-((4,4,5,5-tetramethyl-1,3,2-dioxaborolan-2-yl)methyl) pyrrolidine-1-carboxylate (23):** The title compound was prepared according to General Procedure A. Hydrogenation was performed using MeOH as solvent. Purification by silica gel column chromatography (Gradient: 6% to 10% EtOAc:hexanes) yields **23** as a colorless oil. Diastereomeric ratio was determined by ^{19}F NMR analysis of the 2-fluorobenzoate ester of the corresponding alcohol.

Average over 2 runs: 74% isolated yield (over 2 steps), 11:1 dr.

IR (neat): 2975 (m), 2932 (w), 2876 (w), 1695 (s), 1479 (w), 1453 (w), 1391 (s), 1364 (s), 1322 (m), 1144 (m), 1109 (s), 970 (w), 882 (w), 848 (w), 772 (w) cm^{-1}; **^{1}H NMR (400 MHz, $CDCl_3$):** δ 3.34 – 3.08 (m, 4H), 1.92 – 1.66 (m, 3H), 1.41 (bs, 10H), 1.19 (s, 12H), 1.13 – 0.91 (m, 2H), 0.86 (dd, *J* = 6.9, 1.4 Hz, 3H), 0.79 – 0.73 (m, 6H); **^{13}C NMR (126 MHz, $CDCl_3$, *mixture of rotamers and diastereomers*):** δ 155.0, 83.0, 78.8, 57.5, 56.3, 55.5, 47.8, 47.1, 46.7, 45.7, 45.5, 45.3, 44.9, 44.8, 44.5, 36.1, 36.0, 35.7, 34.9, 28.7, 28.5, 25.0, 25.0, 24.9, 24.9, 24.2, 24.0, 18.1, 17.8, 17.4, 9.7, 9.5, 8.9; **HRMS (ESI+):** Calculated for $C_{21}H_{40}O_4NBNa$ $[M+Na]^+$: 404.2946, Found: 404.2945.

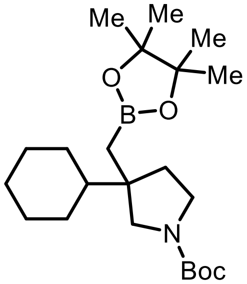

***Tert*-butyl 3-cyclohexyl-3-((4,4,5,5-tetramethyl-1,3,2-dioxaborolan-2-yl)methyl)pyrrolidine-1-carboxylate (24):** The title compound was prepared according to General Procedure A. NMR yield of the unpurified reaction mixture was determined using 1,3,5-trimethylbenzene as an NMR standard. Hydrogenation was performed using MeOH as solvent. Purification for both the steps by

silica gel column chromatography (Gradient: 4% to 14% EtOAc:hexanes) yields **24** as a colorless oil.

Average over 2 runs: 99% NMR yield (alkenylboration step), 80% isolated yield (over 2 steps).

IR (neat): 2976 (w), 2926 (m), 2853 (w), 1695 (s), 1479 (w), 1393 (s), 1362 (s), 1170 (w), 1144 (m), 1109 (s), 970 (w), 882 (w), 848 (w), 772 (w) cm^{-1}; **^{1}H NMR (400 MHz, CDCl$_3$):** δ 3.37 (dd, *J* = 9.2, 5.3 Hz, 2H), 3.27 (d, *J* = 10.7 Hz, 1H), 3.07 (d, *J* = 10.7 Hz, 1H), 1.91 – 1.85 (m, 1H), 1.76 – 1.73 (m, 2H), 1.70 – 1.57 (m, 5H), 1.45 (s, 9H), 1.22 (d, *J* = 2.4 Hz, 12H), 1.18 – 1.00 (m, 5H), 0.86 (s, 2H); **^{13}C NMR (126 MHz, CDCl$_3$, *mixture of rotamers*):** δ 155.0, 154.8, 82.9, 78.8, 57.3, 57.0, 47.7, 47.3, 47.0, 46.1, 45.4, 44.9, 36.1, 35.6, 29.0, 28.7, 28.6, 27.0, 27.0, 26.6, 25.0, 24.9; **HRMS (APCI+):** Calculated for $C_{22}H_{41}O_4NB$ [M+H]$^+$: 394.3123, Found: 394.3123.

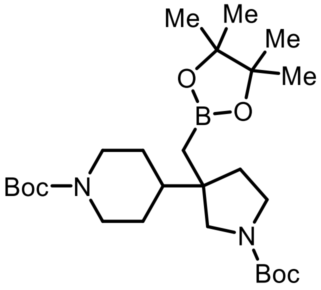

***Tert*-butyl 4-(1-(tert-butoxycarbonyl)-3-((4,4,5,5-tetramethyl-1,3,2-dioxaborolan-2-yl)methyl)pyrrolidin-3-yl)piperidine-1-carboxylate (25):** The title compound was prepared according to General Procedure A. NMR yield of the unpurified reaction mixture was determined using 1,3,5-trimethylbenzene as an NMR standard. Hydrogenation was performed using MeOH as solvent. Purification for both the steps by silica gel column chromatography (Gradient: 20% to 40% EtOAc:hexanes) yields **25** as a colorless oil.

Average over 2 runs: 65% NMR yield (alkenylboration step), 44% isolated yield (over 2 steps).

IR (neat): 2976 (w), 2931 (w), 2866 (w), 1692 (s), 1401 (s), 1364 (s), 1242 (m), 1168 (s), 1144 (s), 970 (w), 881 (w), 848 (w), 772 (w) cm^{-1}; **^{1}H NMR (500 MHz, CDCl$_3$):** δ 4.11 – 4.06 (m, 2H), 3.41 – 3.33 (m, 2H), 3.22 – 3.04 (m, 2H), 2.55 (t, *J* = 12.9 Hz, 2H), 1.83 – 1.78 (m, 1H), 1.67 – 1.61 (m, 1H), 1.57 – 1.55 (m, 1H), 1.52 – 1.48 (m, 1H), 1.40 (s, 18H), 1.36 – 1.29 (m, 1H), 1.27 – 1.20 (m, 2H), 1.17 (s, 12H), 0.83 (s, 2H); **^{13}C NMR (126 MHz, CDCl$_3$, *mixture of rotamers*):** δ 154.8, 83.1, 79.3, 79.0, 56.6, 46.3, 45.3, 44.9, 44.4, 35.4, 28.6, 28.5, 27.8, 27.7, 24.7, 24.9; **HRMS (ESI+):** Calculated for $C_{26}H_{47}O_6N_2BNa$ [M+Na]$^+$: 517.3419, Found: 517.3415.

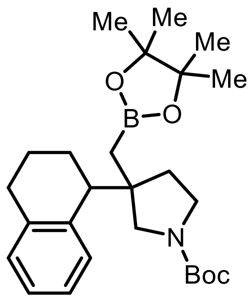

***Tert*-butyl 3-(1,2,3,4-tetrahydronaphthalen-1-yl)-3-((4,4,5,5-tetramethyl-1,3,2-dioxaborolan-2-yl)methyl)pyrrolidine-1-carboxylate (26):** The title compound was prepared according to a modified version of General Procedure A using 3.0 equiv. of vinyl bromide, 3.0 equiv. of NaO^{t}Bu and 4.0 equiv. of B_2pin_2 in 4:1 THF:DMA. NMR yield of the unpurified reaction mixture was determined using 1,3,5-trimethylbenzene as an NMR standard. Hydrogenation was performed using MeOH as solvent. Purification for both the steps by silica gel column chromatography (Gradient: 8% to 14% EtOAc:hexanes) yields **26** as a colorless oil.

Average over 2 runs: 83% NMR yield (alkenylboration step), 71% isolated yield (over 2 steps), 2:1 d.r.

IR (neat): 2975 (w), 2932 (w), 2883 (w), 1694 (s), 1479 (w), 1451 (w), 1398 (s), 1364 (s), 1170 (m), 1143 (m), 1117 (m), 970 (w), 884 (w), 848 (w), 771 (w), cm^{-1}; **^{1}H NMR (400 MHz, $CDCl_3$, *mixture of diastereomers*):** δ 7.18 – 7.03 (m, 4H), 3.45 – 3.10 (m, 4H), 3.06 – 3.01 (m, 1H), 2.73 – 2.63 (m, 2H), 1.99 – 1.79 (m, 5H), 1.55 – 1.48 (m, 1H), 1.45 (s, 9H, *major*), 1.43 (s, 9H, *minor*), 1.21 (d, J = 3.8 Hz, 12H, *minor*), 1.18 (d, J = 2.4 Hz, 12H, *major*), 0.95 – 0.74 (m, 2H); **^{13}C NMR (101 MHz, $CDCl_3$, *mixture of diastereomers and rotamers*):** δ 155.0, 154.9, 154.8, 154.7, 140.1, 140.0, 139.7, 137.8, 137.6, 130.7, 130.5, 130.3, 129.1, 129.0, 129.0, 128.9, 125.9, 125.9, 125.2, 125.0, 83.0, 78.8, 78.8, 57.4, 57.1, 56.3, 49.3, 49.0, 48.3, 47.9, 45.5, 45.3, 45.1, 45.1, 44.9, 44.0, 43.8, 37.4, 37.0, 35.6, 35.1, 30.0, 29.9, 29.6, 28.7, 26.2, 26.0, 25.1, 25.0, 24.9, 24.9, 24.8, 22.0, 21.9, 21.4; **HRMS (ESI+):** Calculated for $C_{26}H_{40}O_4NBNa$ $[M+Na]^+$: 464.2947, Found: 464.2938.

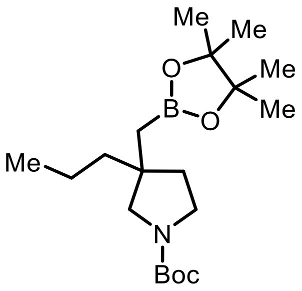

***Tert*-butyl 3-propyl-3-((4,4,5,5-tetramethyl-1,3,2-dioxaborolan-2-yl)methyl)pyrrolidine-1-carboxylate (27):** The title compound was prepared according to General Procedure A. NMR yield of the unpurified reaction mixture was determined using 1,3,5-trimethylbenzene as an NMR standard. Hydrogenation was performed using MeOH as solvent. Purification for both the steps by silica gel column chromatography (Gradient: 6% to 10% EtOAc:hexanes) yields **27** as a colorless oil.

Average over 2 runs: 70% NMR yield (alkenylboration step), 57% isolated yield (over 2 steps).

IR (neat): 2976 (w), 2931 (w), 2872 (w), 1696 (s), 1479 (w), 1454 (w), 1399 (s), 1364 (s), 1324 (w), 1216 (w), 1168 (m), 1145 (m), 1106 (s), 969 (w), 883 (w), 848 (w), 773 (w) cm^{-1}; **^{1}H NMR (400 MHz, CDCl$_3$):** δ 3.31 (s, 2H), 3.13 – 3.06 (m, 2H), 1.75 – 1.61 (m, 2H), 1.42 (s, 9H), 1.37 – 1.31 (m, 2H), 1.30 – 1.24 (m, 2H), 1.19 (s, 12H), 0.89 (s, 2H), 0.86 (t, J = 6.9 Hz, 3H); **^{13}C NMR (101 MHz, CDCl$_3$, *mixture of rotamers*):** δ 155.0, 83.0, 78.8, 58.2, 57.9, 45.0, 44.6, 43.3, 42.5, 41.3, 38.1, 37.3, 28.7, 24.9, 24.9, 24.7, 18.30, 14.9; **HRMS (APCI+):** Calculated for $C_{19}H_{37}O_4NB$ $[M+H]^+$: 354.2810, Found: 354.2811.

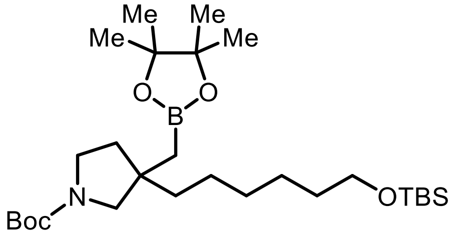

***Tert*-butyl 3-(6-((*tert*-butyldimethylsilyl)oxy)hexyl)-3-((4,4,5,5-tetramethyl-1,3,2-dioxaborolan-2-yl)methyl)pyrrolidine-1-carboxylate (28):** The title compound was prepared according to General Procedure A. After the alkenylboration, the reaction mixture was taken in 20 mL EtOAc and washed with brine. Hydrogenation was performed using EtOAc as solvent. Purification for both the steps by silica gel column chromatography (gradient: 4% to 14% EtOAc:hexanes) yields **28** as a colorless oil.

Average over 2 runs: 55% isolated yield (over 2 steps).

IR (neat): 2976 (w), 2928 (m), 2893 (w), 2857 (m), 1696 (s), 1462 (w), 1391 (s), 1363 (s), 1323 (m), 1166 (s), 1144 (s), 1102 (s), 881 (w), 835 (m), 773 (m) cm^{-1}; **^{1}H NMR (400 MHz, $CDCl_3$):** δ 3.59 (t, *J* = 6.6 Hz, 2H), 3.40 – 3.29 (m, 2H), 3.13 (s, 2H), 1.78 – 1.64 (m, 2H), 1.52 – 1.48 (m, 2H), 1.45 (s, 9H), 1.40 – 1.37 (m, 2H), 1.31 – 1.26 (m, 6H), 1.22 (s, 12H), 0.92 (s, 2H), 0.89 (s, 9H), 0.04 (s, 6H); **^{13}C NMR (126 MHz, $CDCl_3$, *mixture of rotamers*):** δ 154.9, 83.0, 78.8, 63.3, 58.2, 57.9, 44.9, 44.6, 43.2, 42.4, 38.8, 38.1, 37.2, 32.9, 30.3, 28.6, 26.1, 25.8, 25.0, 24.9, 24.9, 18.4, -5.2; **HRMS (ESI+):** Calculated for $C_{28}H_{56}O_5NBNaSi$ $[M+Na]^+$: 548.3918, Found: 548.3907.

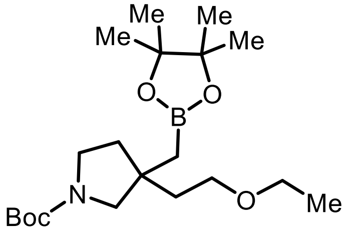

***Tert*-butyl 3-(2-ethoxyethyl)-3-((4,4,5,5-tetramethyl-1,3,2-dioxaborolan-2-yl)methyl) pyrrolidine-1-carboxylate (31):** The title compound was prepared according to General Procedure A. After the alkenylboration, the reaction mixture was taken in 20 mL EtOAc and washed with brine. Hydrogenation was performed using MeOH as solvent. Purification for both the steps by silica gel column chromatography (gradient: 7% to 14% EtOAc:hexanes) yields **31** as a colorless oil.

Average over 2 runs: 71% isolated yield (over 2 steps).

IR (neat): 2975 (w), 2930 (w), 2866 (w), 1693 (s), 1398 (s), 1363 (s), 1319 (m), 1167 (m), 1143 (s), 1105 (s), 968 (m), 880 (m), 847 (m), 771 (w) cm^{-1} ; **^{1}H NMR (400 MHz, $CDCl_3$):** δ 3.49 – 3.42 (m, 4H), 3.35 (t, *J* = 7.2 Hz, 2H), 3.17 (s, 2H), 1.79 – 1.72 (m, 4H), 1.45 (s, 9H), 1.23 (s, 12H), 1.18 (t, *J* = 7.0 Hz, 3H), 0.94 (s, 2H); **^{13}C NMR (126 MHz, $CDCl_3$, *mixture of rotamers*):** δ 154.9, 83.1, 78.9, 67.7, 66.2, 58.3, 44.6, 41.6, 41.2, 38.3, 37.9, 28.6, 24.9, 24.9, 19.6, 15.3; **HRMS (APCI+):** Calculated for $C_{20}H_{39}O_5NB$ $[M+H]^+$: 384.2916, Found: 384.2919.

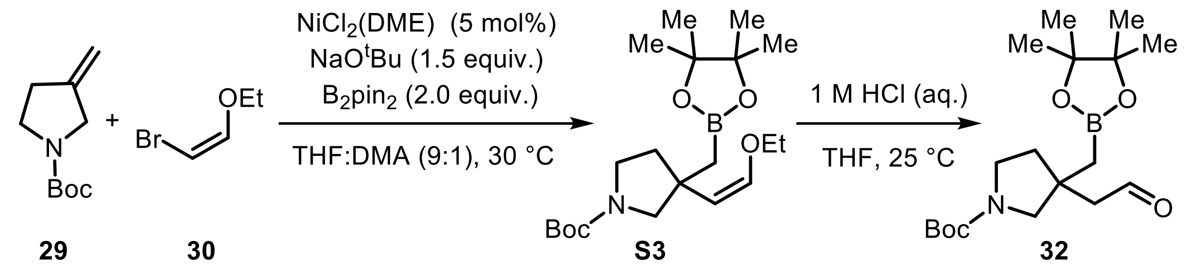

***Tert*-butyl 3-(2-oxoethyl)-3-((4,4,5,5-tetramethyl-1,3,2-dioxaborolan-2-yl)methyl) pyrrolidine-1-carboxylate (32):** In an N_2-filled glovebox, to an oven-dried 13 x 100 mm screw-capped vial was added bis(pinacolato)diboron (152 mg, 0.600 mmol, 2.00 equiv.) and sodium *tert*-butoxide (43 mg, 0.45 mmol, 1.5 equiv.). In a separate oven-dried 2-dram vial, Ni(DME)Cl_2 (8.20 mg, 0.0375 mmol) was added. Both vials were sealed with septa and removed from the glovebox. THF (2.7 mL) was added to the reaction vial, followed by *tert*-butyl 3-methylenepyrrolidine-1-carboxylate **29** (55 µL, 0.3 mmol, 1.0 equiv.) and (*Z*)-1-bromo-2-ethoxyethene **30** (48 µL, 0.45 mmol, 1.5 equiv.). DMA (0.75 mL) was then added to the vial containing Ni(DME)Cl_2 (0.05 M in Ni(DME)Cl_2) to prepare the catalyst solution. The catalyst solution (0.30 mL) was then added to the reaction vial (5 mol% catalyst loading). The septum was then quickly replaced by a Teflon-lined screw cap and the reaction was stirred at 30 °C for 18 h in a preheated metal block. The crude reaction mixture was taken in 20 mL EtOAc and washed with brine (2 x 10 mL), dried over Na_2SO_4 and concentrated under reduced pressure. Purification by silica gel column chromatography (Gradient: 6% to 12% EtOAc:hexanes) yields **S3** as a colorless oil (90.0 mg, 79% yield). In a 10 mL round bottom flask, enol ether **S3** (45 mg, 0.12 mmol) was dissolved in THF (2.0 mL) and 1M aq. HCl (2.0 mL). After completion (monitored by TLC) of the reaction, the aqueous layer was extracted with EtOAc (4 x 6 mL), dried over anhydrous Na_2SO_4, filtered and concentrated under reduced pressure to obtain the aldehyde **32** as colorless oil (39 mg, 94% yield).

IR (neat): 2976 (w), 2931 (w), 2876 (w), 1721 (m), 1692 (s), 1479 (w), 1392 (s), 1364 (s), 1324 (w), 1166 (m), 1142 (s), 1122 (m), 968 (w), 879 (m), 847 (m), 772 (m), 550 (w) cm^{-1}; **^{1}H NMR (400 MHz, $CDCl_3$):** δ 9.80 (s, 1H), 3.44 – 3.20 (m, 4H), 2.65 – 2.52 (m, 2H), 1.84 (t, *J* = 7.5 Hz, 2H), 1.45 (s, 9H), 1.23 (s, 12H), 1.19 – 1.06 (m, 2H); **^{13}C NMR (101 MHz, $CDCl_3$, *mixture of rotamers*):** δ 202.2, 202.1, 154.8, 154.7, 83.5, 79.3, 58.0, 57.7, 51.3, 51.2, 44.5, 44.2, 41.6, 40.9, 38.2, 37.4, 28.6, 25.1, 24.9, 24.9, 24.7; **HRMS (APCI+):** Calculated for $C_{18}H_{33}O_5NB$ $[M+H]^+$: 354.2446, Found: 354.2447.

6. Optimization table for vinyl triflate:

Table S1:

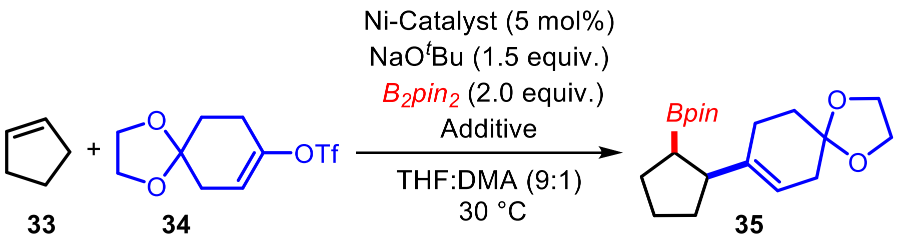

Entry	Ni-Catalyst	Additive	Yield (%)[a]
1	$Ni(DME)Cl_2$	None	60
2	$Ni(DME)Cl_2$	NaBr (30 mol%)	64
3	$Ni(DME)Br_2$	None	77
4	$Ni(DME)Br_2$	NaCl (60 mol%)	79
5	$Ni(DME)Br_2$	NaBr (60 mol%)	21
6	$Ni(DME)Br_2$	NaOTf (1 equiv.)	87
7	$Ni(DME)Br_2$	$NaBF_4$ (1 equiv.)	91 (70)[b]
8	$Ni(DME)Br_2$	$NaSbF_6$ (1 equiv.)	19
9	$Ni(DME)Br_2$	$NaPF_6$ (1 equiv.)	52

[a]Yield determined via GC analysis with a calibrated internal standard (dodecane). [b]Isolated yield.

7. General Procedure for Formal Alkylboration with Vinyl Triflates (General Procedure B):

In an N_2-filled glovebox, to an oven-dried 13 x 100 mm screw-capped vial was added bis(pinacolato)diboron (152 mg, 0.600 mmol, 2.00 equiv.), $NaBF_4$ (33 mg, 0.30 mmol, 1.0 equiv.) and NaO^{t}Bu (43 mg, 0.45 mmol, 1.5 equiv.). In a separate oven-dried 2-dram vial, $Ni(DME)Br_2$ (11.6 mg, 0.0375 mmol) was added. Both vials were sealed with septa and removed from the glovebox. THF (2.7 mL) was added to the reaction vial, followed by alkene (0.45 mmol, 1.5 equiv.) and vinyl triflate (0.3 mmol, 1.0 equiv.). DMA (0.75 mL) was then added to the vial containing $Ni(DME)Br_2$ (0.05 M in $Ni(DME)Br_2$) to prepare the catalyst solution. The catalyst solution (0.3 mL) was then added to the reaction vial (5 mol% catalyst loading). The septum was then quickly replaced by a Teflon-lined screw cap and the reaction was stirred at 30 °C for 18 h in a preheated metal block. The reaction was quenched upon the addition of 1 M HCl (5.0 mL), and the mixture

was extracted with EtOAc (3 x 5.0 mL). The organic layer was washed with 1 M KOH (2 x 5.0 mL), dried over Na_2SO_4, gravity filtered, and concentrated. 1,3,5-Trimethylbenzene was added as an internal standard and a small aliquot was analyzed via ^1H NMR. Purified by silica-gel column chromatography. In an oven dried 10-mL round-bottom flask, alkene was taken in MeOH (3.0 mL) or EtOAc (3.0 mL) under positive pressure of N_2. Pd-C (5 mol%) was added and hydrogen gas was bubbled through the solution for 5 min. The heterogeneous mixture was stirred at room temperature until TLC shows complete consumption of starting alkene. The reaction contents were filtered over celite and washed with EtOAc. The crude reaction mixture was purified by silica-gel column chromatography.

8. Optimization of Reduction Conditions for 1,2-Alkenylboration Product 35:

Table S2:

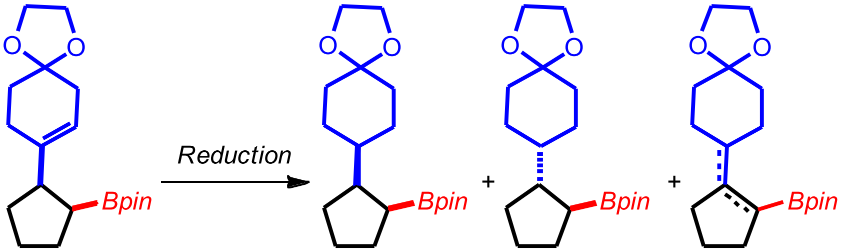

35 **36** **37** **38**

Entry	Reduction Conditions	Yield[a] (a:b:c)[b]
1	Pd-C, H_2 (1 atm) EtOAc, 25 °C	95% (1:1.6:0)
2	Crabtree's catalyst H_2 (1 atm), CH_2Cl_2, 0 °C	84% (5:0:1)
3	$Mn(dpm)_3$, $PhSiH_2(O^iPr)$ TBHP, Hexane, 25 °C	66%[c] (1:0:0)

[a]Yield determined by ^1H NMR analysis of the unpurified reaction mixture with an internal standard. [b]Ratios determined by GC anlysis. [c]Isolated yield.

9. Characterization Data for Vinyl Triflate Derived Products:

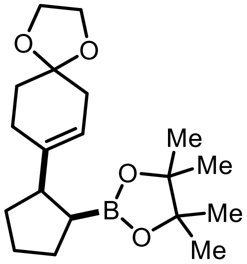

2-(2-(1,4-dioxaspiro[4.5]dec-7-en-8-yl)cyclopentyl)-4,4,5,5-tetramethyl-1,3,2-dioxaborolane (35): The title compound was prepared according to General Procedure B without hydrogenation. After the alkenylboration, the reaction mixture was taken in 20 mL EtOAc and washed with brine. Purification by silica gel column chromatography (Gradient: hexanes to 6% Et_2O:hexanes) yields **35** as white solid.

Average over 2 runs: 70% isolated yield, >20:1 d.r.

m.p. 52-54 ºC; **IR (neat):** 2979 (m), 2872 (m), 1372 (m), 1143 (m), 904 (s), 724 (s) cm^{-1}; **^1H NMR (400 MHz, $CDCl_3$):** δ 5.32 – 5.30 (m, 1H), 3.96 (s, 4H), 2.61 – 2.55 (m, 1H), 2.34 – 2.28 (m, 1H), 2.23 – 2.13 (m, 3H), 1.82 – 1.64 (m, 7H), 1.64 – 1.48 (m, 2H), 1.19 (s, 12H); **^{13}C NMR (126 MHz, $CDCl_3$):** δ 140.5, 116.3, 108.4, 82.8, 64.4, 64.4, 48.8, 35.7, 31.4, 30.4, 28.0, 27.5, 25.2, 25.2, 25.2, 25.1; **HRMS (ESI+):** Calculated for $C_{19}H_{32}O_4B$ [M+H]+: 335.2392, Found: 335.2391.

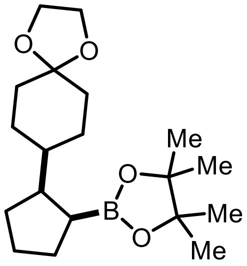

2-(2-(1,4-Dioxaspiro[4.5]decan-8-yl)cyclopentyl)-4,4,5,5-tetramethyl-1,3,2-dioxaborolane (36): In a 2-dram vial, alkenyl Bpin (33.4 mg, 0.100 mmol, 1.00 equiv.) was taken in 0.4 mL hexane under positive pressure of N_2. *Tert*-Butyl hydroperoxide (5.5 M in nonane, 36 µL, 0.20 mmol, 2.0 equiv.) and $PhSiH_2(O^iPr)$ (36 µL, 0.20 mmol, 2.0 equiv.) were added via micro syringe under inert atmosphere. The solution was degassed by bubbling N_2 through it for 10 min. $Mn(dpm)_3$ (2.4 mg, 4.0 mol%) was added as solid and the mixture was degassed for 30 seconds. After consumption of starting alkene, the crude reaction mixture was purified by silica-gel column chromatography (2% to 8% Et_2O:hexane) to obtain **36** as colorless oil (22 mg, 66% yield, >20:1 d.r.).

IR (neat): 2975 (w), 2937 (m), 2865 (m), 1445 (w), 1379 (m), 1371 (m), 1315 (s), 1230 (m), 1143 (s), 1036 (s), 925 (w), 860 (m), cm^{-1}; **^1H NMR (400 MHz, $CDCl_3$):** δ 3.92 (s, 4H), 1.87 – 1.68 (m, 6H), 1.68 – 1.58 (m, 3H), 1.55 – 1.41 (m, 4H), 1.37 – 1.22 (m, 4H), 1.22 – 1.17 (m, 12H); **^{13}C NMR (101 MHz, $CDCl_3$):** δ 109.3, 82.7, 64.4, 64.3, 49.2, 40.9, 34.8, 34.7, 30.6, 30.3, 29.7, 28.2, 25.1, 25.0, 24.8; **HRMS (APCI+):** Calculated for $C_{19}H_{34}O_4B$ $[M+H]^+$: 337.2545, Found: 337.2547.

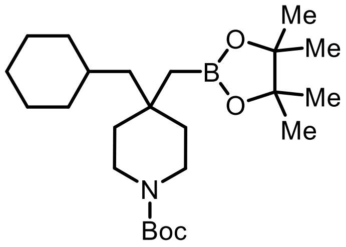

***Tert*-butyl 4-(cyclohexylmethyl)-4-((4,4,5,5-tetramethyl-1,3,2-dioxaborolan-2-yl)methyl) piperidine-1-carboxylate (41):** The title compound was prepared according to a modified version of General Procedure B using 1.0 equiv. of alkene and 1.5 equiv. of vinyl triflate. NMR yield of the unpurified reaction mixture was determined using 1,3,5-trimethylbenzene as an NMR standard. Hydrogenation was performed using Pd-C and MeOH as solvent. Purification for both the steps by silica gel column chromatography (gradient: 6% to 8% EtOAc:hexanes) yields **41** as a colorless oil.

Yield: 78% NMR yield (alkenylboration step), 68% isolated yield (over 2 steps).

IR (neat): 2976 (w), 2921 (m), 2850 (w), 1692 (s), 1448 (w), 1363 (s), 1320 (m), 1276 (w), 1245 (m), 1165 (s), 1143 (s), 971 (m), 867 (m), 847 (m) cm^{-1}; **^1H NMR (400 MHz, $CDCl_3$):** δ 3.48 – 3.42 (m, 2H), 3.30 – 3.23 (m, 2H), 1.69 – 1.61 (m, 5H), 1.47 – 1.38 (m, 13H), 1.26 – 1.24 (m, 4H), 1.22 (s, 12H), 1.19 – 1.06 (m, 2H), 1.00 – 0.94 (m, 2H), 0.91 (s, 2H); **^{13}C NMR (101 MHz, $CDCl_3$):** δ 155.2, 82.9, 79.0, 47.5, 40.0, 37.2, 36.1, 34.1, 33.2, 28.6, 26.7, 26.3, 25.0; **HRMS (ESI+):** Calculated for $C_{24}H_{45}O_4NB$ $[M+H]^+$: 422.3441, Found: 422.3436.

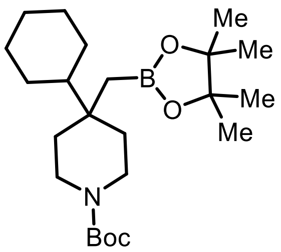

***Tert*-butyl 4-cyclohexyl-4-((4,4,5,5-tetramethyl-1,3,2-dioxaborolan-2-yl)methyl)piperidine-1-carboxylate (42):** The title compound was prepared according to a modified version of General Procedure B using 1.0 equiv. of alkene and 1.5 equiv. of vinyl triflate. NMR yield of the unpurified reaction mixture was determined using 1,3,5-trimethylbenzene as an NMR standard. Hydrogenation was performed using Pd-C and MeOH as solvent. Purification for both the steps by silica gel column chromatography (gradient: 8% to 10% EtOAc:hexanes) yields **42** as a colorless oil.

Average over 2 runs: 66% NMR yield (alkenylboration step), 61% isolated yield (over 2 steps), 4:1 (1,2 vs. 1,1 pdt).

IR (neat): 2975 (w), 2924 (m), 2852 (w), 1691 (s), 1379 (s), 1363 (m), 1317 (m), 1212 (m), 1163 (s), 1144 (s), 971 (w), 846 (w), 769 (w) cm^{-1};**^{1}H NMR (500 MHz, $CDCl_3$, *mixture of 1,2 and 1,1 pdt*):** δ 3.63 – 3.59 (m, 2H), 3.17 – 3.13 (m, 2H), 1.79 – 1.71 (m, 4H), 1.50 – 1.47 (m, 2H), 1.45 (bs, 11H), 1.29 – 1.26 (m, 2H), 1.23 (s, 12H), 1.19 – 1.09 (m, 3H), 1.02 – 0.96 (m, 2H), 0.87 (s, 2H); **^{13}C NMR (101 MHz, $CDCl_3$):** δ 155.3, 82.8, 79.1, 45.9, 40.4, 39.5, 36.1, 33.9, 28.6, 28.6, 27.3, 27.0, 26.9, 25.0; **HRMS (ESI+):** Calculated for $C_{23}H_{42}O_4NBNa$ $[M+Na]^+$: 430.3099, Found: 430.3098.

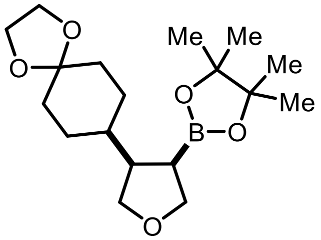

2-(4-(1,4-dioxaspiro[4.5]decan-8-yl)tetrahydrofuran-3-yl)-4,4,5,5-tetramethyl-1,3,2-dioxaborolane (43): The title compound was prepared according to a modified version of General Procedure B using 1.0 equiv. of alkene and 1.5 equiv. of vinyl triflate. After the alkenylboration, the reaction mixture was taken in 20 mL EtOAc and washed with brine. Hydrogenation was

performed using HAT conditions. Purification by silica gel column chromatography (gradient: 10% to 25% EtOAc: hexanes) yields **43** as a colorless oil.

Average over 2 runs: 69% isolated yield for alkenylboration step and 50% isolated yield for the reduction step.

IR (neat): 2976 (w), 2927 (m), 2866 (w), 1473 (w), 1378 (s), 1371 (s), 1322 (s), 1143 (s), 1098 (s), 924 (m), 858 (m) cm^{-1}; **^{1}H NMR (400 MHz, $CDCl_3$):** δ 3.91 (s, 4H), 3.89 – 3.86 (m, 3H), 3.51 – 3.47 (m, 1H), 2.17 – 2.08 (m, 1H), 1.85 – 1.79 (m, 1H), 1.77 – 1.69 (m, 3H), 1.57 – 1.42 (m, 3H), 1.40 – 1.26 (m, 3H), 1.23 (s, 12H). **^{13}C NMR (101 MHz, $CDCl_3$):** δ 108.81, 83.42, 71.71, 70.72, 64.36, 64.31, 48.08, 38.49, 34.60, 34.49, 30.15, 29.12, 25.01, 24.84. **HRMS (ESI+):** Calculated for $C_{18}H_{32}O_5B$ $[M+H]^+$: 339.2337 Found: 339.2339.

Diastereoselective Hydrogenation:

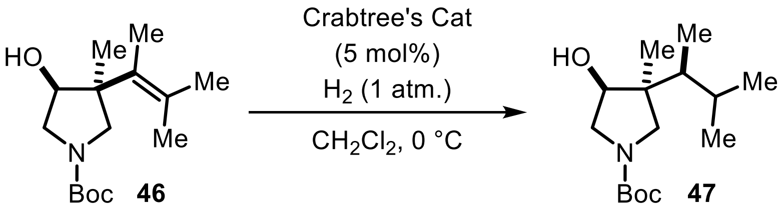

***Tert*-Butyl 4-hydroxy-3-methyl-3-(3-methylbutan-2-yl)pyrrolidine-1-carboxylate (47):** In an oven-dried 13 x 100 mm screw-capped vial, alkene **46** (36.0 mg, 0.13 mmol, 1.0 equiv.) was taken in dry CH_2Cl_2 (1 mL) under positive pressure of N_2. The solution was cooled to 0 ºC (ice bath) and hydrogen gas was bubbled into the solution for 5 min. Crabtree's catalyst (5 mol% in 0.5 mL CH_2Cl_2) was added hourly (1 mol% as solution in CH_2Cl_2 per hour). Purification by silica gel column chromatography (Gradient: 15% to 25% EtOAc:hexanes) yields **47** as a colorless oil (30.0 mg, 85%, 4:1 dr).

IR (neat): 3390 (w), 3357 (w), 2959 (m), 2932 (m), 2878 (w), 2868 (w), 1664 (s), 1474 (w), 1419 (s), 1364 (m), 1207 (w), 1117 (s), 1087 (s), 1015 (w), 888 (m), 769 (m), 571 (w) cm^{-1}; **^{1}H NMR (400 MHz, $CDCl_3$, *mixture of diastereomers*):** δ 3.96 (s, 1H, *major*), 3.81 (s, 1H, *minor*), 3.64 – 3.56 (m, 1H), 3.42 – 3.18 (m, 2H), 3.12 – 3.07 (m, 1H), 1.89 – 1.76 (m, 3H), 1.44 (s, 9H), 1.24 (s, 1H), 0.94 (d, J = 6.7 Hz, 3H), 0.88 – 0.84 (m, 6H), 0.76 (dd, J = 7.2, 2.4 Hz, 3H, *major*); **^{13}C NMR (101 MHz, $CDCl_3$, *mixture of diastereomers and rotamers*):** δ 155.3, 155.1, 79.4, 79.4, 75.9,

56.2, 55.6, 54.6, 54.4, 50.4, 49.7, 39.0, 38.9, 29.8, 28.7, 28.7, 28.1, 28.1, 24.2, 24.2, 18.8, 18.8, 17.6, 9.8; **HRMS (ESI+):** Calculated for $C_{15}H_{29}O_3NNa$ [M+Na]$^+$: 294.2040, Found: 294.2042.

Note: Using Pd-C catalyst in MeOH opposite major diastereomer of the product was obtained in 97% yield and 2:1 diastereomeric ratio. Crystal obtained from the sample of Pd-C reduction shows the major diastereomer as in the case of Crabtree's catalyst.

10. Synthesis of *Tamoxilog* Analog:

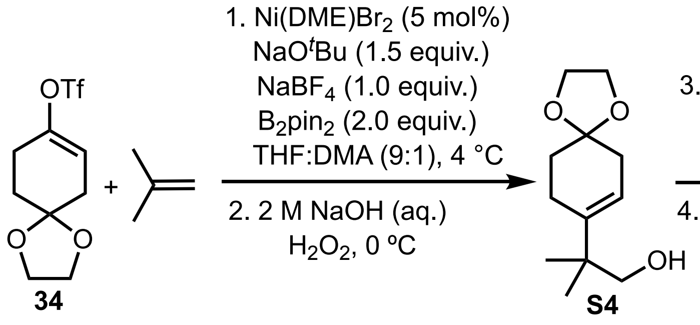

4-(1-Hydroxy-2-methylpropan-2-yl)cyclohexan-1-one (S5): Alkenylboration was performed following the General Procedure B using 1.0 equiv. of triflate and 10 equiv. of 2-methylprop-1-ene (solution in THF) at 4 ºC on a 0.8 mmol scale. The crude reaction mixture was taken in 5 mL THF, cooled to 0 ºC (ice bath) followed by the addition of 2 M NaOH (aq.) and H_2O_2. After 1h, 10 mL water was added, and the aqueous layer was extracted with EtOAc (3 X 10 mL). Organic layer was washed with brine (10 mL), dried over Na_2SO_4 and purified by silica-gel column chromatography (Gradient: 20% to 50% EtOAc:hexanes) to obtain **S4** as a clear oil (52% isolated yield). In an oven-dried 13 x 100 mm screw-capped vial, alkene **S4** (1.0 equiv.) was taken in dry CH_2Cl_2 (0.1 M) under positive pressure of N_2. The solution was cooled to 0 ºC (ice bath) and hydrogen gas was bubbled into the solution for 5 min. Crabtree's catalyst (3.0 mol% in 0.3 mL CH_2Cl_2) was added hourly (1 mol% as solution in CH_2Cl_2 per hour). After completion of the reduction, solvent was removed, and the crude reaction mixture was taken in 3 mL THF. The solution was cooled to 0 ºC (ice bath) followed by the addition of 3 mL 3 M HCl (aq.) and stirring was continued overnight. The aqueous layer was extracted with EtOAc (3 X 5 mL), dried over Na_2SO_4 and purification by silica-gel column chromatography (Gradient: 20% to 50% EtOAc:hexanes) afforded **S5** (95% yield) as a colorless oil. **IR (neat):** 3429 (w), 2956 (s), 1712 (s) cm^{-1}; **HRMS (ESI+):** Calculated for $C_{10}H_{19}O_2$ [M+H]$^+$: 171.1385, Found: 171.1380; **^{1}H NMR (500 MHz, CDCl$_3$):** δ 3.42 (s, 2H), 2.41 – 2.29 (m, 4H), 2.08 – 2.04 (m, 2H), 1.82 – 1.76 (m, 1H),

1.57 (s, 1H), 1.52 – 1.44 (m, 2H), 0.88 (s, 6H); **^{13}C NMR (126 MHz, $CDCl_3$):** δ 212.6, 70.7, 41.6, 41.4, 37.3, 27.4, 22.0.

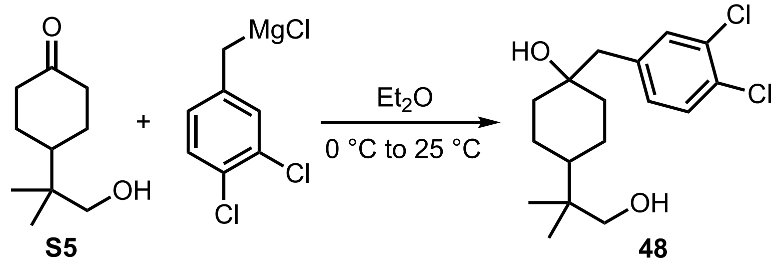

1-(3,4-Dichlorobenzyl)-4-(1-hydroxy-2-methylpropan-2-yl)cyclohexan-1-ol (48): In an oven-dried 13 x 100 mm screw-capped vial, keto-alcohol **S5** (1.0 equiv.) was taken in dry Et_2O (0.1 M) under positive pressure of N_2. The solution was cooled to 0 ºC (ice bath) and freshly prepared (3,4-dichlorobenzyl)magnesium chloride (0.3 M in Et_2O, 2.5 equiv.) was added dropwise. The reaction mixture was allowed to warm to 25 ºC and stirred for 3 h. After complete consumption of **S5** (TLC), quenched with sat. NH_4Cl (aq.) solution and the aqueous layer was extracted with EtOAc (3 X 5 mL), dried over Na_2SO_4. Purification by silica-gel column chromatography (Gradient: 20% to 40% EtOAc:hexanes) afforded **48** (88% yield, combined yield of *cis* and *trans* isomers) as a white solid. Spectral data matches with the previously reported literature.[18]

***Cis*-48**: **^{1}H NMR (400 MHz, CD_3OD):** δ 7.37 – 7.35 (m, 2H), 7.10 (dd, *J* = 8.2, 2.0 Hz, 1H), 3.28 (s, 2H), 2.63 (s, 2H), 1.57 (d, *J* = 9.8 Hz, 2H), 1.49 (d, *J* = 9.5 Hz, 2H), 1.41 – 1.31 (m, 4H), 1.23 – 1.18 (m, 1H), 0.79 (s, 6H). **^{13}C NMR (126 MHz, CD_3OD):** δ 140.1, 133.6, 132.4, 131.7, 130.9, 130.7, 71.6, 70.7, 50.3, 43.8, 38.1, 38.0, 23.0, 22.3.

***Trans*-48**: **^{1}H NMR (400 MHz, CD_3OD):** δ 7.39 (s, 1H), 7.35 (d, *J* = 8.3 Hz, 1H), 7.13 (d, *J* = 8.4 Hz, 1H), 3.30 (s, 2H), 2.75 (s, 2H), 1.66-1.63 (m, 4H), 1.40 – 1.25 (m, 6H), 0.84 (s, 6H). **^{13}C NMR (126 MHz, CD_3OD):** δ 140.4, 133.7, 132.5, 131.6, 130.9, 130.7, 72.9, 70.8, 44.0, 42.4, 39.2, 37.9, 25.1, 22.5.

Comparison of ^{1}H NMR (CD_3OD) Spectroscopic Data for ***cis*-48**

Literature Data ^{1}H NMR (500 MHz, CD_3OD)	Our Data ^{1}H NMR (400 MHz, CD_3OD)
7.37 – 7.36 (m, 2H)	7.37 – 7.35 (m, 2H)
7.10 (dd, *J* = 8.2, 2.0 Hz, 1H)	7.10 (dd, *J* = 8.2, 2.0 Hz, 1H)
3.28 (s, 2H)	3.28 (s, 2H)
2.63 (s, 2H)	2.63 (s, 2H)
1.57 (br d, *J* = 10.5 Hz, 2H)	1.57 (d, *J* = 9.8 Hz, 2H)
1.48 (br d, *J* = 10.5 Hz, 2H)	1.49 (d, *J* = 9.5 Hz, 2H)
1.41 – 1.31 (m, 4H)	1.41 – 1.31 (m, 4H)
1.23 – 1.18 (m, 1H)	1.23 – 1.18 (m, 1H)
0.79 (s, 6H)	0.79 (s, 6H)

Comparison of ^{13}C NMR (126 MHz, CD_3OD) Spectroscopic Data for ***cis*-48**

Literature Data ^{13}C NMR (126 MHz, CD_3OD)	Our Data ^{13}C NMR (126 MHz, CD_3OD)
140.1	140.1
133.6	133.6
132.4	132.4
131.6	131.7
130.9	130.9
130.7	130.7
71.6	71.6
70.7	70.7
50.3	50.3
43.7	43.8
38.1	38.1
38.0	38.0
22.9	23.0
22.3	22.3

Comparison of ^{1}**H NMR (CD$_3$OD)** Spectroscopic Data for ***trans*-48**

Literature Data ^{1}**H NMR (500 MHz, CD$_3$OD)**	Our Data ^{1}**H NMR (400 MHz, CD$_3$OD)**
7.39 (d, *J* = 2.0 Hz, 1H)	7.39 (s, 1H)
7.35 (d, *J* = 8.5 Hz, 1H)	7.35 (d, *J* = 8.3 Hz, 1H)
7.13 (dd, *J* = 8.0, 2.0 Hz, 1H)	7.13 (d, *J* = 8.4 Hz, 1H)
3.31 (s, 2H)	3.30 (s, 2H)
2.75 (s, 2H)	2.75 (s, 2H)
1.66 (br d, *J* = 9.5 Hz, 4H)	1.66-1.63 (m, 4H)
1.38 – 1.22 (m, 5H)	1.40 – 1.25 (m, 6H)
	0.84 (s, 6H)

Comparison of ^{13}**C NMR (126 MHz, CD$_3$OD)** Spectroscopic Data for ***trans*-48**

Literature Data ^{13}**C NMR (126 MHz, CD$_3$OD)**	Our Data ^{13}**C NMR (126 MHz, CD$_3$OD)**
140.3	140.4
133.7	133.7
132.4	132.5
131.6	131.6
129.9	130.9
130.7	130.7
72.8	72.9
70.8	70.8
43.9	44.0
42.3	42.4
39.1	39.2
37.8	37.9
25.1	25.1
22.4	22.5

11. Single crystal X-ray diffraction analysis of 47:

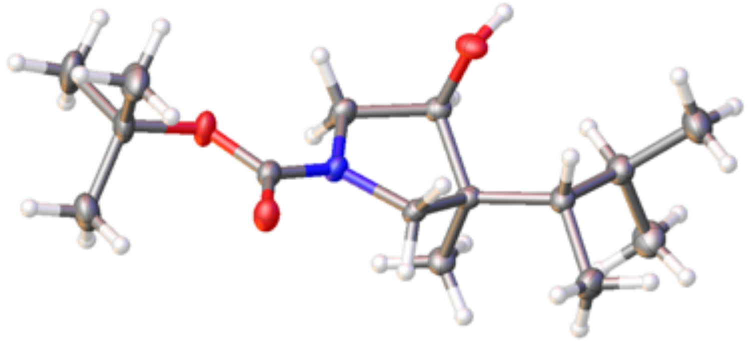

Table S3 Crystal data and structure refinement for 47.

Identification code	**47**	
Empirical formula	$C_{15}H_{29}NO_3$	
Formula weight	271.39	
Temperature	100(2) K	
Crystal system	monoclinic	
Space group	*P2₁/c*	
	a = 12.3834(11) Å	α = 90 °
	b = 10.9459(9) Å	β = 90.342(3) °
	c = 12.0235(9) Å	γ = 90 °
Volume	1629.7(2) Å^3	
Z	4	
Density (calculated)	1.106 g/cm^3	
Absorption coefficient	0.076 mm^{-1}	
F(000)	600.0	
Crystal size	0.140 × 0.110 × 0.030 mm^3	
Radiation	MoKα (λ = 0.71073)	
Theta range for data collection	4.966 to 52.81 °	
Index ranges	−15 ≤ h ≤ 15, −13 ≤ k ≤ 13, −15 ≤ l ≤ 13	
Reflections collected	27891	
Independent reflections	3333 [R_{int} = 0.0936, $R\sigma$ = 0.0458]	
Data/restraints/parameters	3333/ 0/ 183	

Goodness-of-fit on F^2	1.048
Final R indexes [I>g2σ (I)]	R_1 = 0.0512, ωR_2 = 0.1214
Final R indexes [all data]	R_1 = 0.0739, ωR_2 = 0.1394
Largest diff. peak/hole	0.49/-0.23 e Å^{-3}

11. Spectra:

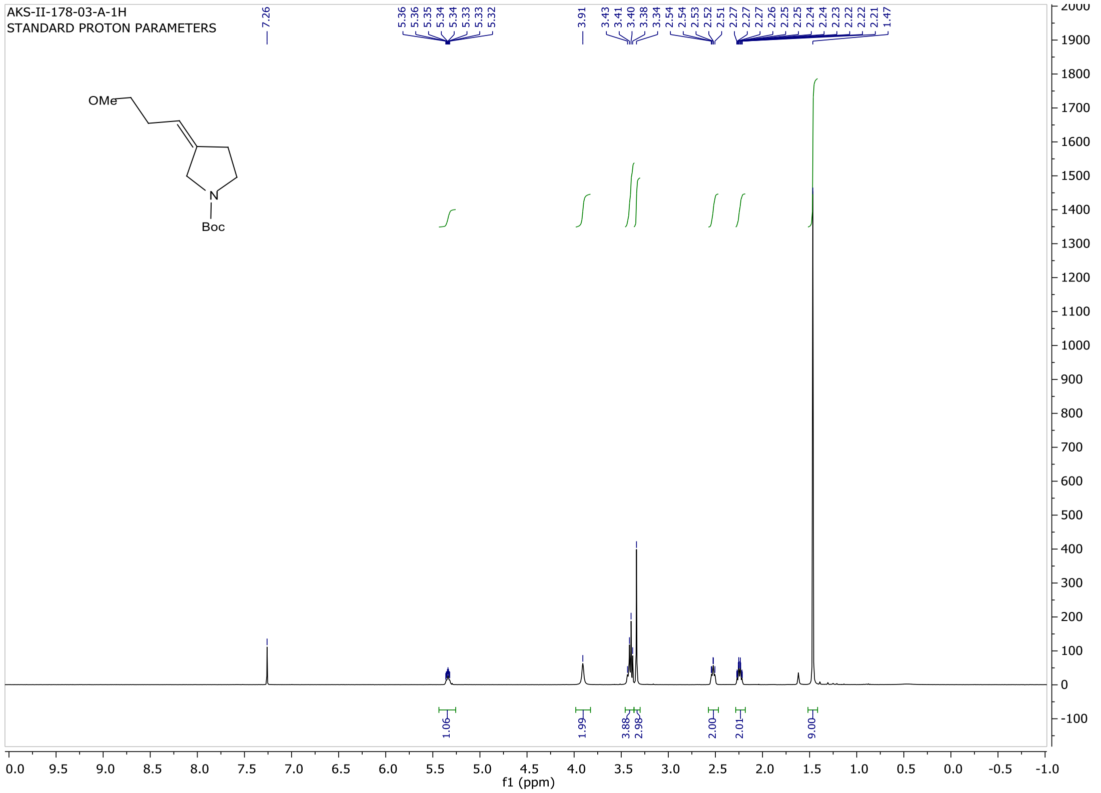
AKS-II-178-03-A-1H
STANDARD PROTON PARAMETERS
OMe
N
Boc
f1 (ppm)

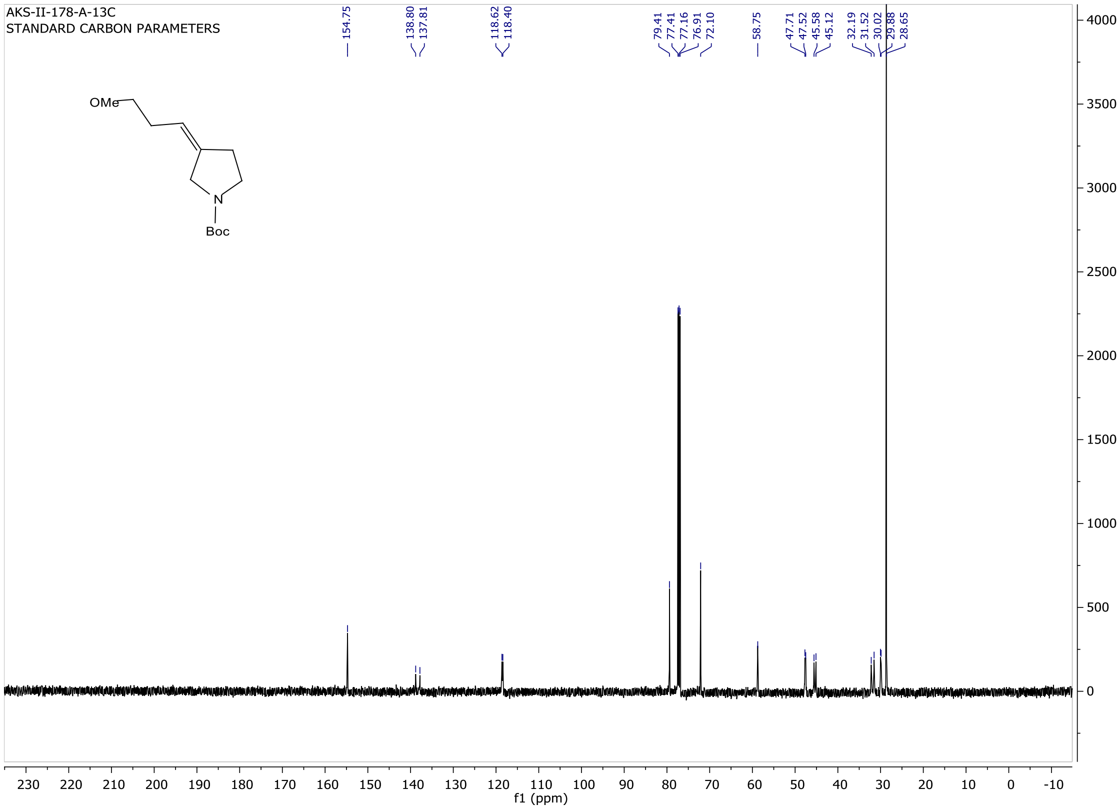
AKS-II-178-A-13C
STANDARD CARBON PARAMETERS
OMe
N
Boc
f1 (ppm)

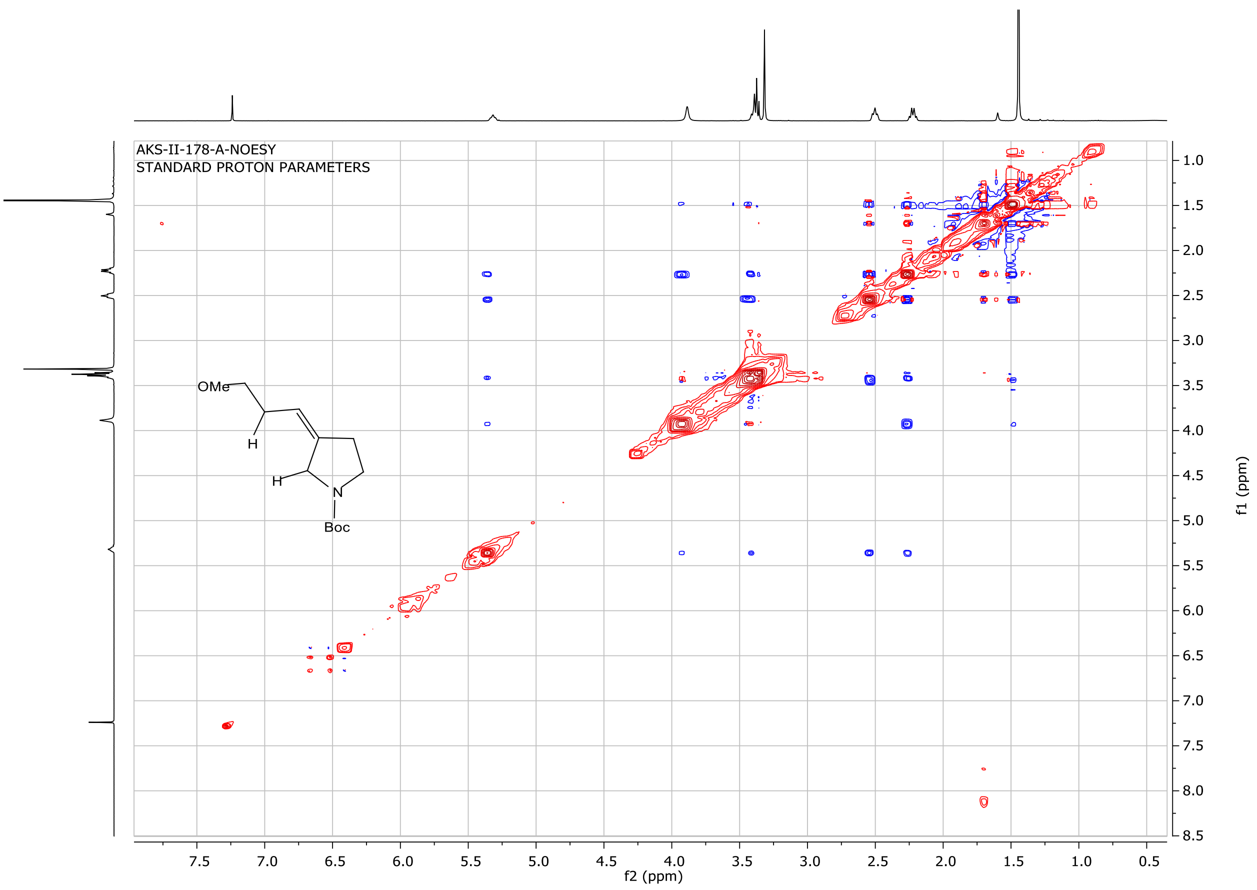
AKS-II-178-A-NOESY
STANDARD PROTON PARAMETERS
OMe
H
H
N
Boc
f2 (ppm)
f1 (ppm)

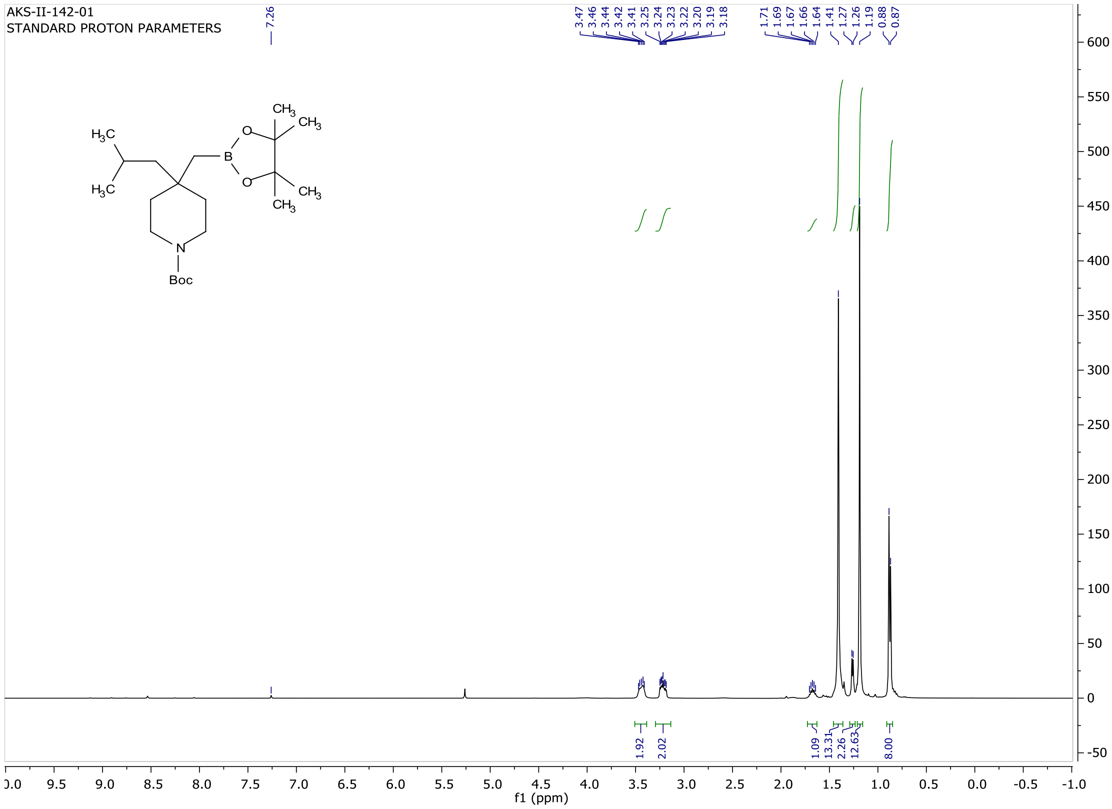

AKS-II-142-01
STANDARD PROTON PARAMETERS
Boc
f1 (ppm)

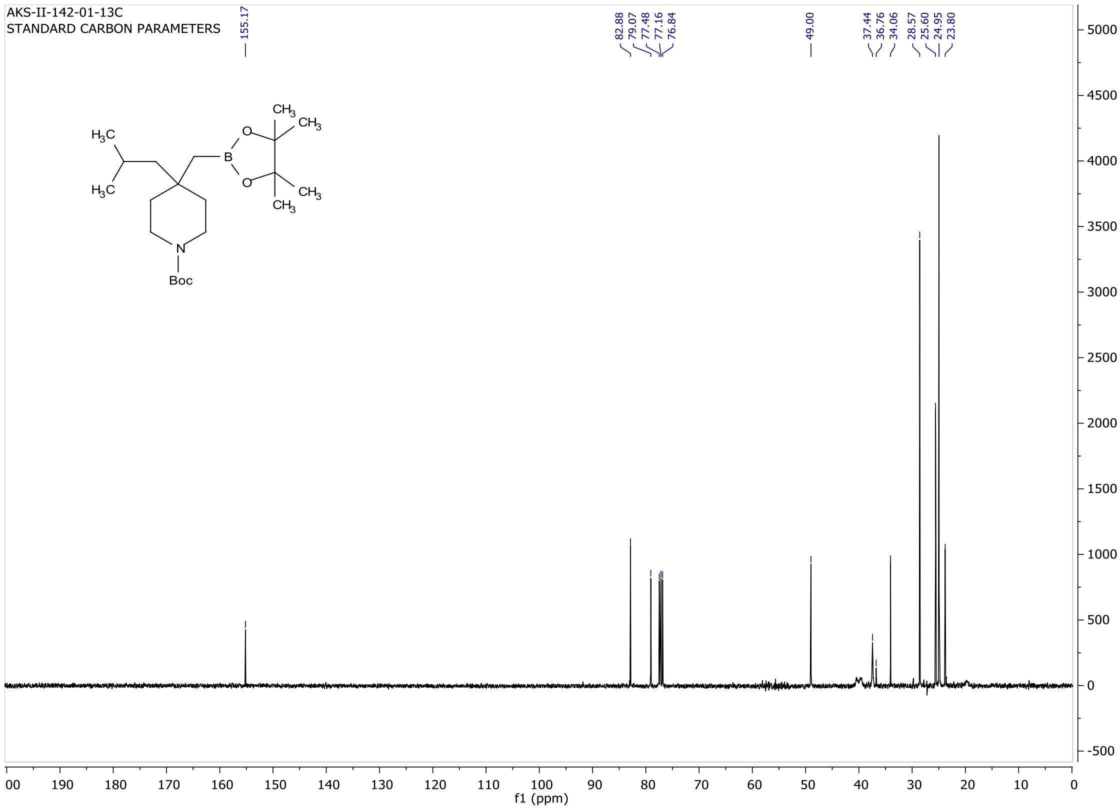

AKS-II-142-01-13C
STANDARD CARBON PARAMETERS
Boc
f1 (ppm)

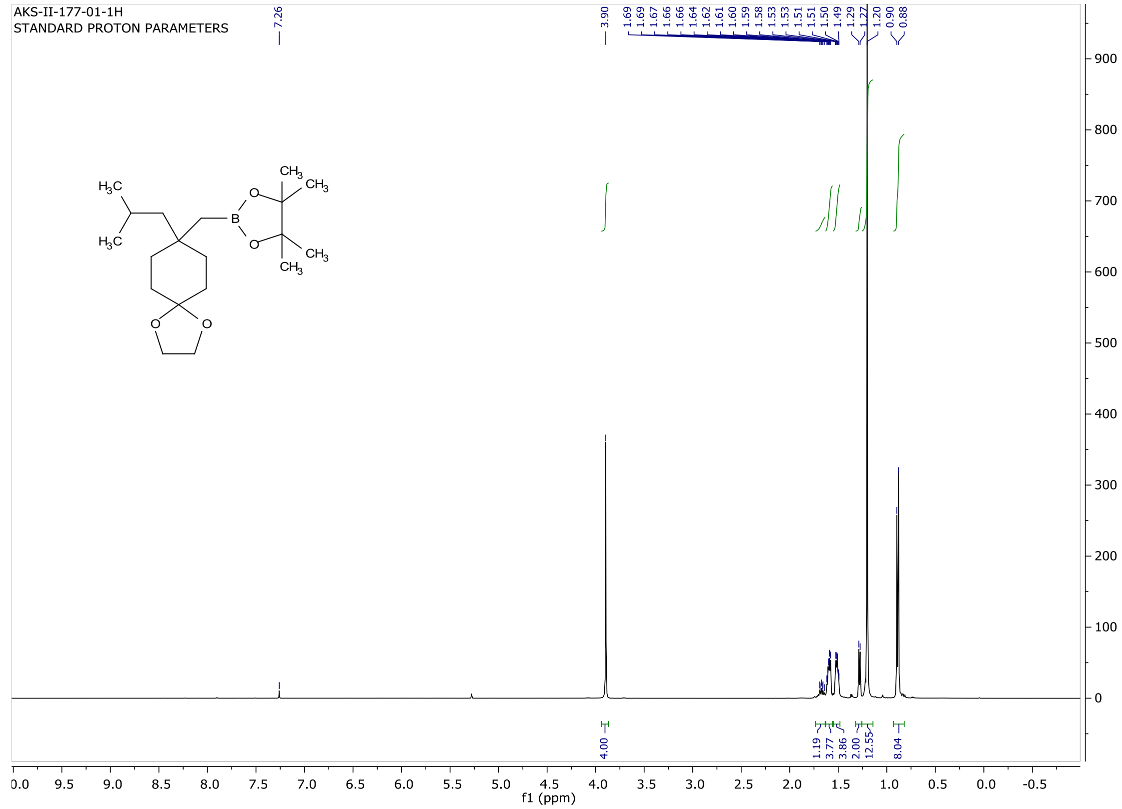
AKS-II-177-01-1H
STANDARD PROTON PARAMETERS
7.26
3.90
1.69 1.69 1.67 1.66 1.66 1.64 1.62 1.61 1.60 1.59 1.58 1.53 1.53 1.51 1.51 1.50 1.49 1.29 1.22 1.20 0.90 0.88
4.00
1.19
3.77
3.86
2.00
12.55
8.04
f1 (ppm)

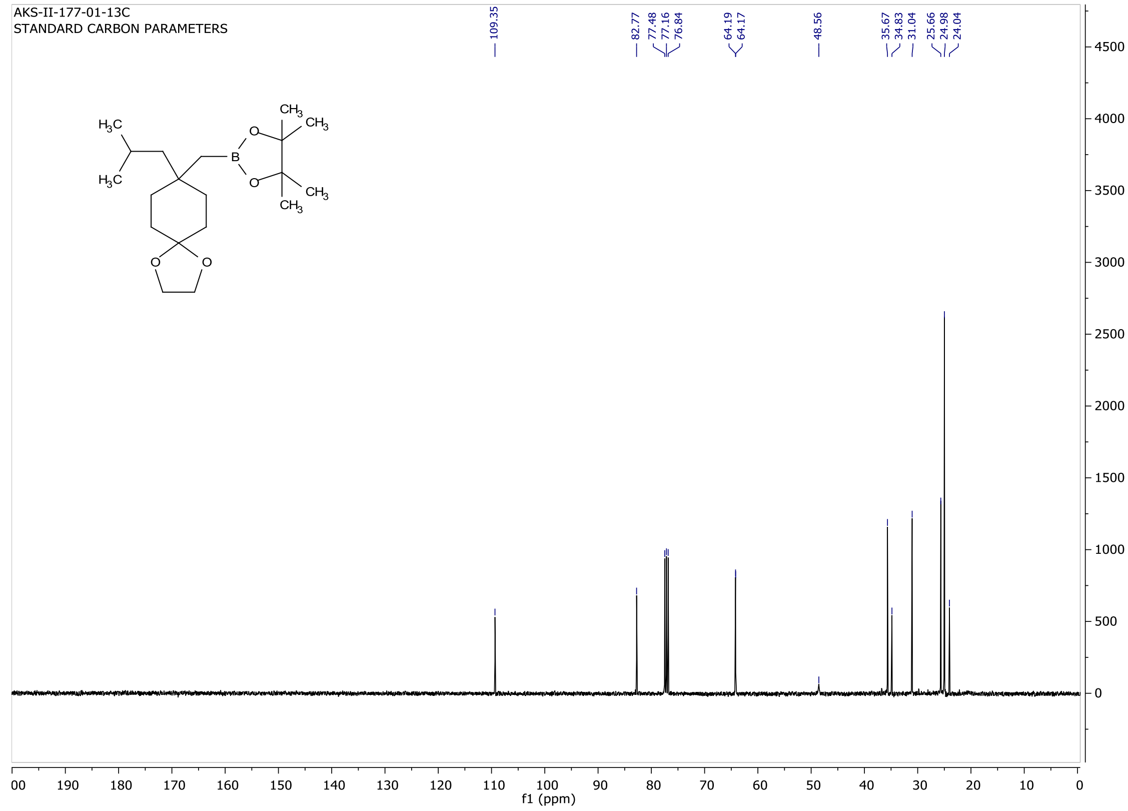
AKS-II-177-01-13C
STANDARD CARBON PARAMETERS
109.35
82.77 77.48 77.16 76.84
64.19 64.17
48.56
35.67 34.83 31.04 25.66 24.98 24.04
f1 (ppm)

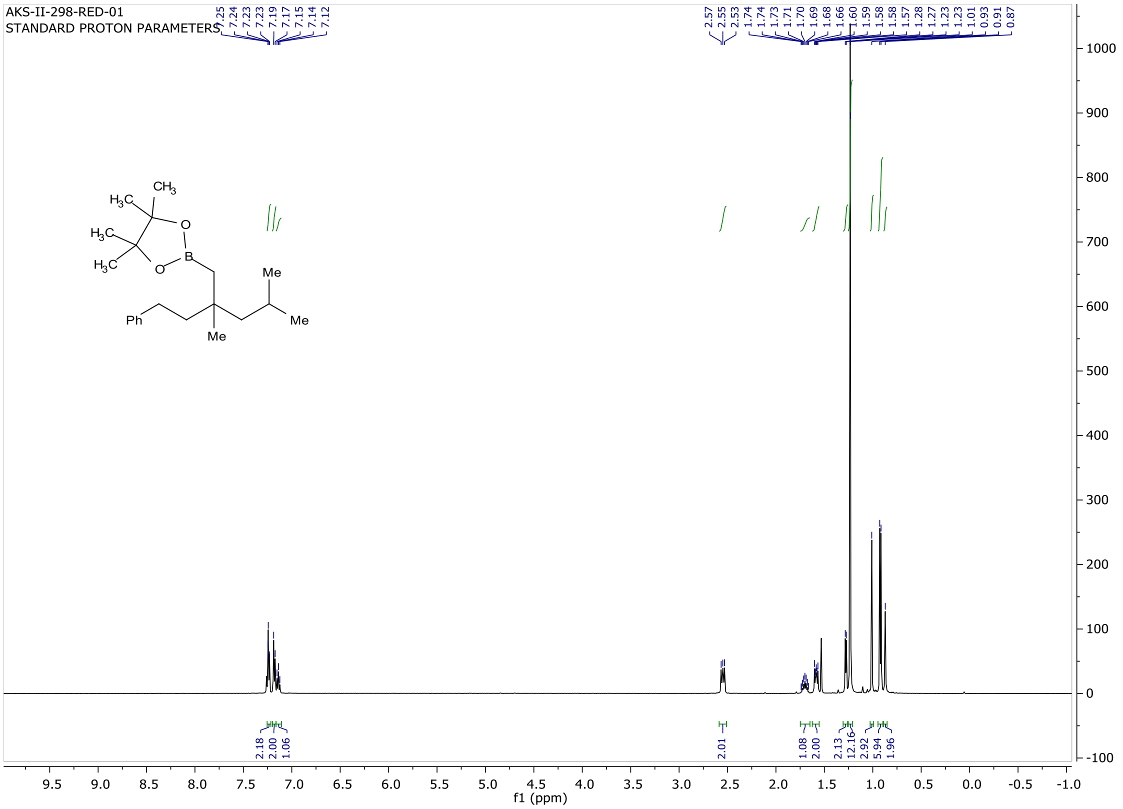
AKS-II-298-RED-01
STANDARD PROTON PARAMETERS
7.25 7.24 7.23 7.23 7.19 7.17 7.15 7.14 7.12
2.57 2.55 2.53 1.74 1.74 1.73 1.71 1.70 1.69 1.68 1.66 1.60 1.59 1.58 1.58 1.57 1.28 1.27 1.23 1.23 1.01 0.93 0.91 0.87
2.18 2.00 1.06 2.01 1.08 2.00 2.13 12.16 2.92 5.94 1.96
f1 (ppm)

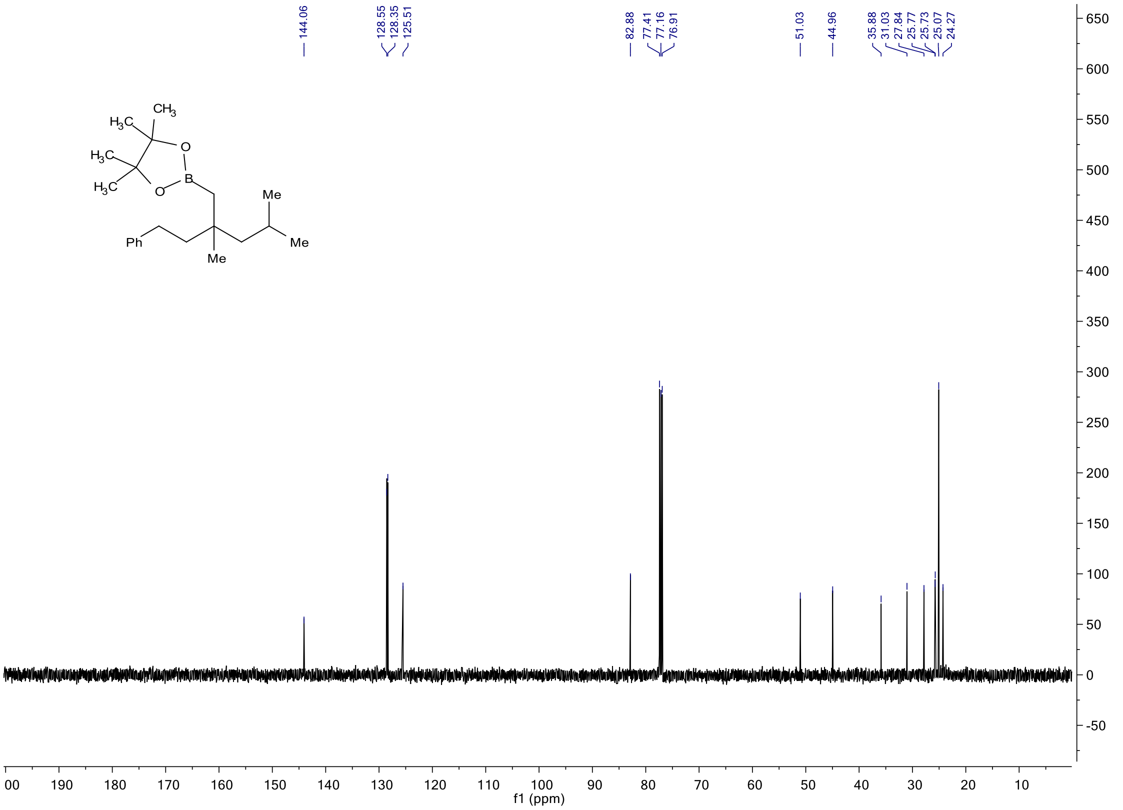
144.06 128.55 128.35 125.51 82.88 77.41 77.16 76.91 51.03 44.96 35.88 31.03 27.84 25.77 25.73 25.07 24.27
f1 (ppm)

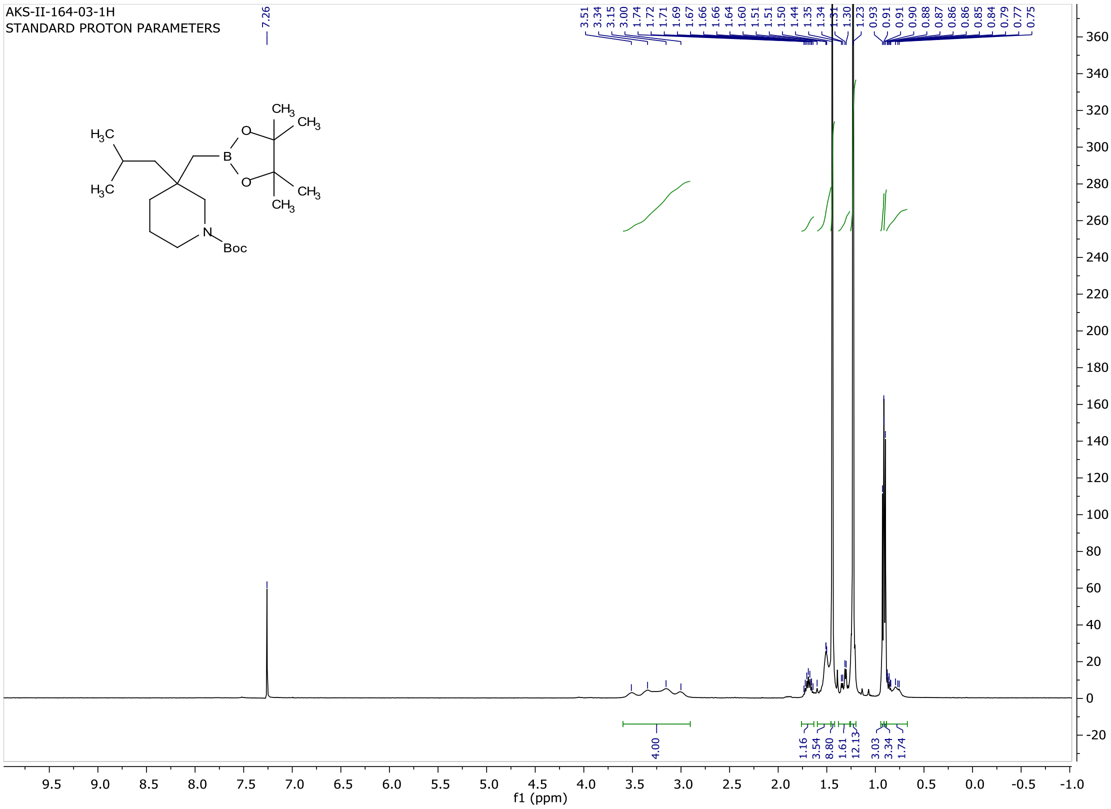
AKS-II-164-03-1H
STANDARD PROTON PARAMETERS
f1 (ppm)

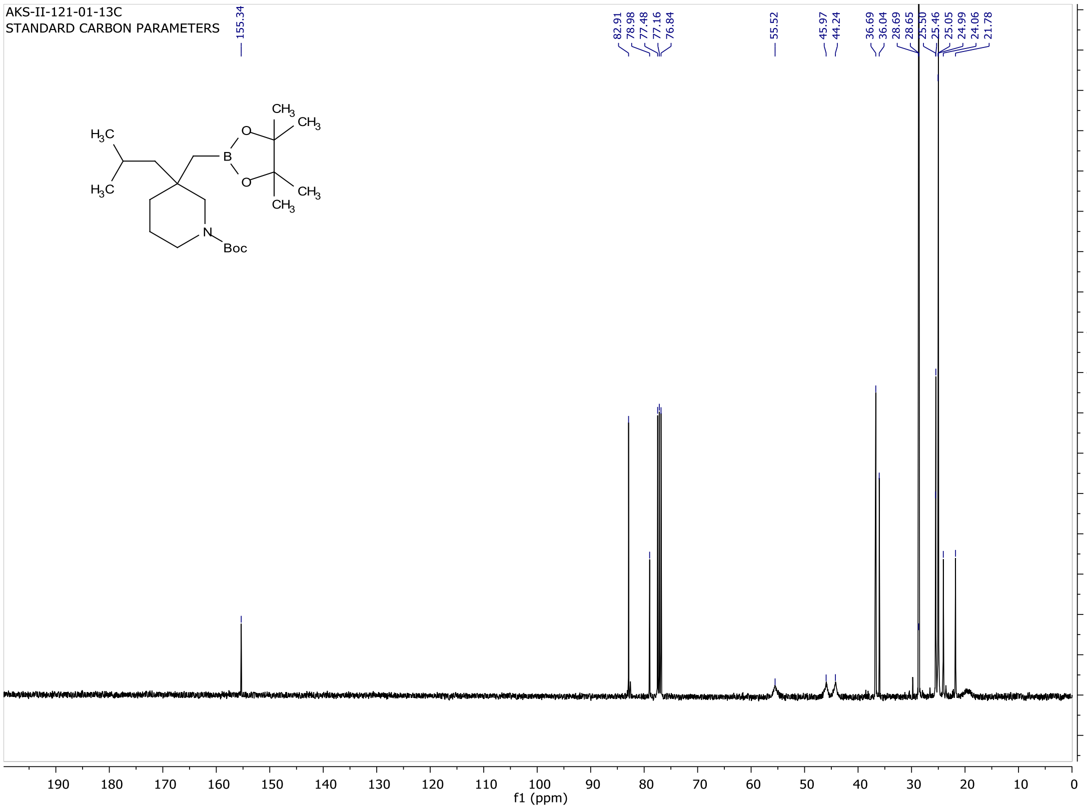
AKS-II-121-01-13C
STANDARD CARBON PARAMETERS
f1 (ppm)

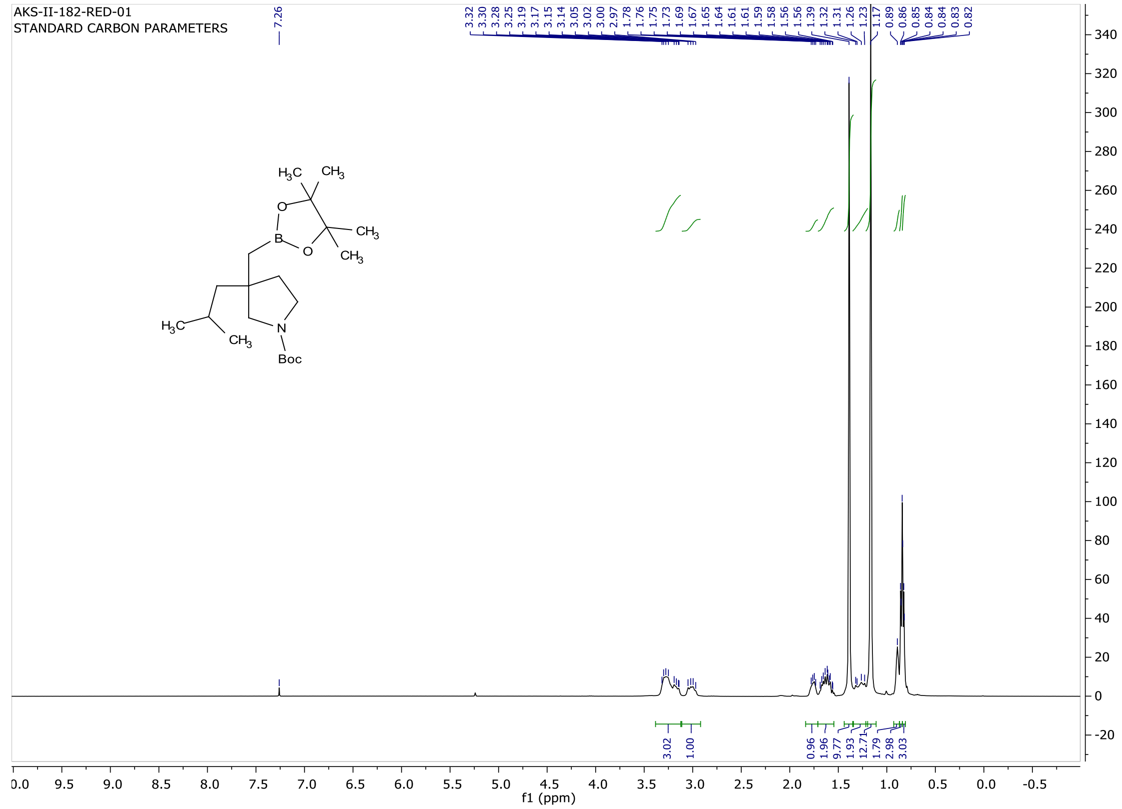
AKS-II-182-RED-01
STANDARD CARBON PARAMETERS
7.26
3.32 3.30 3.28 3.25 3.19 3.17 3.15 3.14 3.05 3.02 3.00 2.97 1.78 1.76 1.75 1.73 1.69 1.67 1.65 1.64 1.61 1.61 1.59 1.58 1.56 1.56 1.39 1.32 1.31 1.26 1.23 1.17 0.89 0.86 0.85 0.84 0.84 0.83 0.82
3.02 1.00 0.96 1.96 9.77 1.93 12.71 1.79 2.98 3.03
Boc
f1 (ppm)

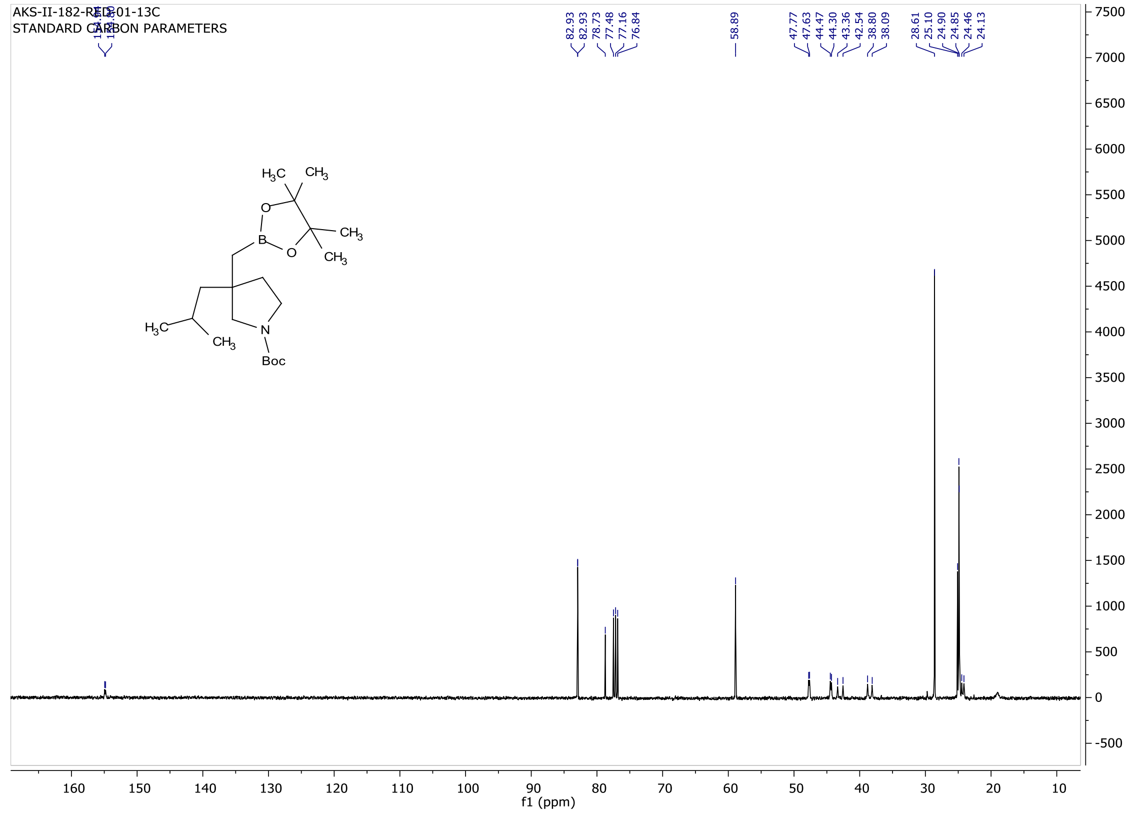
AKS-II-182-RED-01-13C
STANDARD CARBON PARAMETERS
82.93 82.93 78.73 77.48 77.16 76.84
58.89
47.77 47.63 44.47 44.30 43.36 42.54 38.80 38.09
28.61 25.10 24.90 24.85 24.46 24.13
Boc
f1 (ppm)

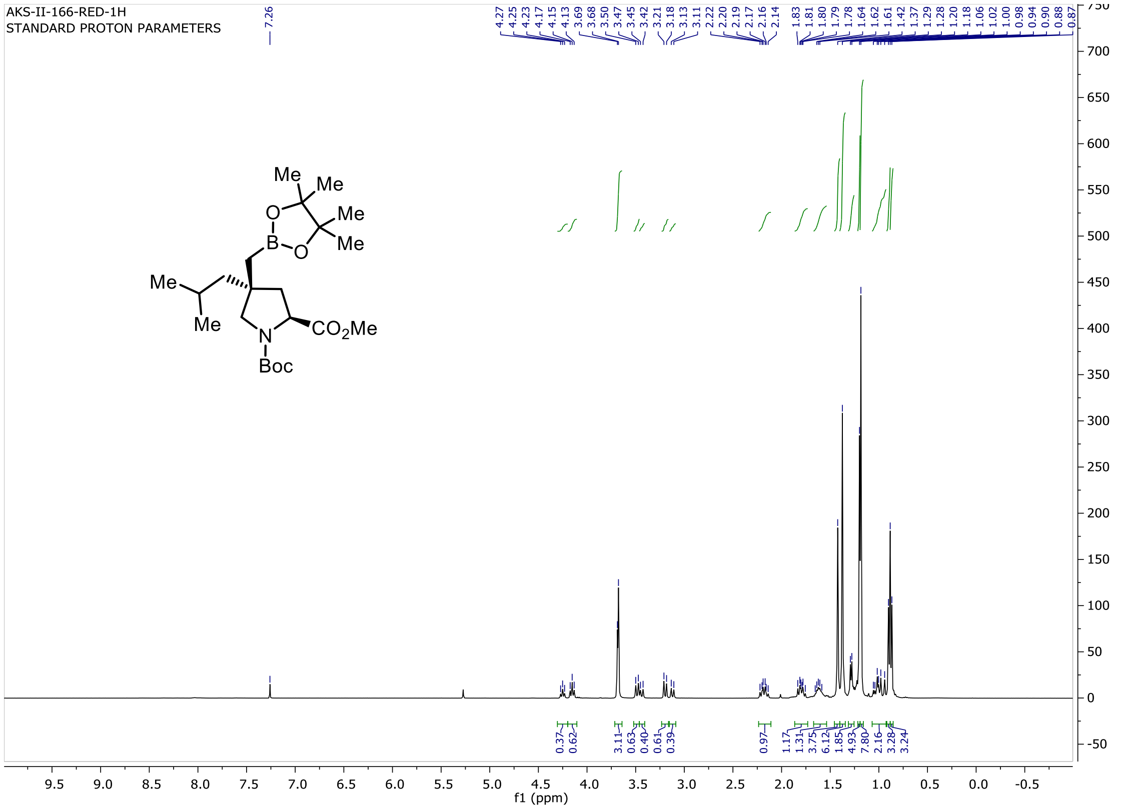

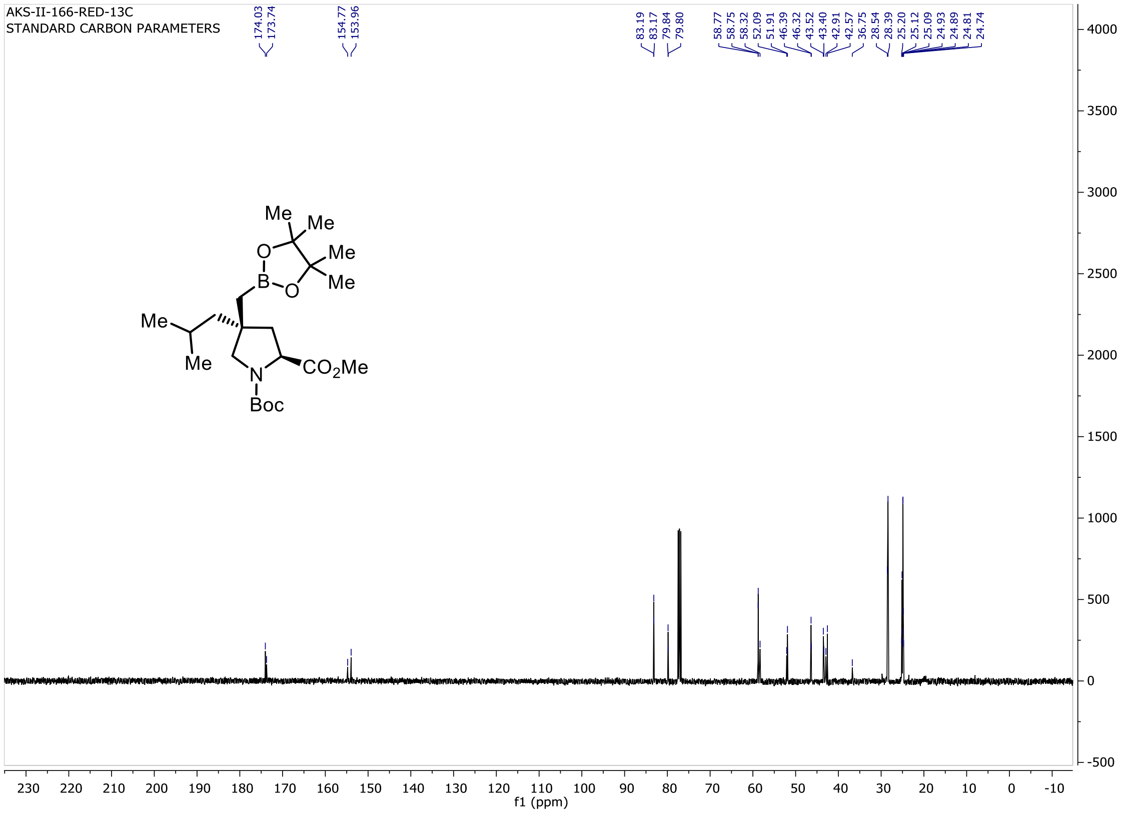

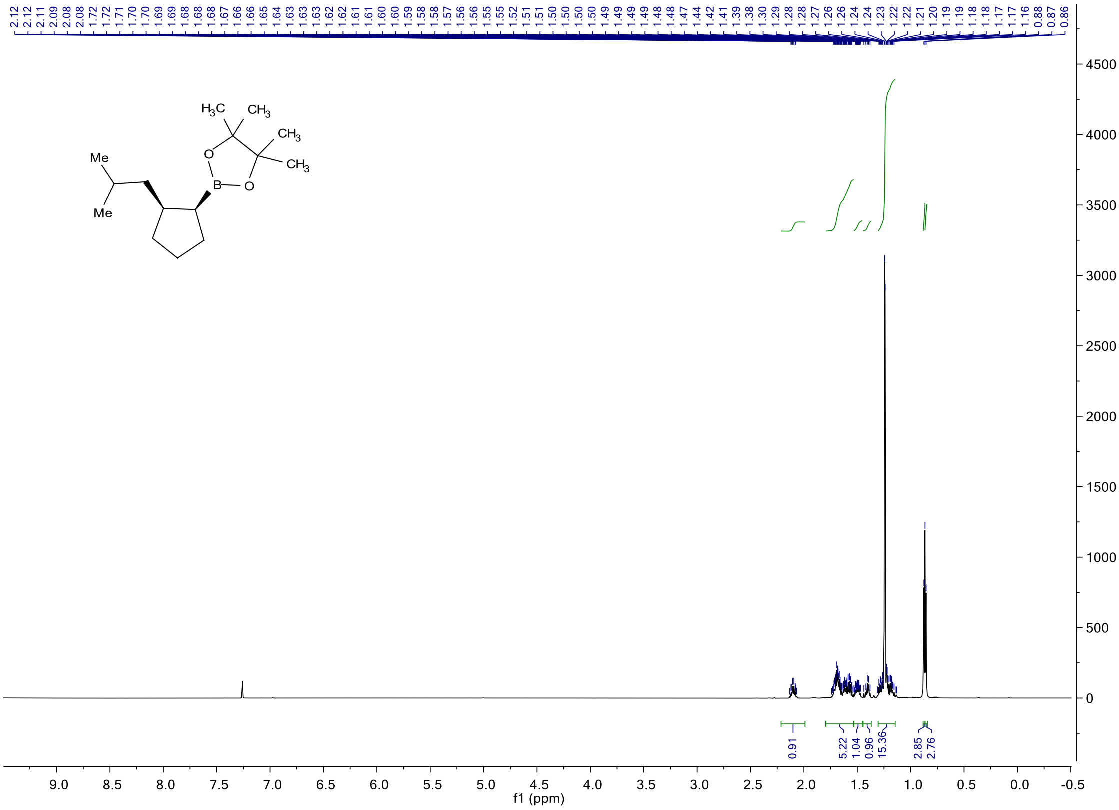

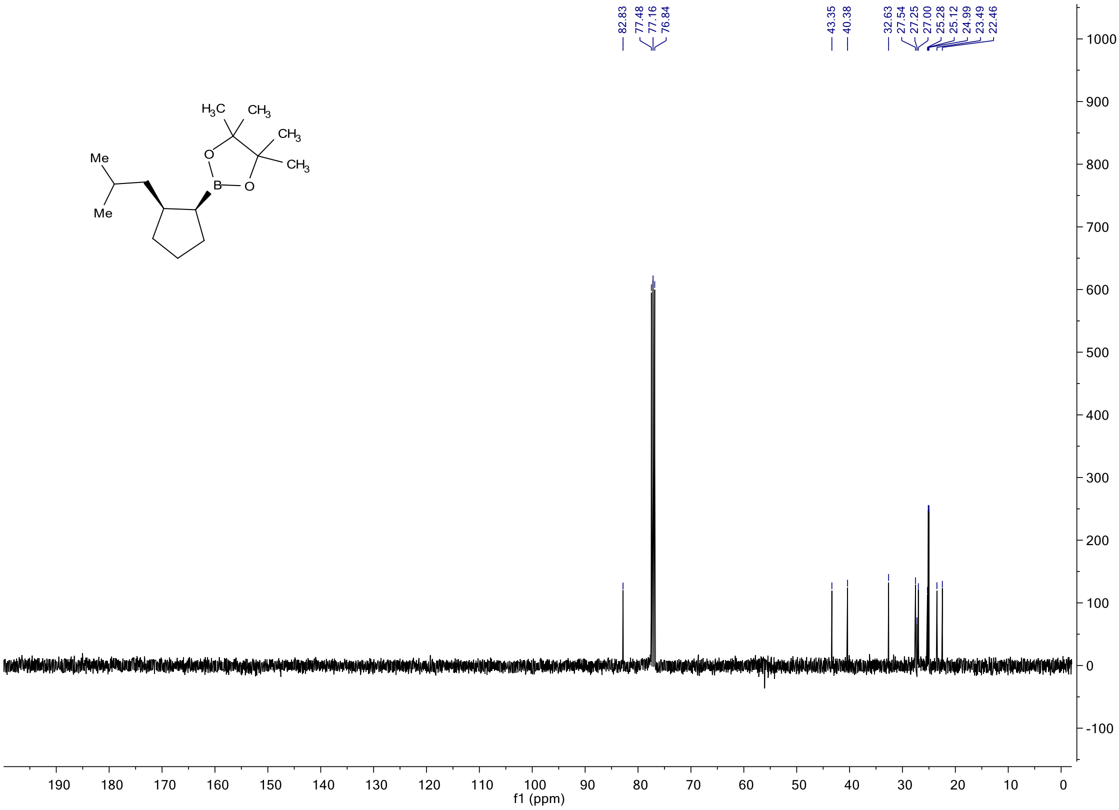

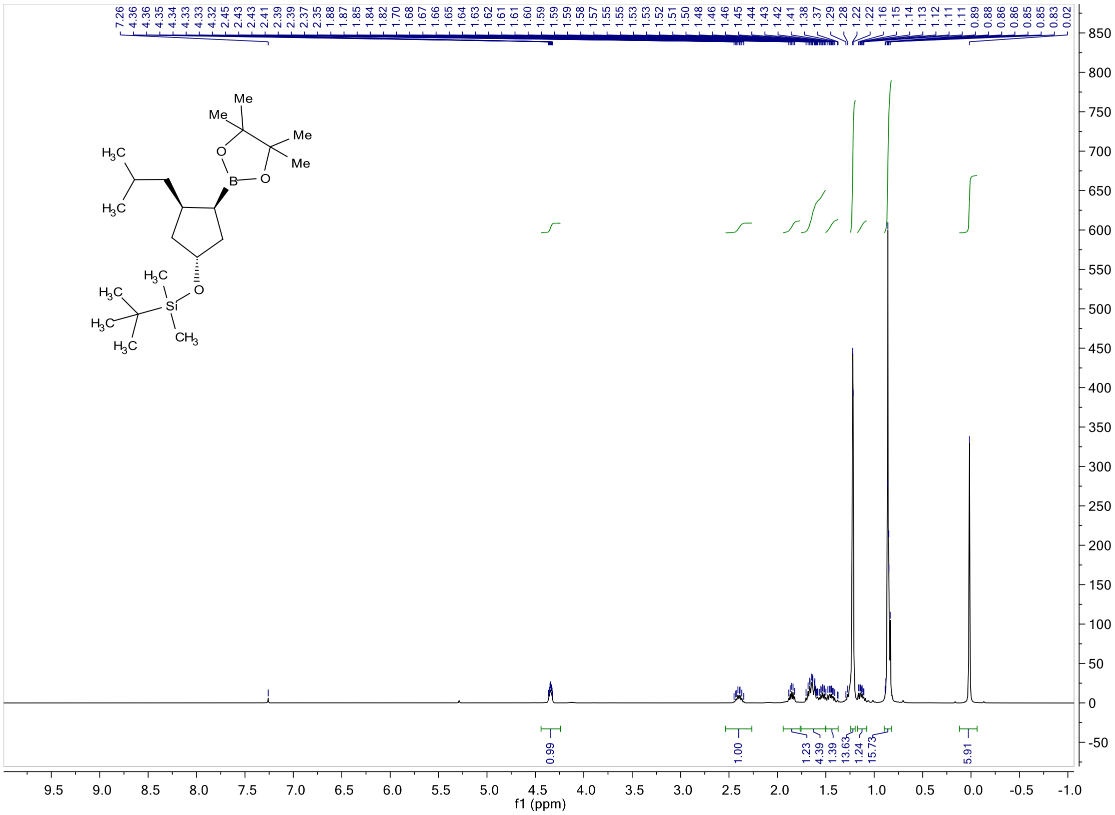

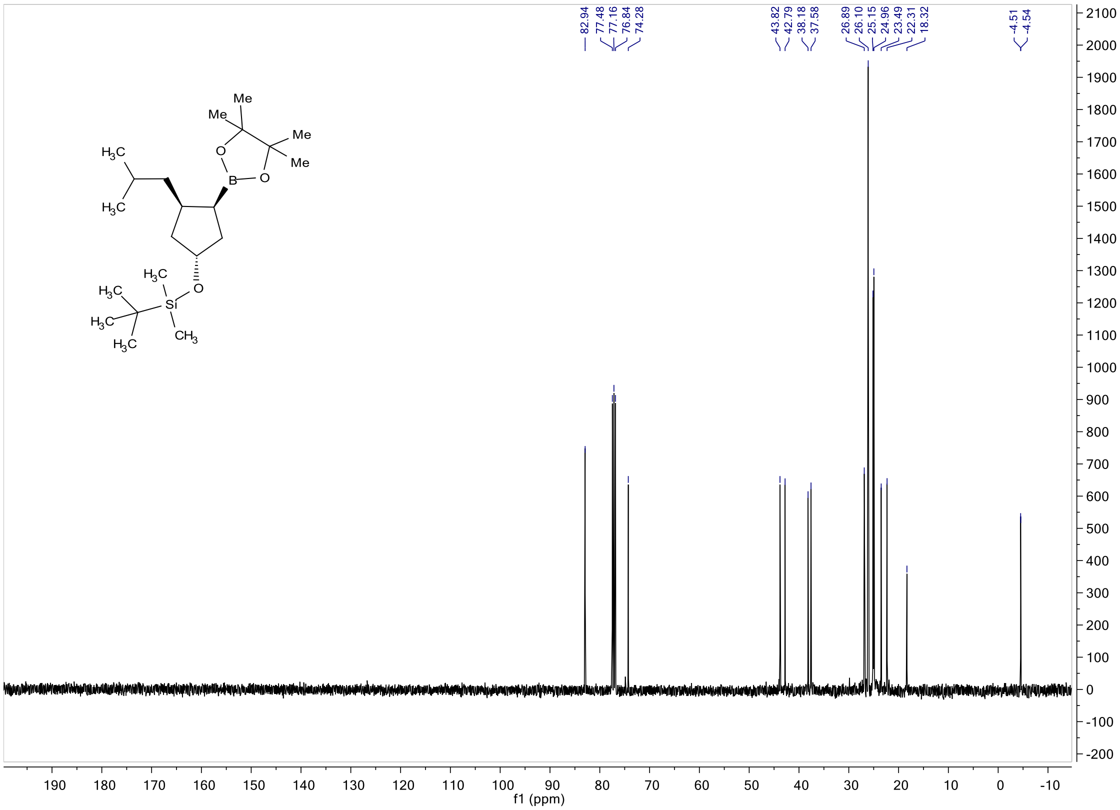

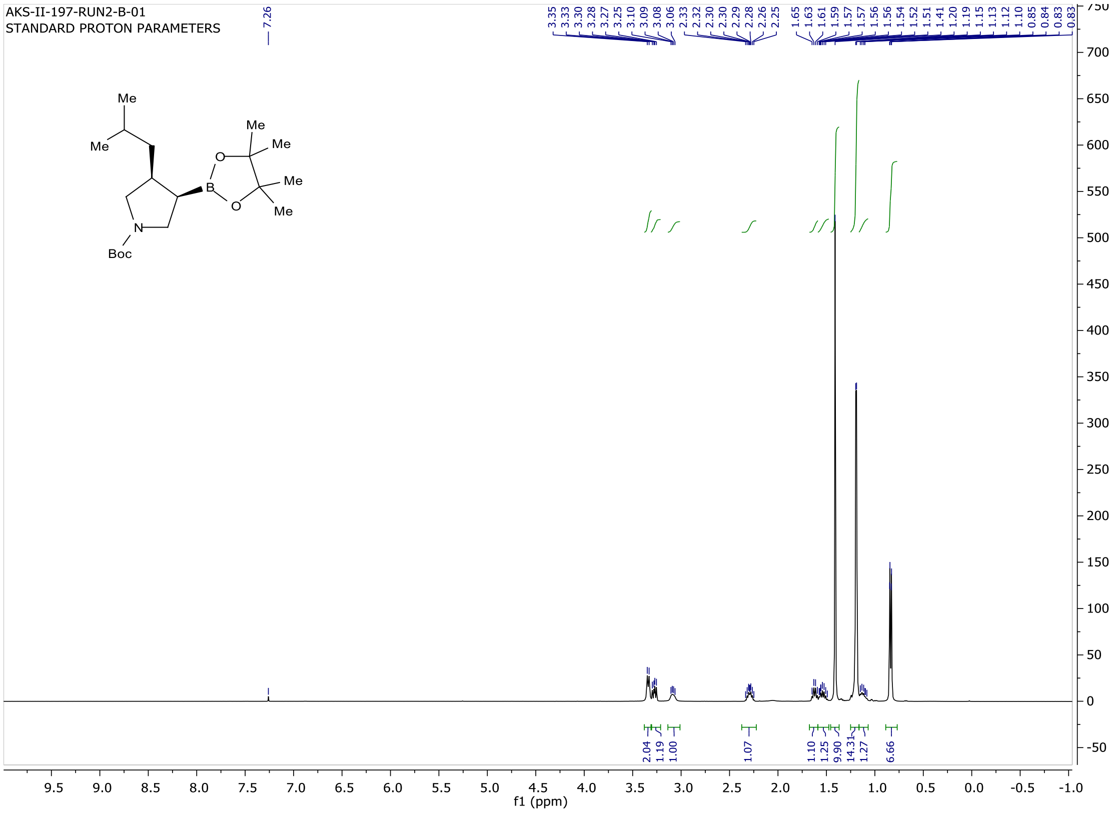
AKS-II-197-RUN2-B-01
STANDARD PROTON PARAMETERS
7.26
3.35 3.33 3.30 3.28 3.27 3.25 3.10 3.09 3.08 3.06 2.33 2.32 2.30 2.30 2.29 2.28 2.26 2.25 1.65 1.63 1.61 1.59 1.57 1.57 1.56 1.56 1.54 1.52 1.51 1.41 1.20 1.19 1.15 1.13 1.12 1.10 0.85 0.84 0.83
Me
Me
Me
Me
Me
Me
O
B
O
N
Boc
2.04 1.19 1.00 1.07 1.10 1.25 9.90 14.31 1.27 6.66
f1 (ppm)

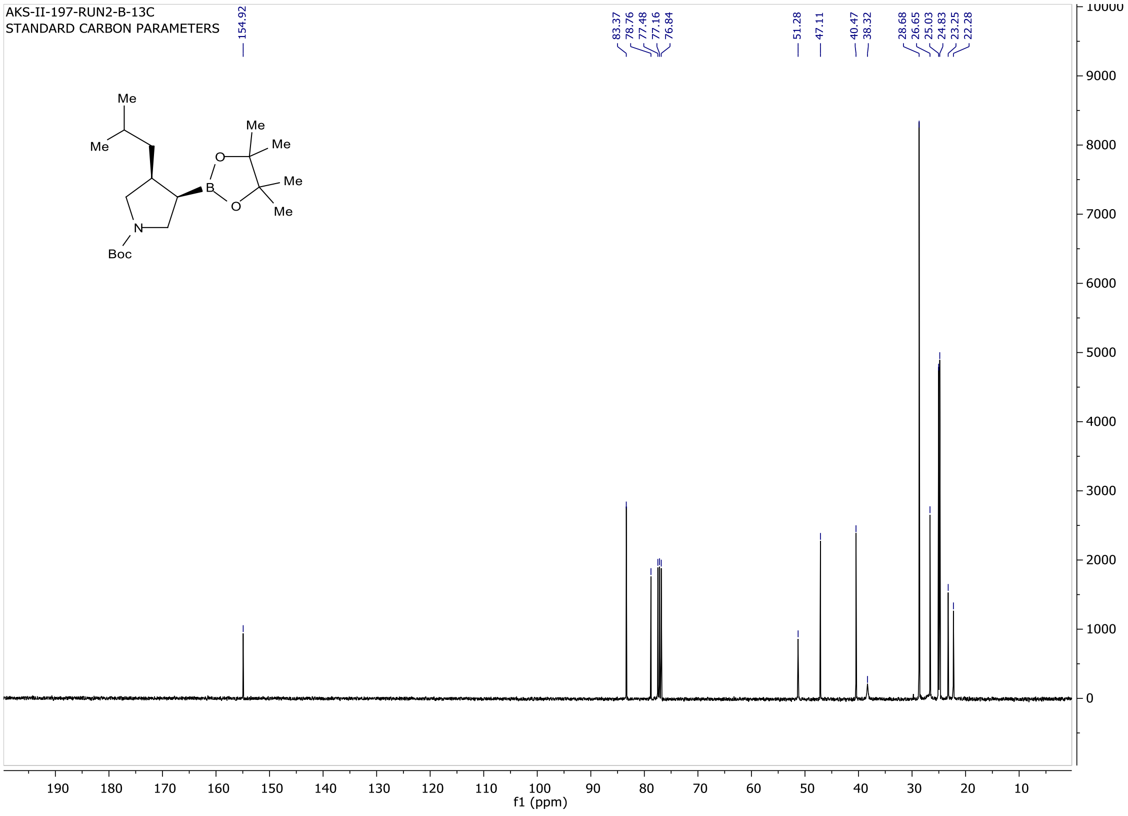
AKS-II-197-RUN2-B-13C
STANDARD CARBON PARAMETERS
154.92
83.37 78.76 77.48 77.16 76.84
51.28
47.11
40.47
38.32
28.68 26.65 25.03 24.83 23.25 22.28
Me
Me
Me
Me
Me
Me
O
B
O
N
Boc
f1 (ppm)

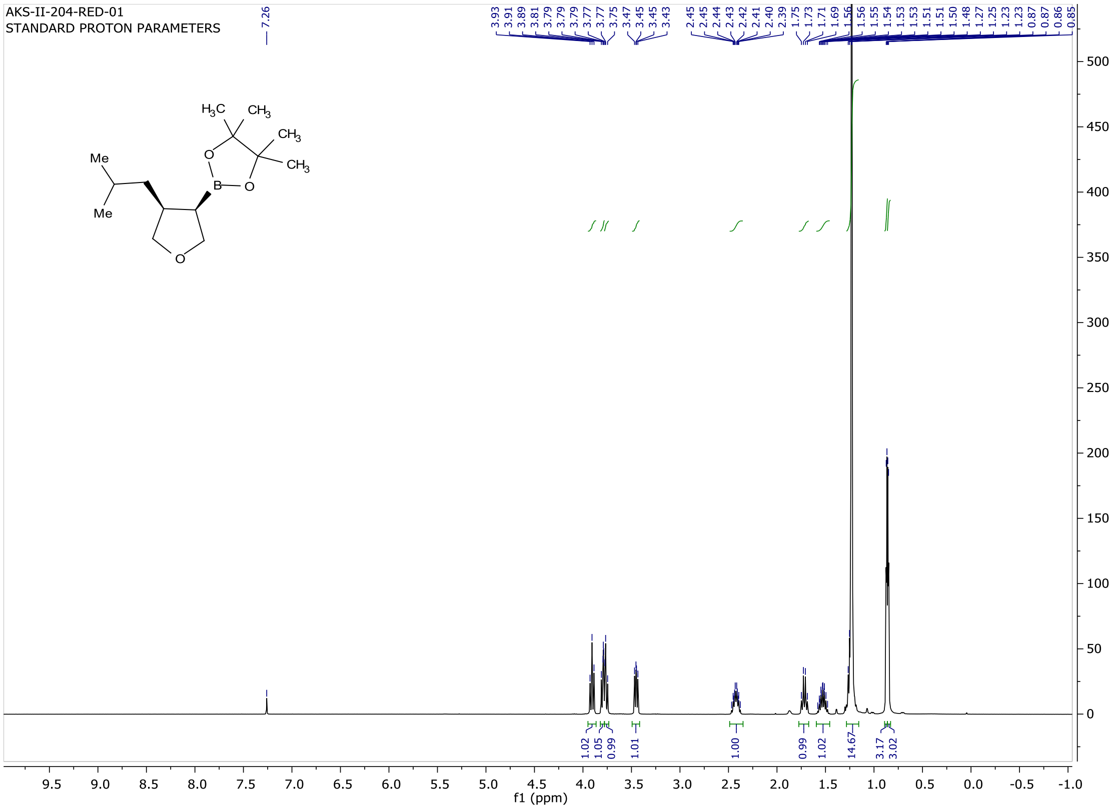

AKS-II-204-RED-01
STANDARD PROTON PARAMETERS
7.26
3.93 3.91 3.89 3.81 3.79 3.79 3.79 3.77 3.77 3.75 3.47 3.45 3.45 3.43
2.45 2.45 2.44 2.43 2.42 2.41 2.40 2.39 1.75 1.73 1.71 1.69 1.56 1.56 1.55 1.54 1.53 1.53 1.51 1.51 1.51 1.50 1.48 1.27 1.25 1.23 1.23 0.87 0.87 0.86 0.85
1.02 1.05 0.99 1.01 1.00 0.99 1.02 14.67 3.17 3.02
f1 (ppm)

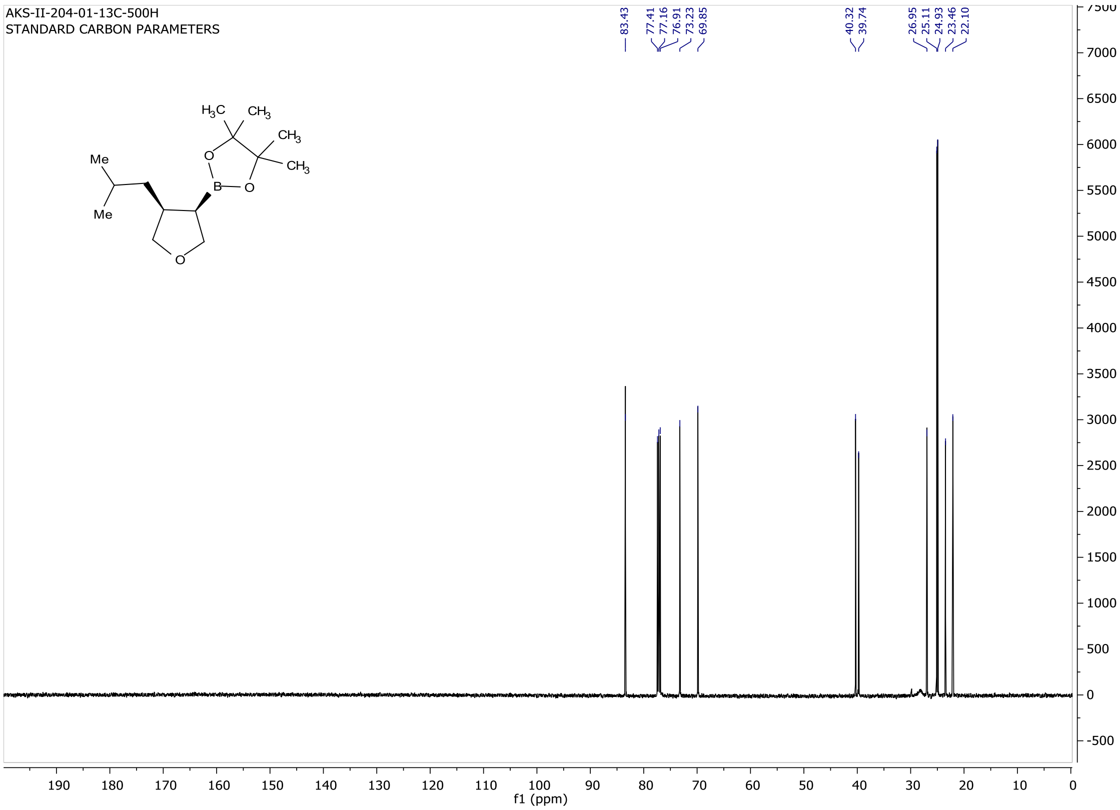

AKS-II-204-01-13C-500H
STANDARD CARBON PARAMETERS
83.43 77.41 77.16 76.91 73.23 69.85 40.32 39.74 26.95 25.11 24.93 23.46 22.10
f1 (ppm)

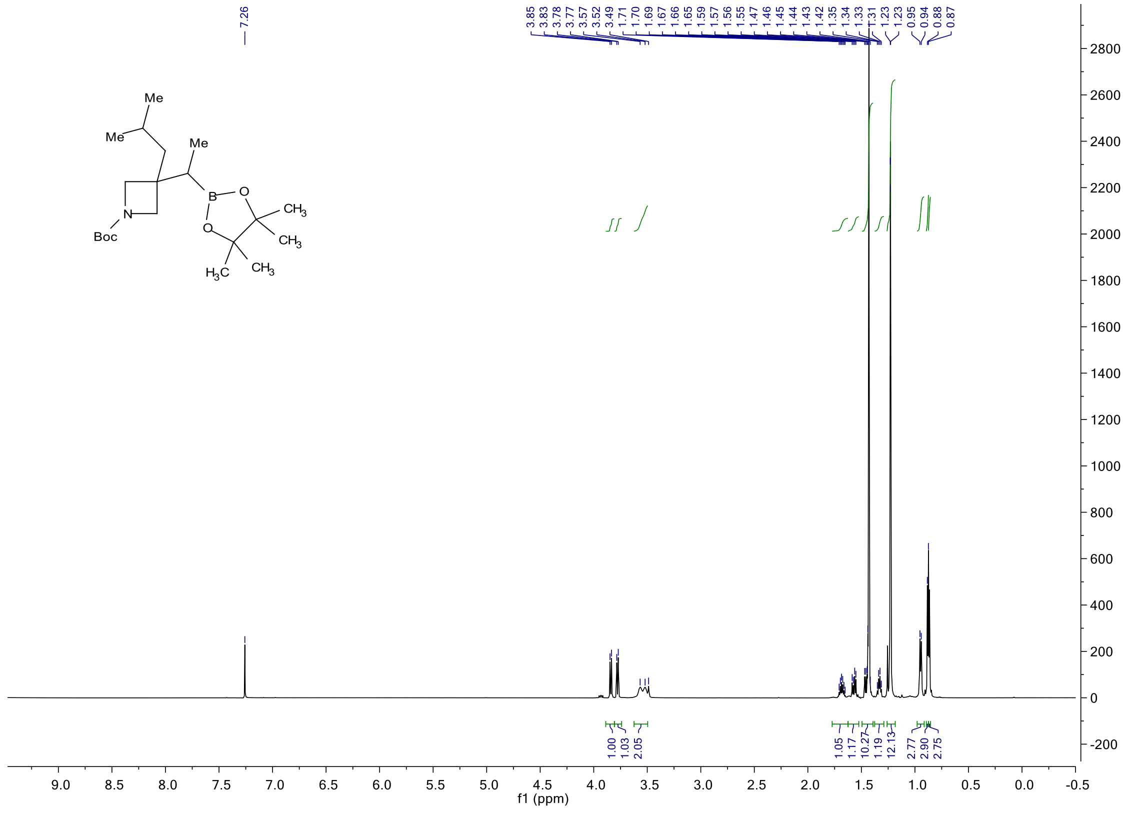

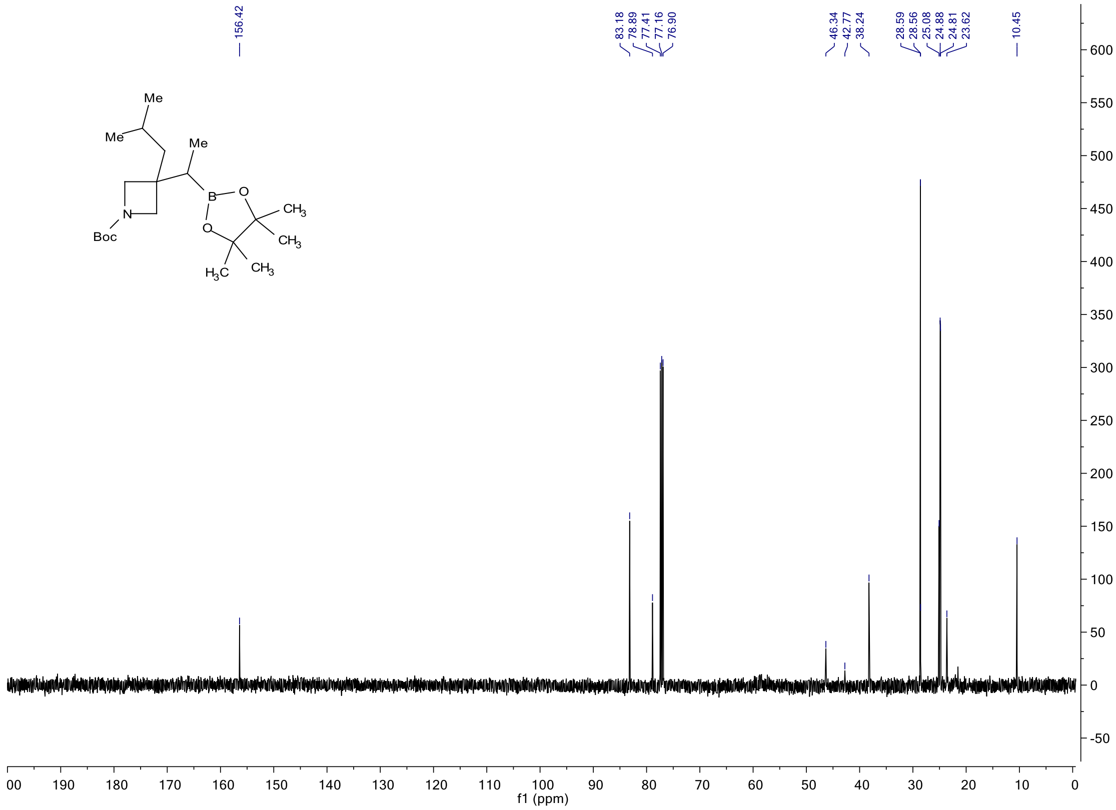

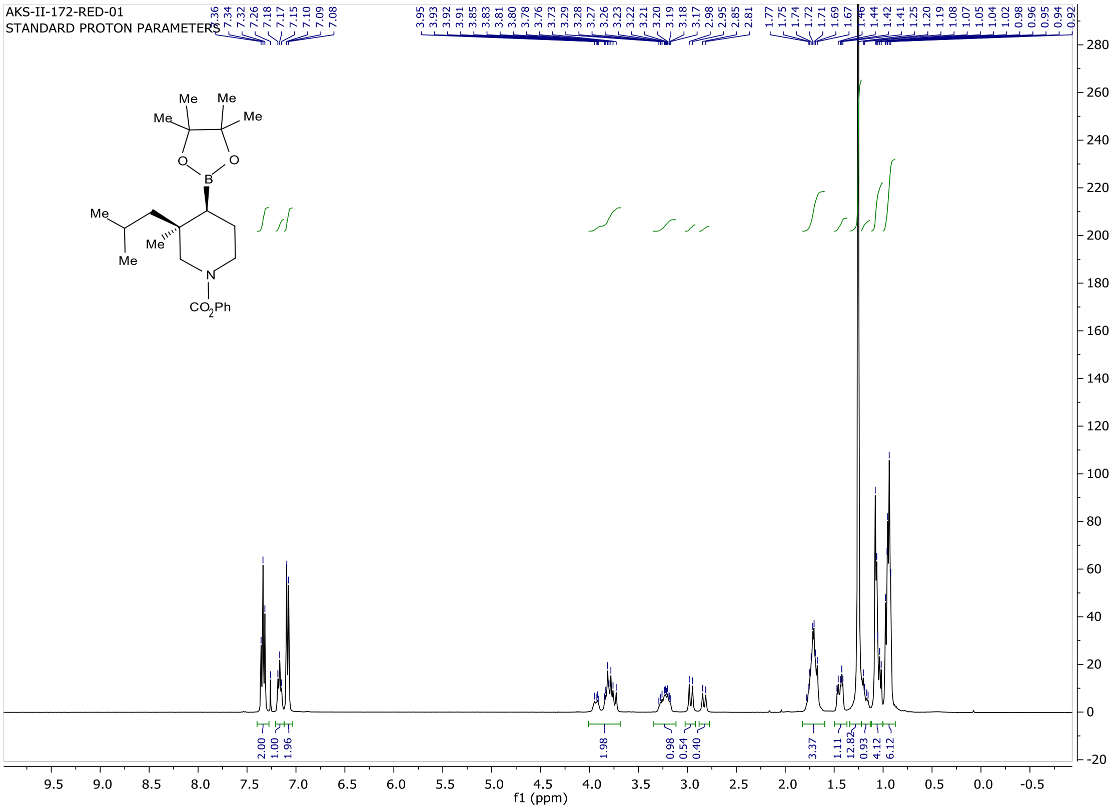
AKS-II-172-RED-01
STANDARD PROTON PARAMETERS
f1 (ppm)

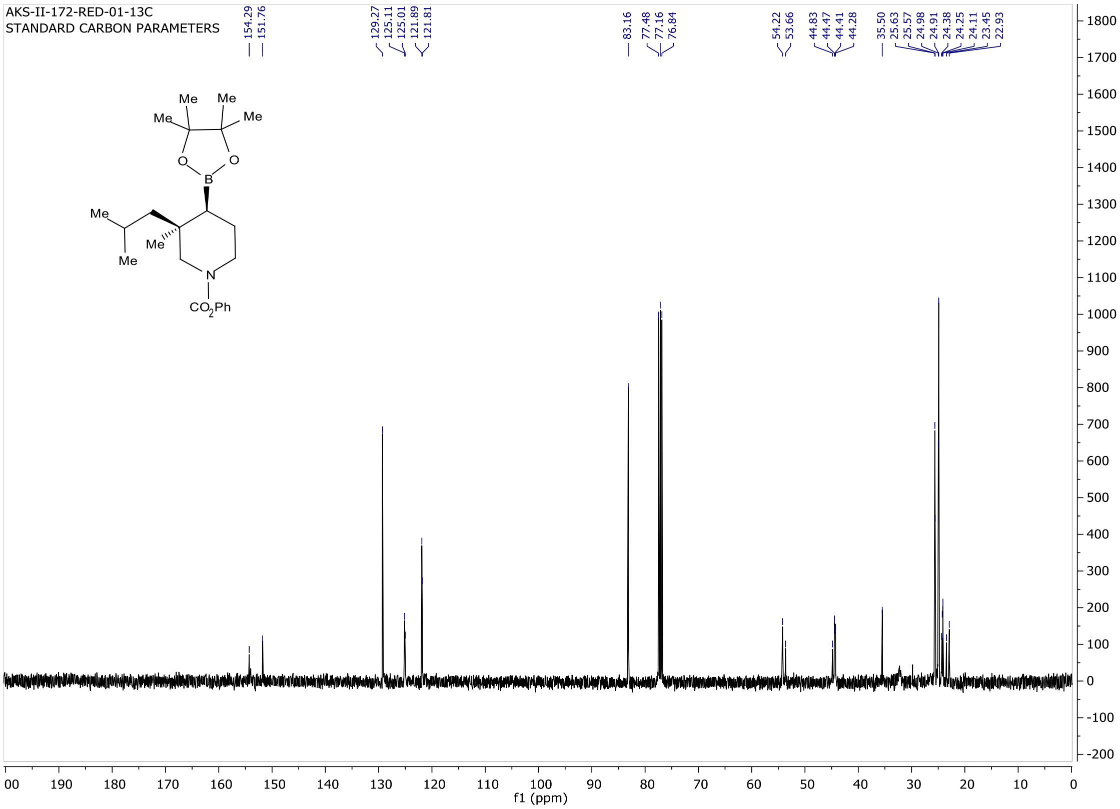
AKS-II-172-RED-01-13C
STANDARD CARBON PARAMETERS
f1 (ppm)

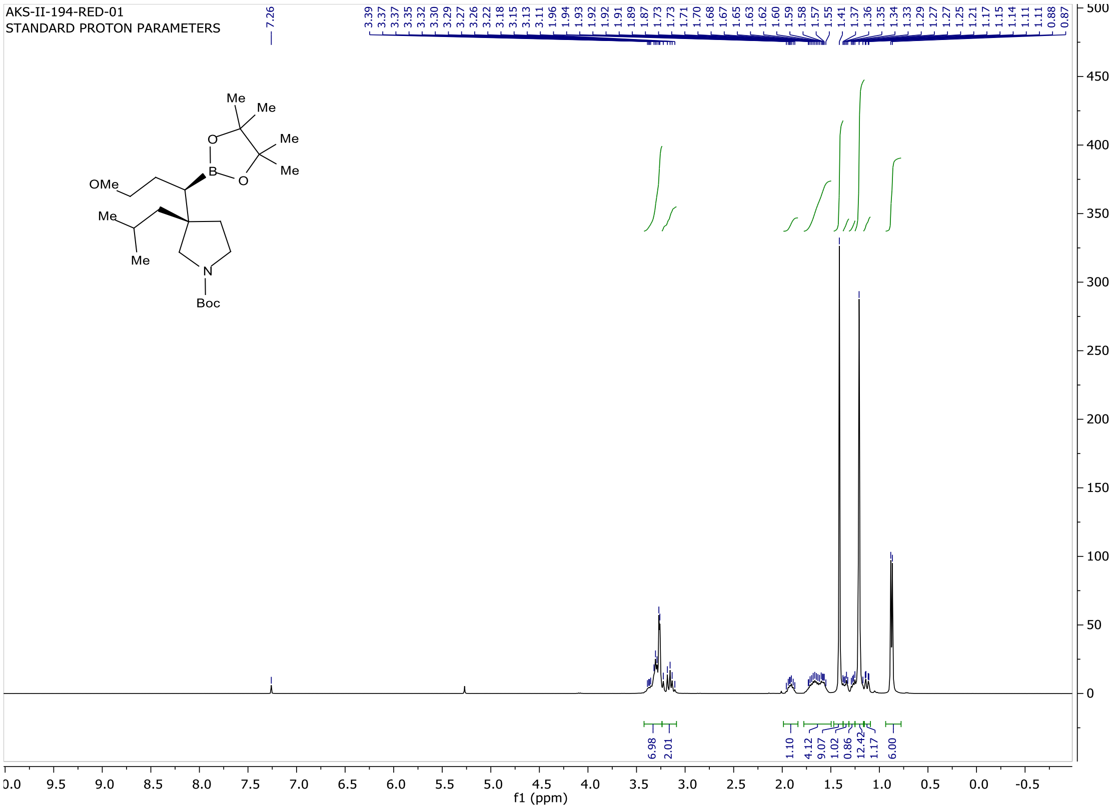
AKS-II-194-RED-01
STANDARD PROTON PARAMETERS
f1 (ppm)

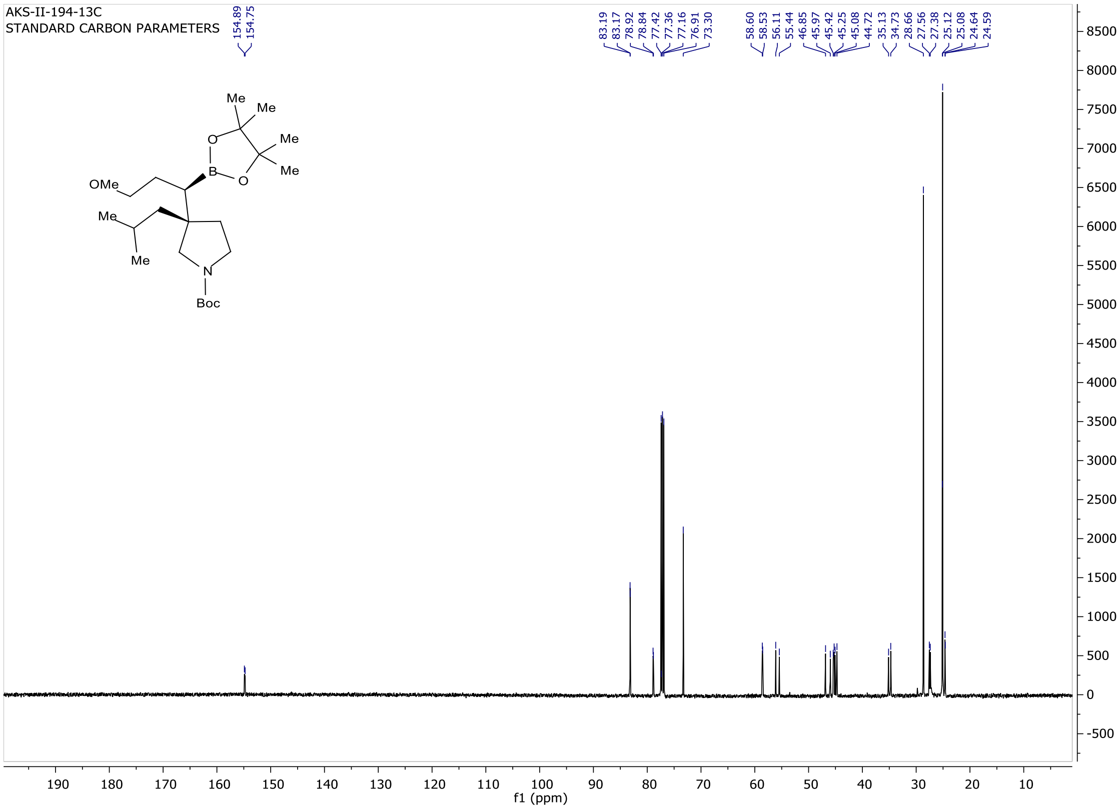
AKS-II-194-13C
STANDARD CARBON PARAMETERS
f1 (ppm)

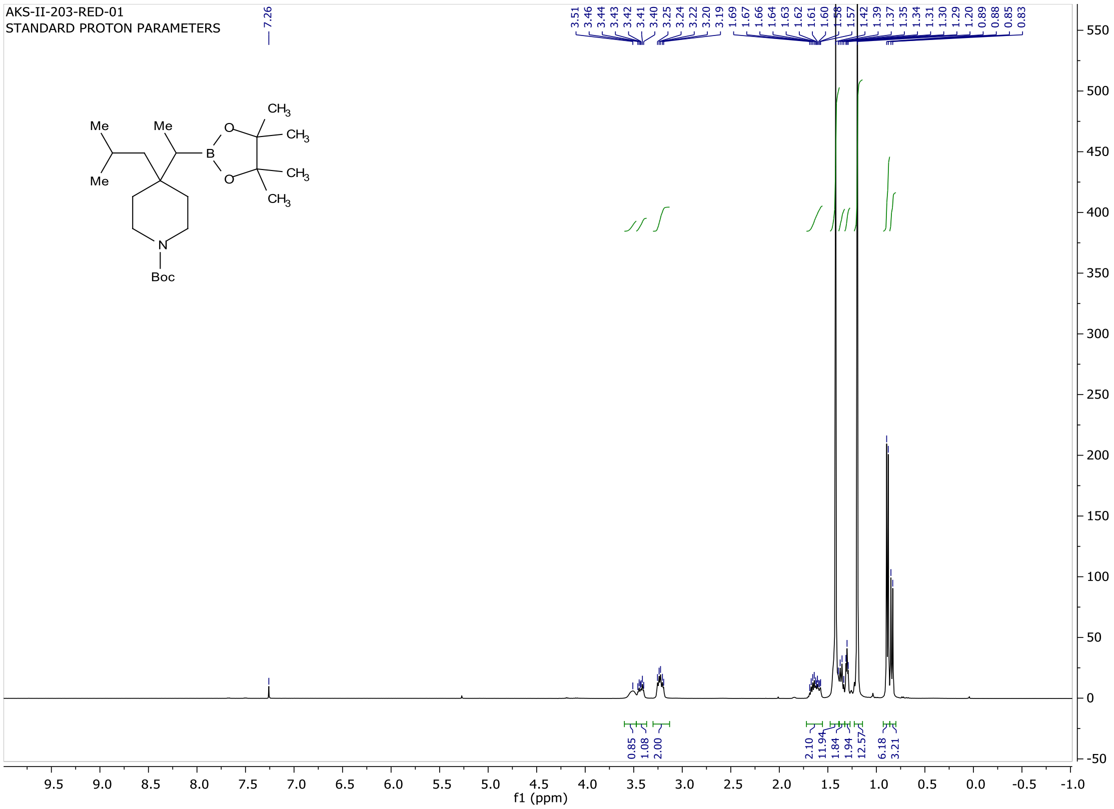

AKS-II-203-RED-01
STANDARD PROTON PARAMETERS
Boc
f1 (ppm)

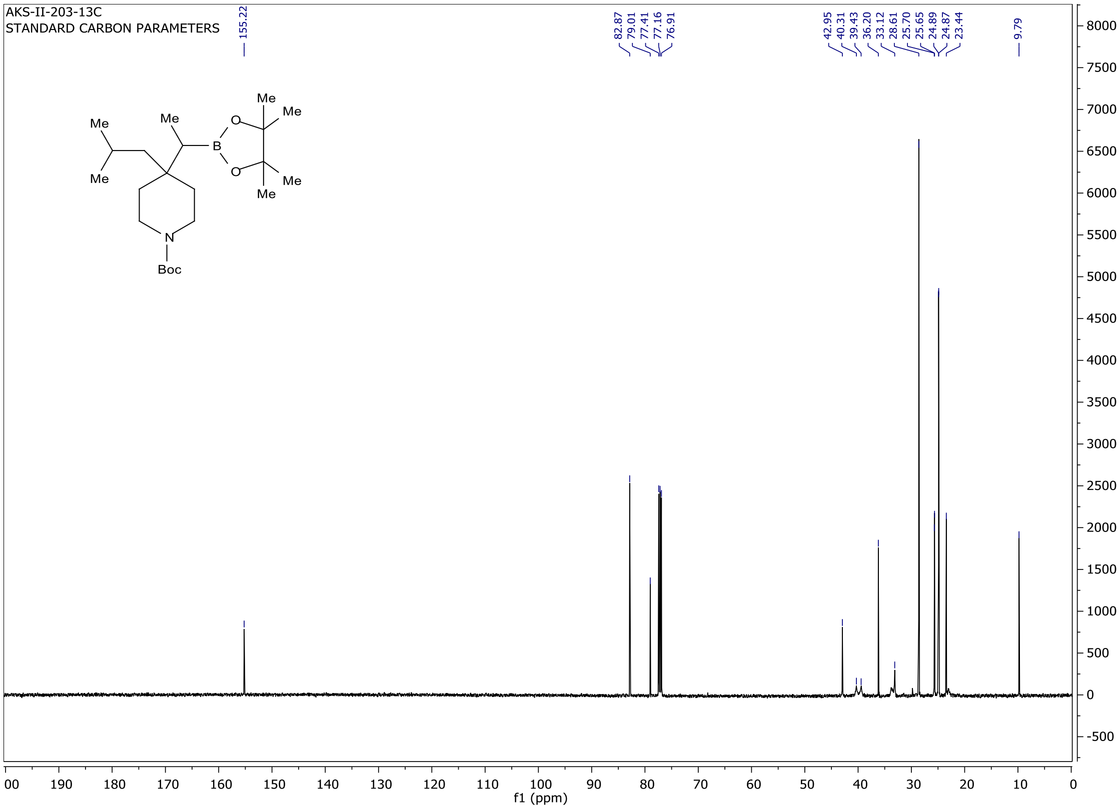

AKS-II-203-13C
STANDARD CARBON PARAMETERS
Boc
f1 (ppm)

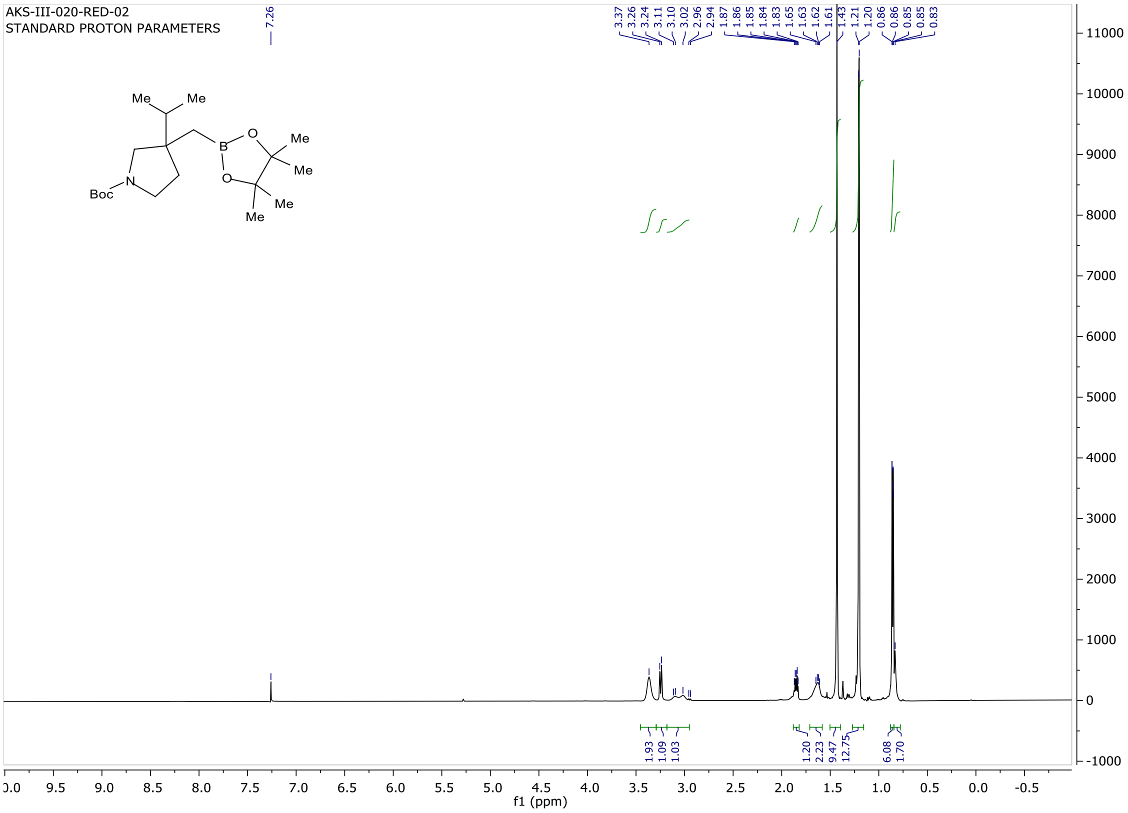
AKS-III-020-RED-02
STANDARD PROTON PARAMETERS
7.26
3.37
3.26
3.24
3.11
3.10
3.02
2.96
2.94
1.87
1.86
1.85
1.84
1.83
1.65
1.63
1.62
1.61
1.43
1.21
1.20
0.86
0.86
0.85
0.85
0.83
1.93
1.09
1.03
1.20
2.23
9.47
12.75
6.08
1.70
f1 (ppm)

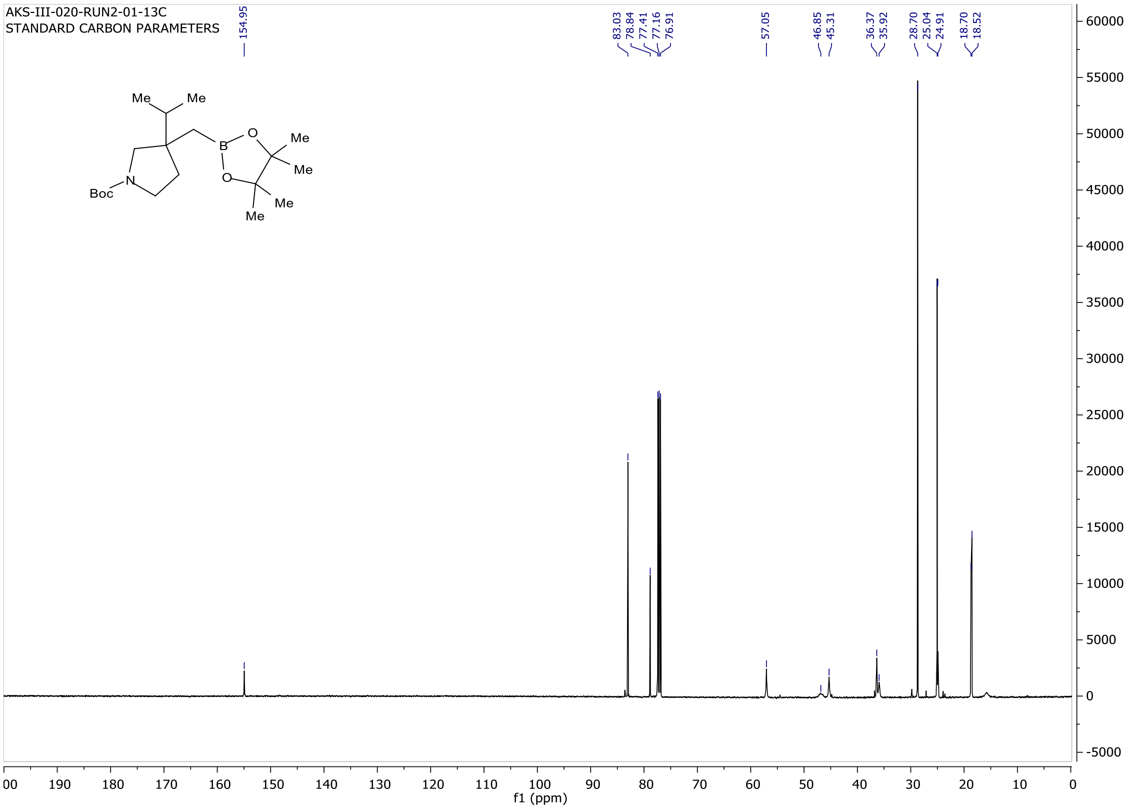
AKS-III-020-RUN2-01-13C
STANDARD CARBON PARAMETERS
154.95
83.03
78.84
77.41
77.16
76.91
57.05
46.85
45.31
36.37
35.92
28.70
25.04
24.91
18.70
18.52
f1 (ppm)

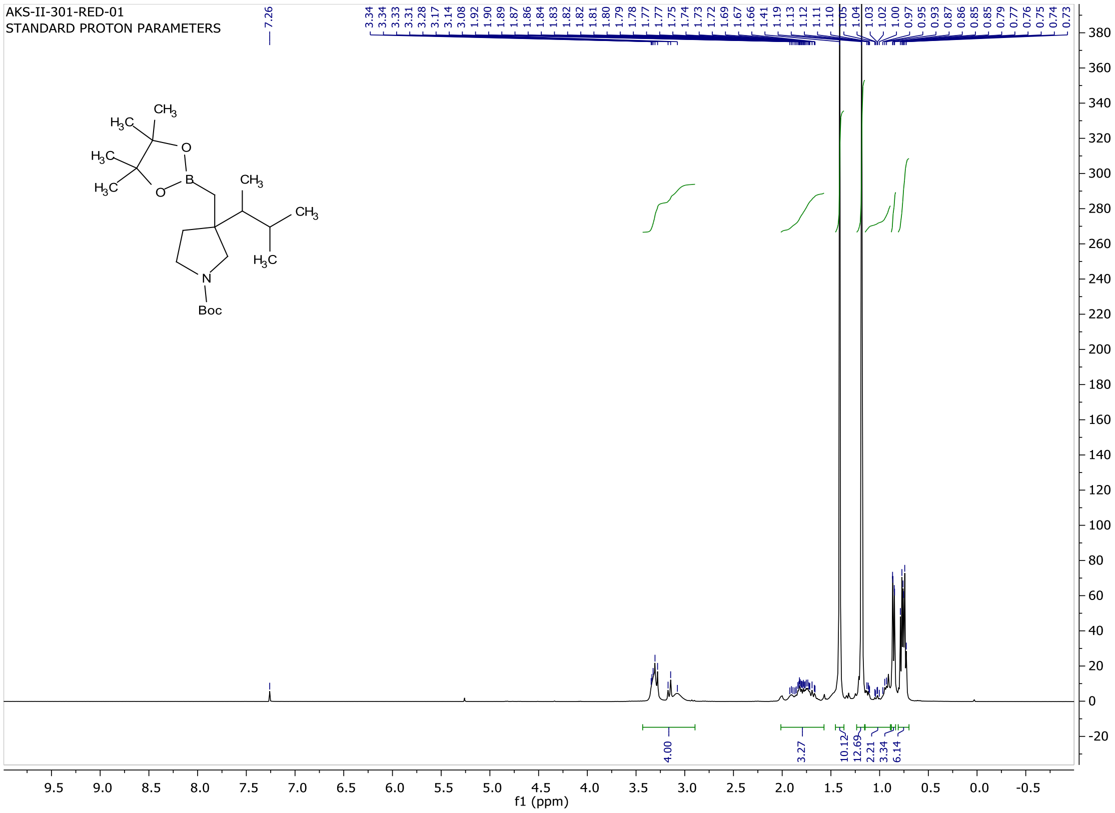
AKS-II-301-RED-01
STANDARD PROTON PARAMETERS
f1 (ppm)

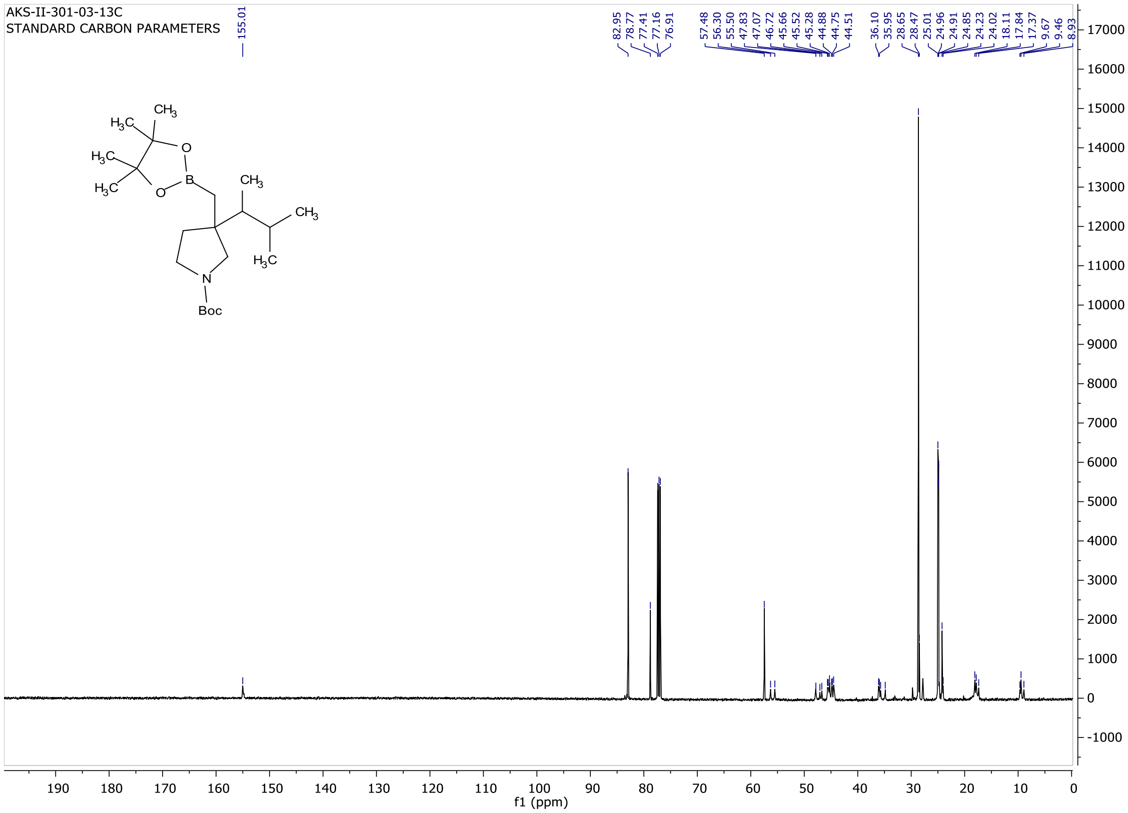
AKS-II-301-03-13C
STANDARD CARBON PARAMETERS
f1 (ppm)

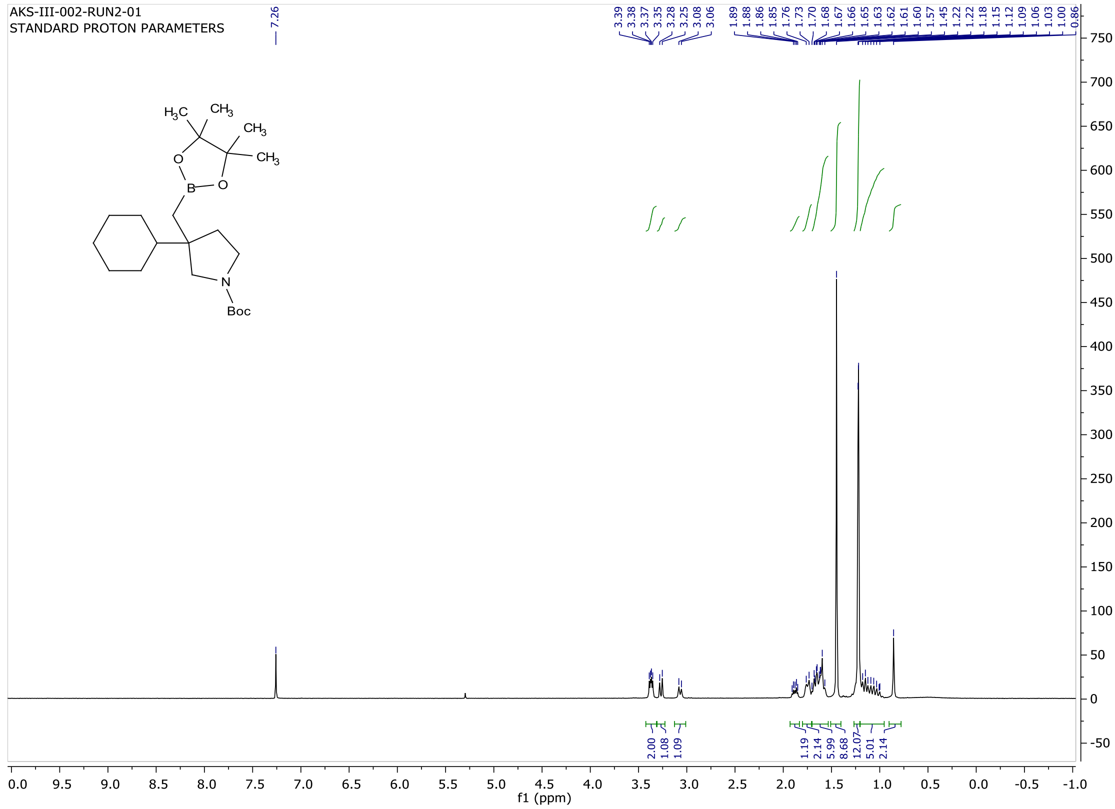

AKS-III-002-RUN2-01
STANDARD PROTON PARAMETERS
Boc
f1 (ppm)

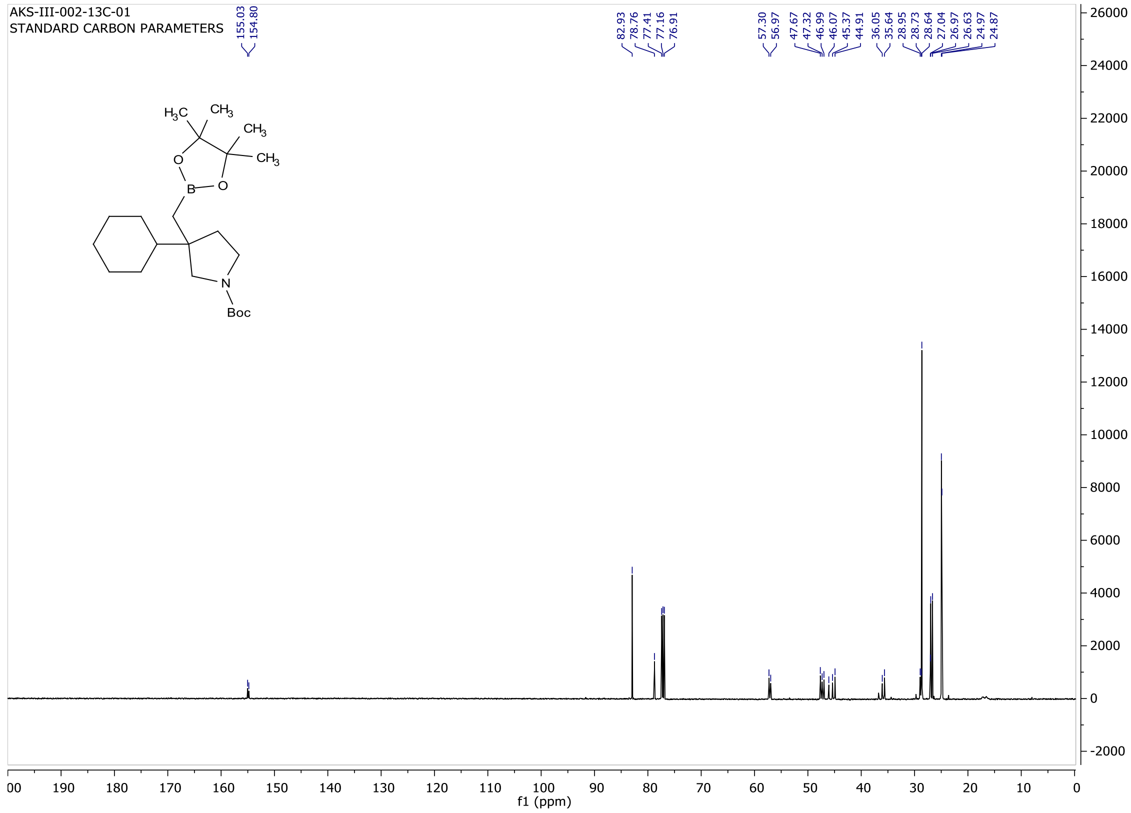

AKS-III-002-13C-01
STANDARD CARBON PARAMETERS
Boc
f1 (ppm)

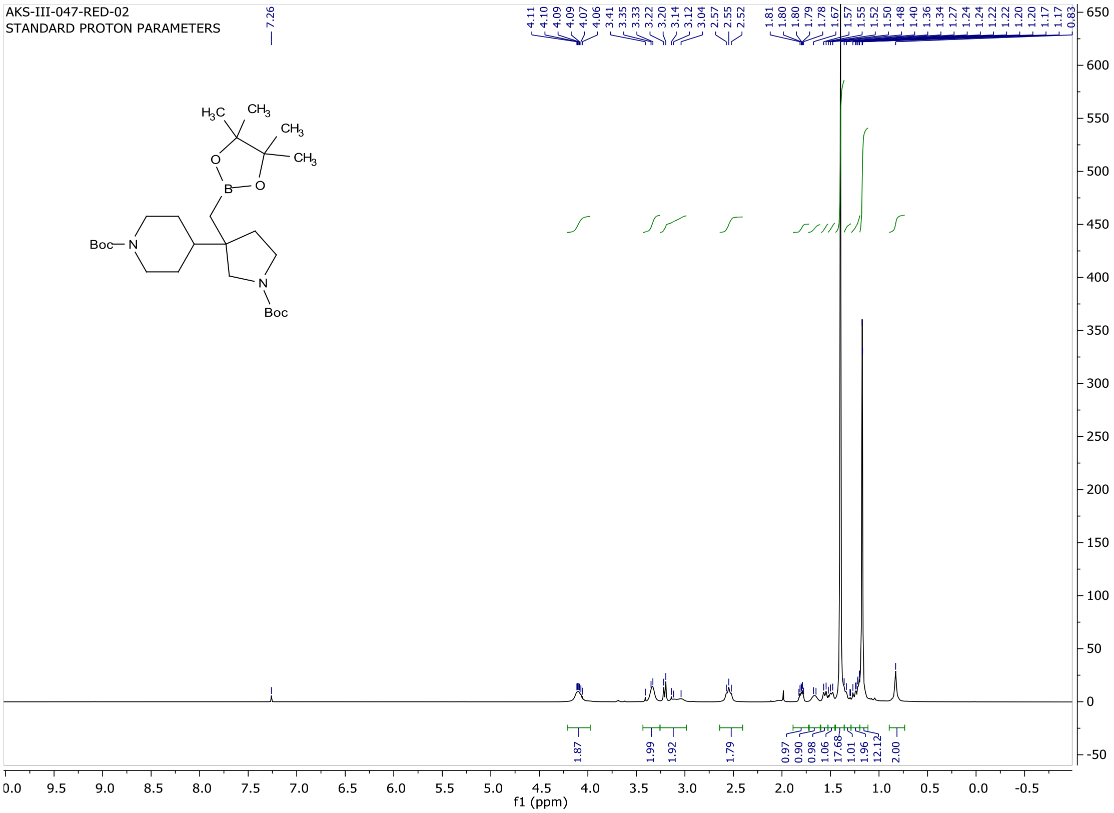
AKS-III-047-RED-02
STANDARD PROTON PARAMETERS
7.26
4.11 4.10 4.09 4.09 4.07 4.06 3.41 3.35 3.33 3.22 3.20 3.14 3.12 3.04 2.57 2.55 2.52 1.81 1.80 1.80 1.79 1.78 1.67 1.57 1.55 1.52 1.50 1.48 1.40 1.36 1.34 1.27 1.24 1.24 1.22 1.22 1.20 1.20 1.17 1.17 0.83
1.87 1.99 1.92 1.79 0.97 0.90 0.98 1.06 17.68 1.01 1.96 12.12 2.00
Boc
N
O
B
H3C
CH3
f1 (ppm)

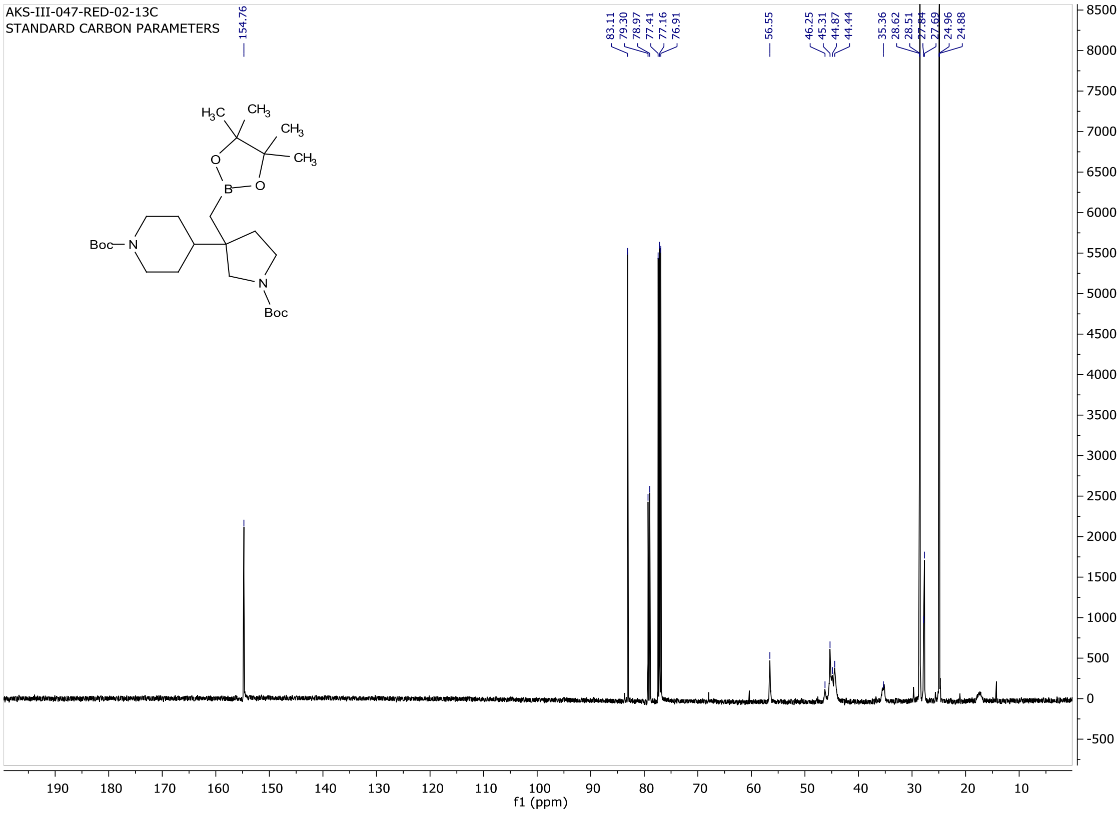
AKS-III-047-RED-02-13C
STANDARD CARBON PARAMETERS
154.76
83.11 79.30 78.97 77.41 77.16 76.91
56.55
46.25 45.31 44.87 44.44
35.36 28.62 28.51 27.84 27.69 24.96 24.88
Boc
N
O
B
H3C
CH3
f1 (ppm)

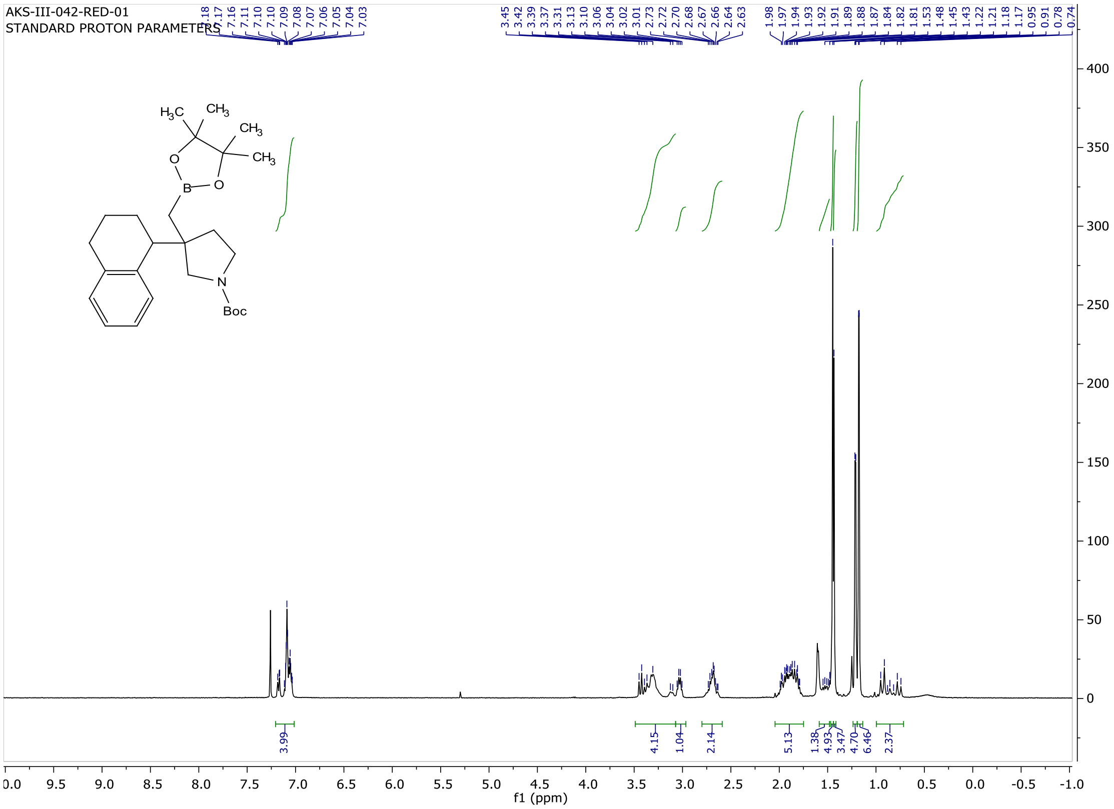
AKS-III-042-RED-01
STANDARD PROTON PARAMETERS
f1 (ppm)

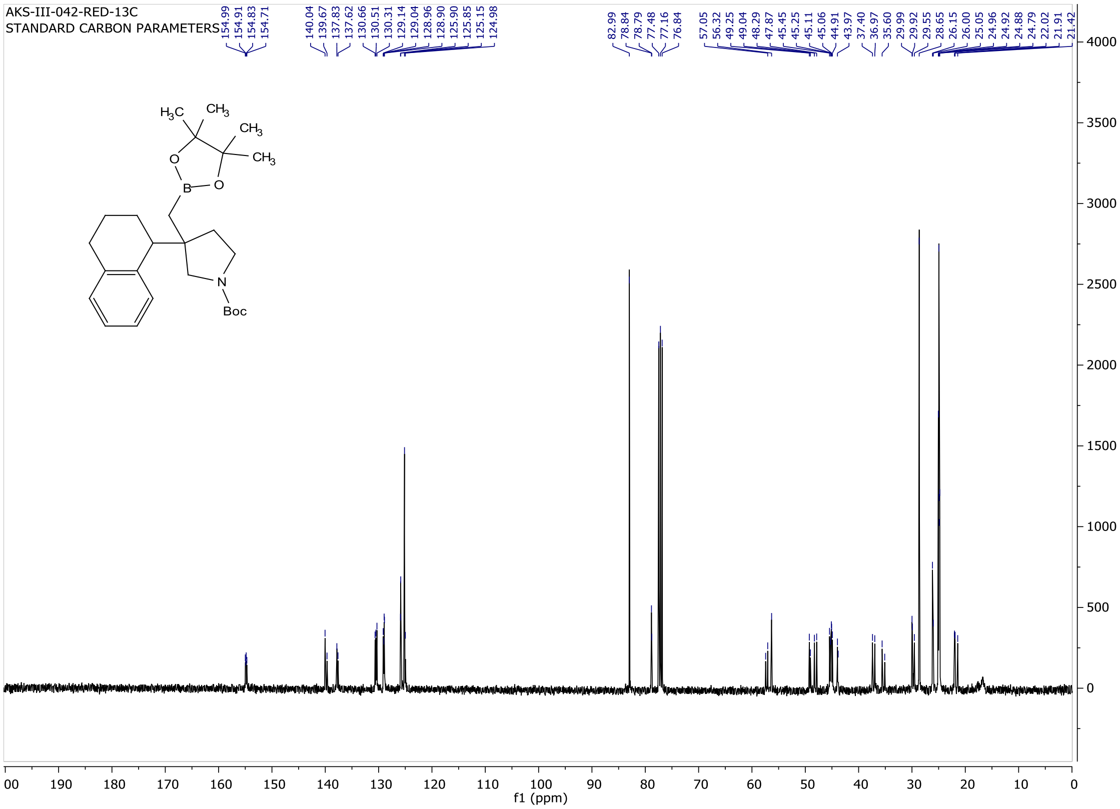
AKS-III-042-RED-13C
STANDARD CARBON PARAMETERS
f1 (ppm)

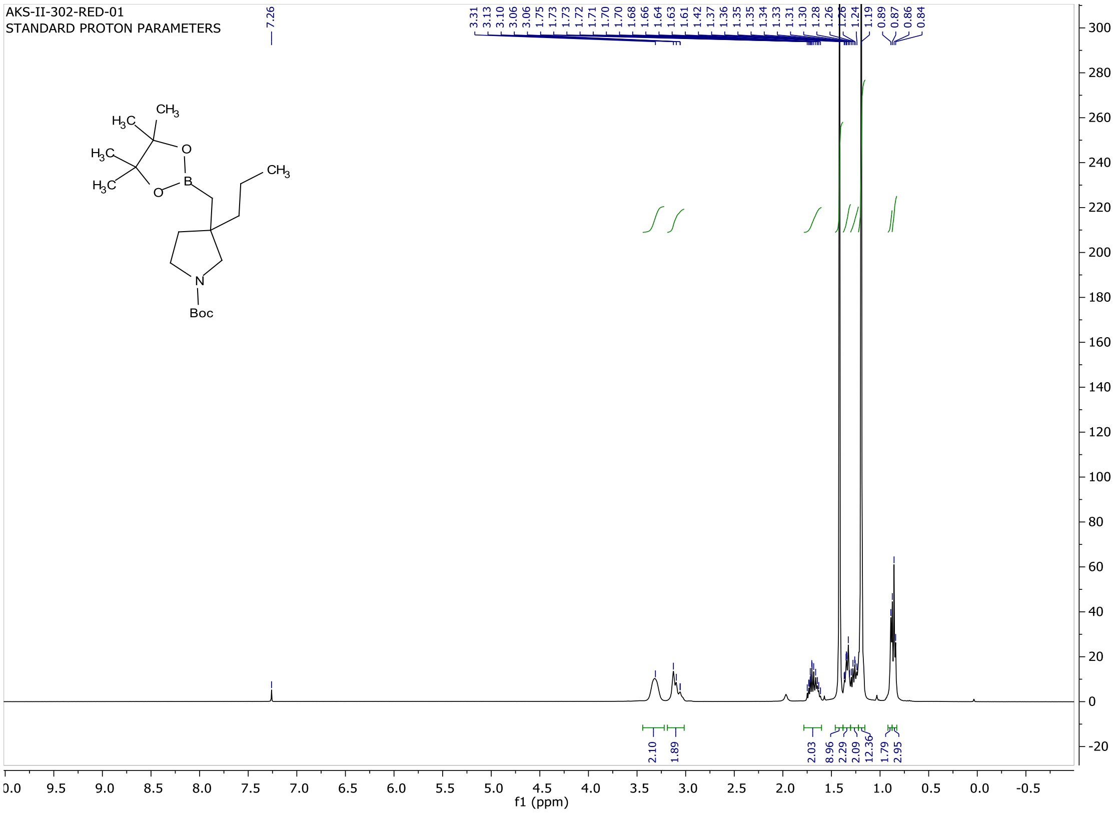
AKS-II-302-RED-01
STANDARD PROTON PARAMETERS
Boc
f1 (ppm)

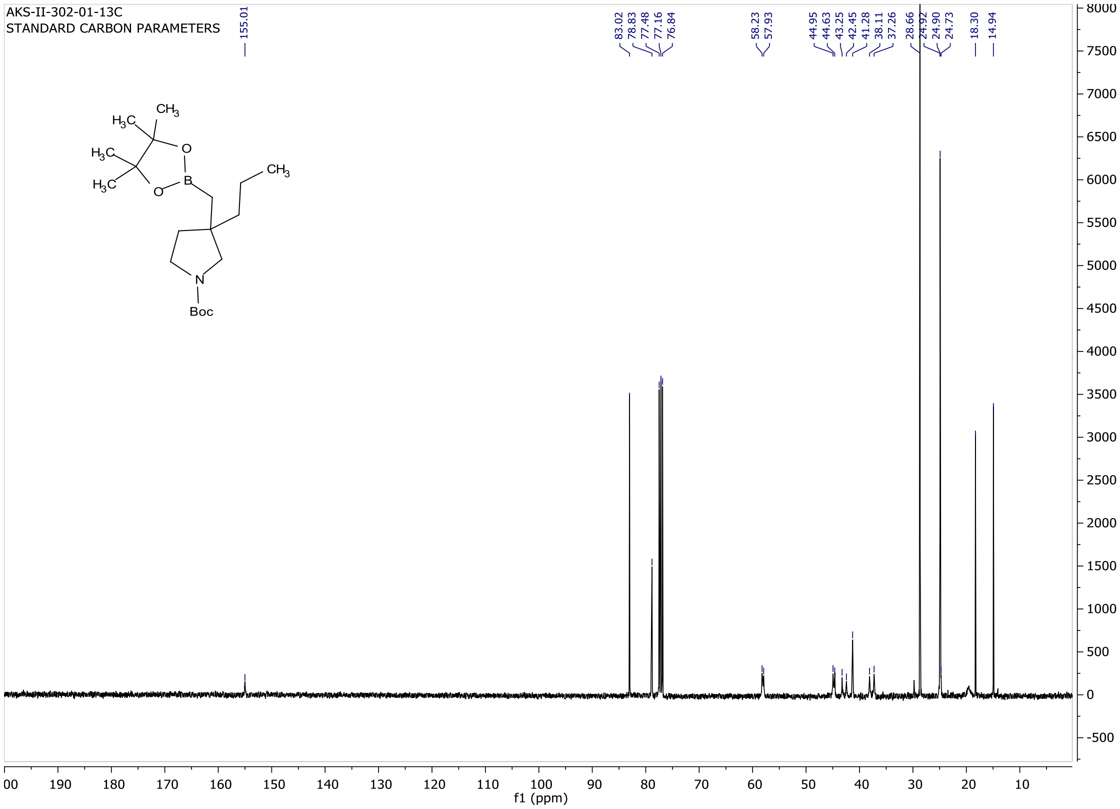
AKS-II-302-01-13C
STANDARD CARBON PARAMETERS
Boc
f1 (ppm)

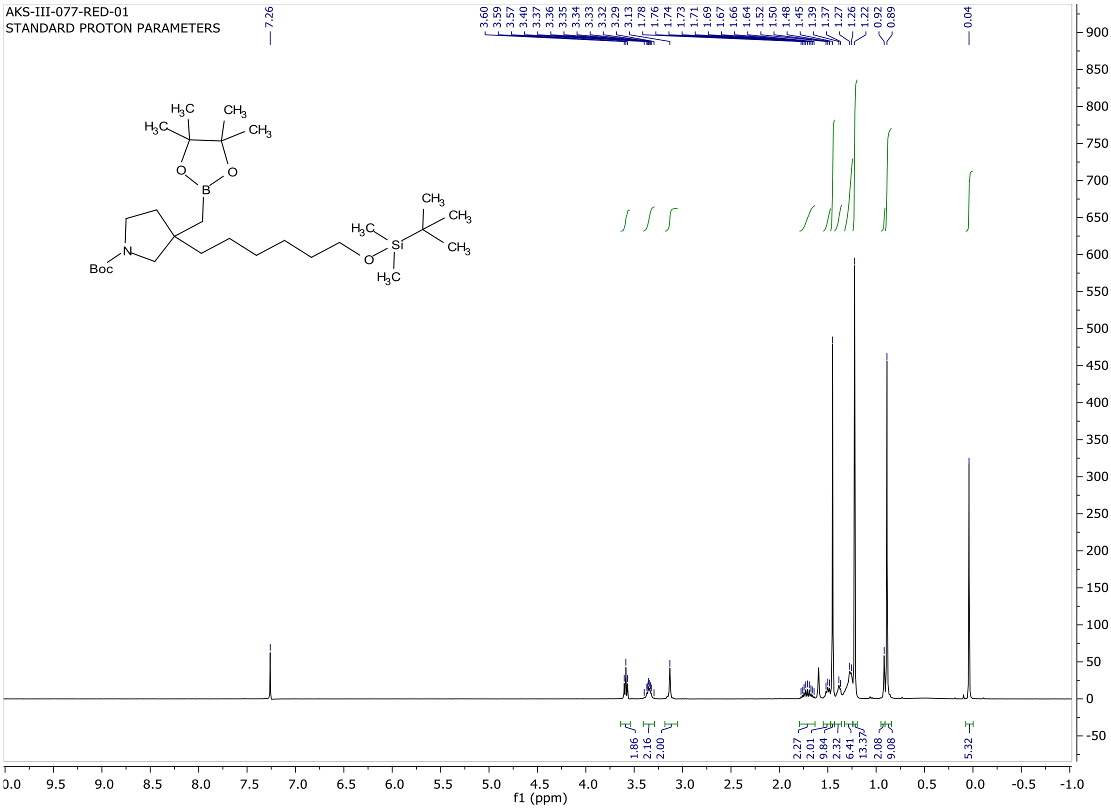
AKS-III-077-RED-01
STANDARD PROTON PARAMETERS
7.26
3.60 3.59 3.57 3.40 3.37 3.36 3.35 3.34 3.33 3.32 3.29 3.13 1.78 1.76 1.74 1.73 1.71 1.69 1.67 1.66 1.64 1.52 1.50 1.48 1.45 1.39 1.37 1.27 1.26 1.22 0.92 0.89
0.04
1.86 2.16 2.00 2.27 2.01 9.84 2.32 6.41 13.37 2.08 9.08 5.32
f1 (ppm)
Boc

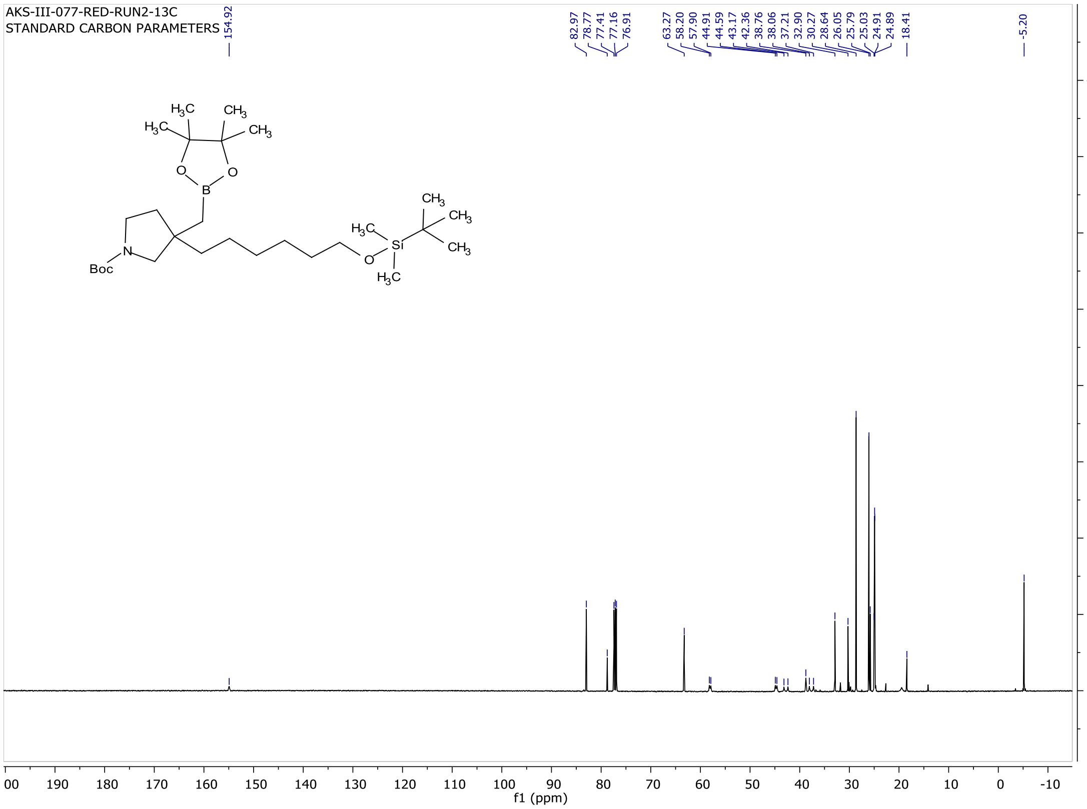
AKS-III-077-RED-RUN2-13C
STANDARD CARBON PARAMETERS
154.92
82.97 78.77 77.41 77.16 76.91
63.27 58.20 57.90 44.91 44.59 43.17 42.36 38.76 38.06 37.21 32.90 30.27 28.64 26.05 25.79 25.03 24.91 24.89 18.41
-5.20
f1 (ppm)
Boc

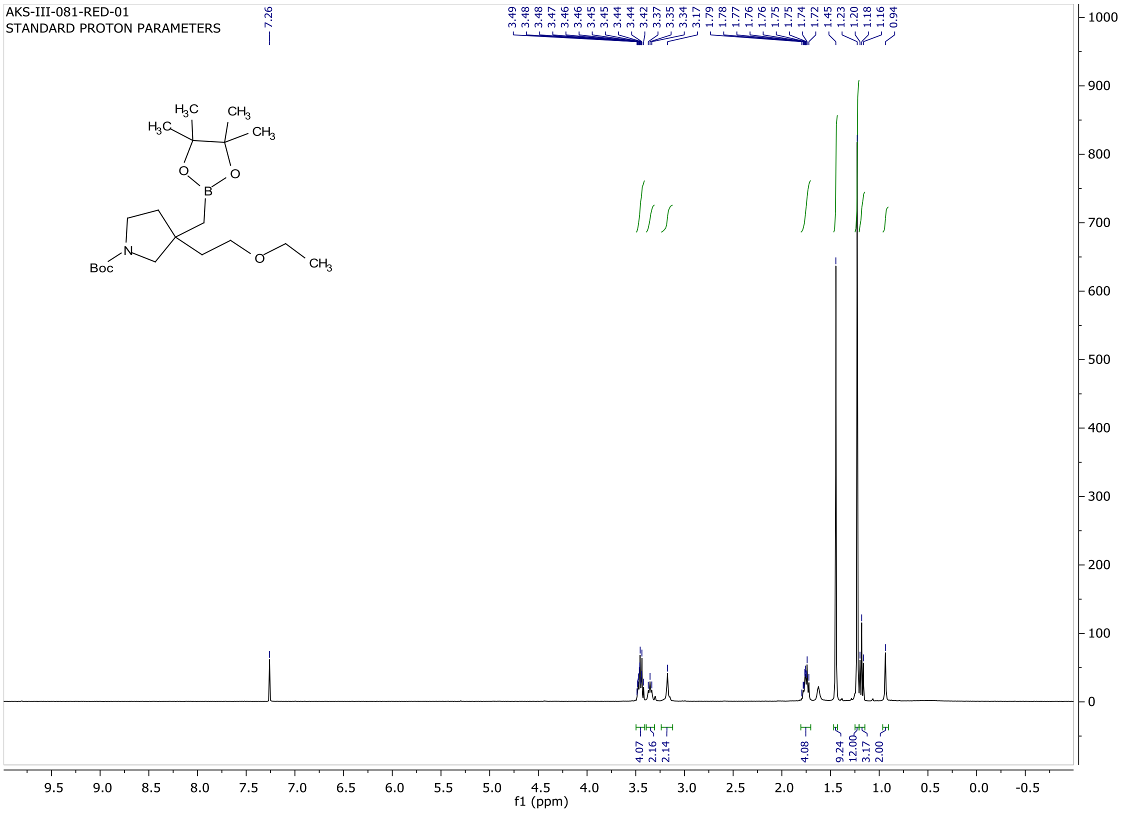
AKS-III-081-RED-01
STANDARD PROTON PARAMETERS
7.26
3.49
3.48
3.48
3.47
3.46
3.46
3.45
3.45
3.44
3.44
3.42
3.37
3.35
3.34
3.17
1.79
1.78
1.77
1.76
1.76
1.75
1.75
1.74
1.72
1.45
1.23
1.20
1.18
1.16
0.94
4.07
2.16
2.14
4.08
9.24
12.00
3.17
2.00
f1 (ppm)
Boc

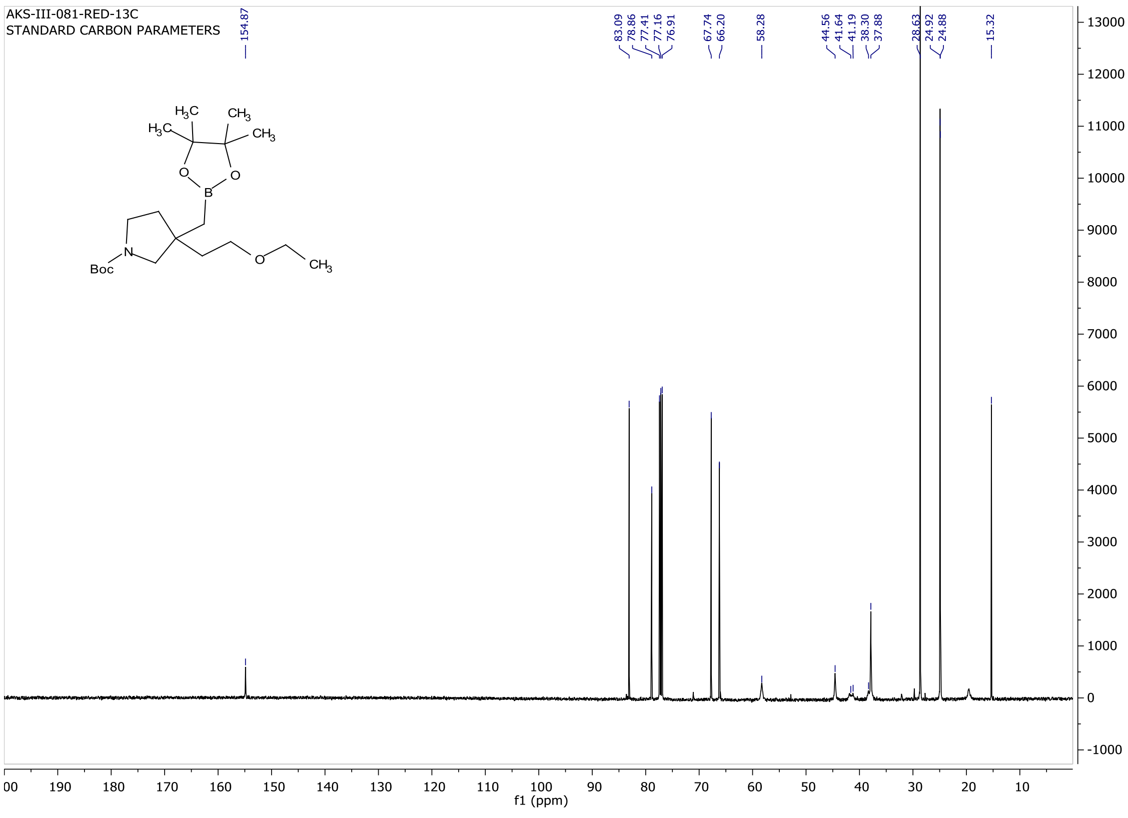
AKS-III-081-RED-13C
STANDARD CARBON PARAMETERS
154.87
83.09
78.86
77.41
77.16
76.91
67.74
66.20
58.28
44.56
41.64
41.19
38.30
37.88
28.63
24.92
24.88
15.32
f1 (ppm)
Boc

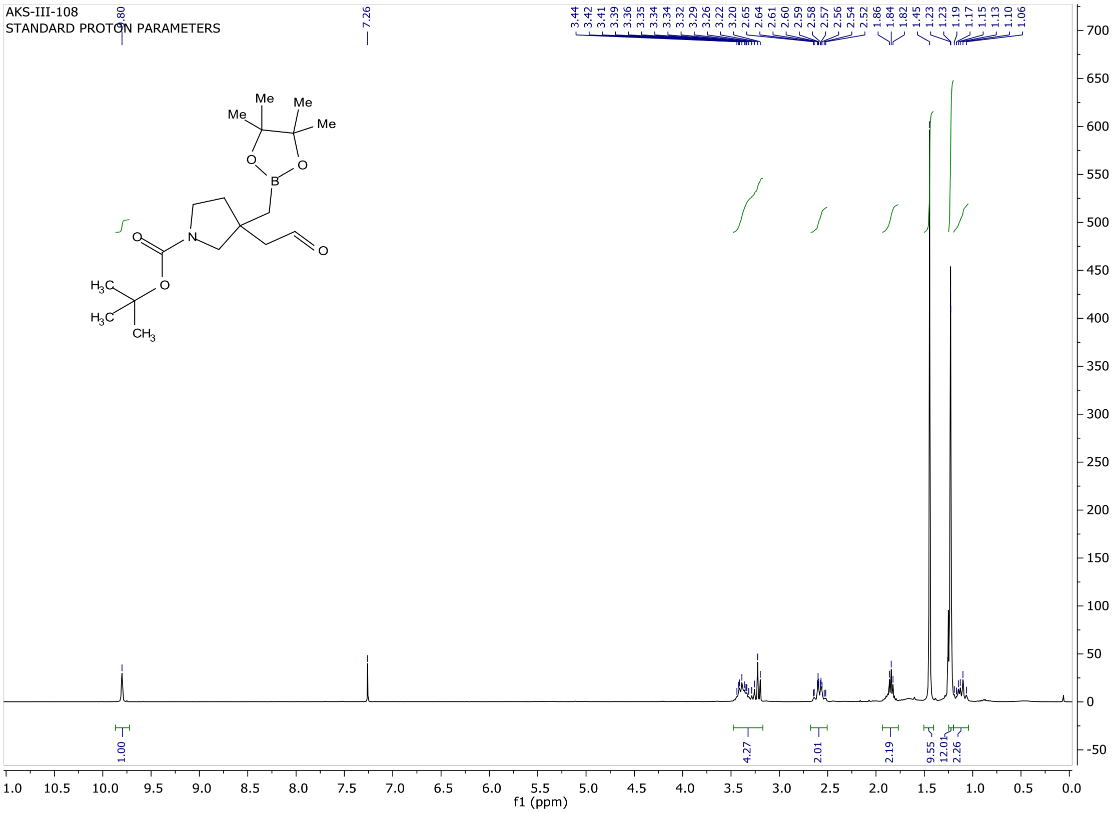
AKS-III-108
STANDARD PROTON PARAMETERS
9.80
7.26
3.44 3.42 3.41 3.39 3.36 3.35 3.34 3.34 3.32 3.29 3.26 3.22 3.20 2.65 2.64 2.61 2.60 2.59 2.58 2.57 2.56 2.54 2.52 1.86 1.84 1.82 1.45 1.23 1.23 1.19 1.17 1.15 1.13 1.10 1.06
Me
Me
Me
Me
O
O
B
N
O
O
O
H_3C
H_3C
CH_3
1.00
4.27
2.01
2.19
9.55
12.01
2.26
f1 (ppm)

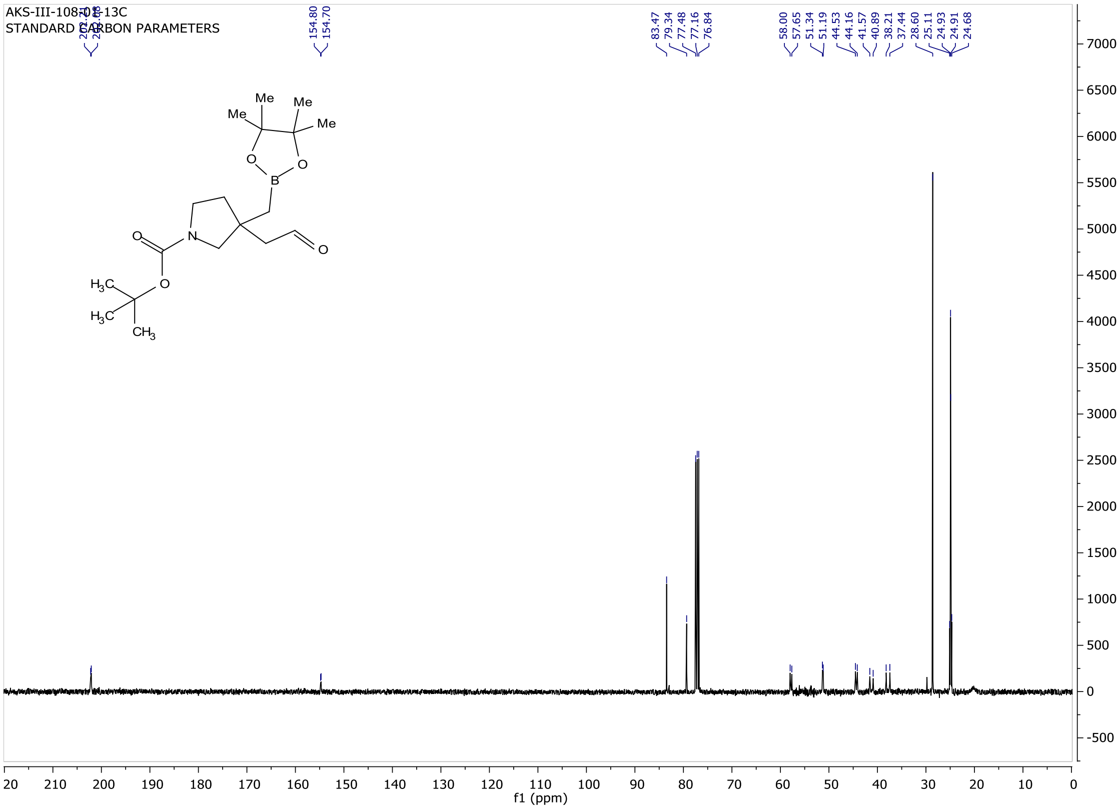
AKS-III-108-01-13C
STANDARD CARBON PARAMETERS
202.21 202.08
154.80 154.70
83.47 79.34 77.48 77.16 76.84
58.00 57.65 51.34 51.19 44.53 44.16 41.57 40.89 38.21 37.44 28.60 25.11 24.93 24.91 24.68
Me
Me
Me
Me
O
O
B
N
O
O
O
H_3C
H_3C
CH_3
f1 (ppm)

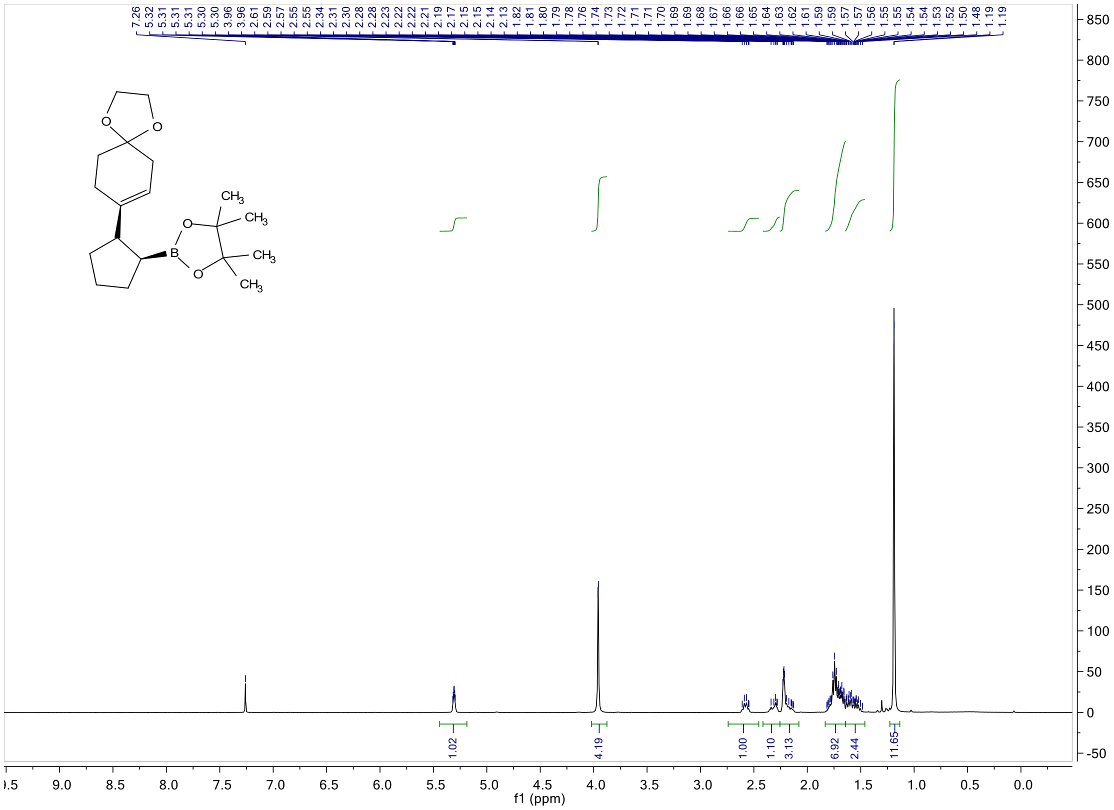

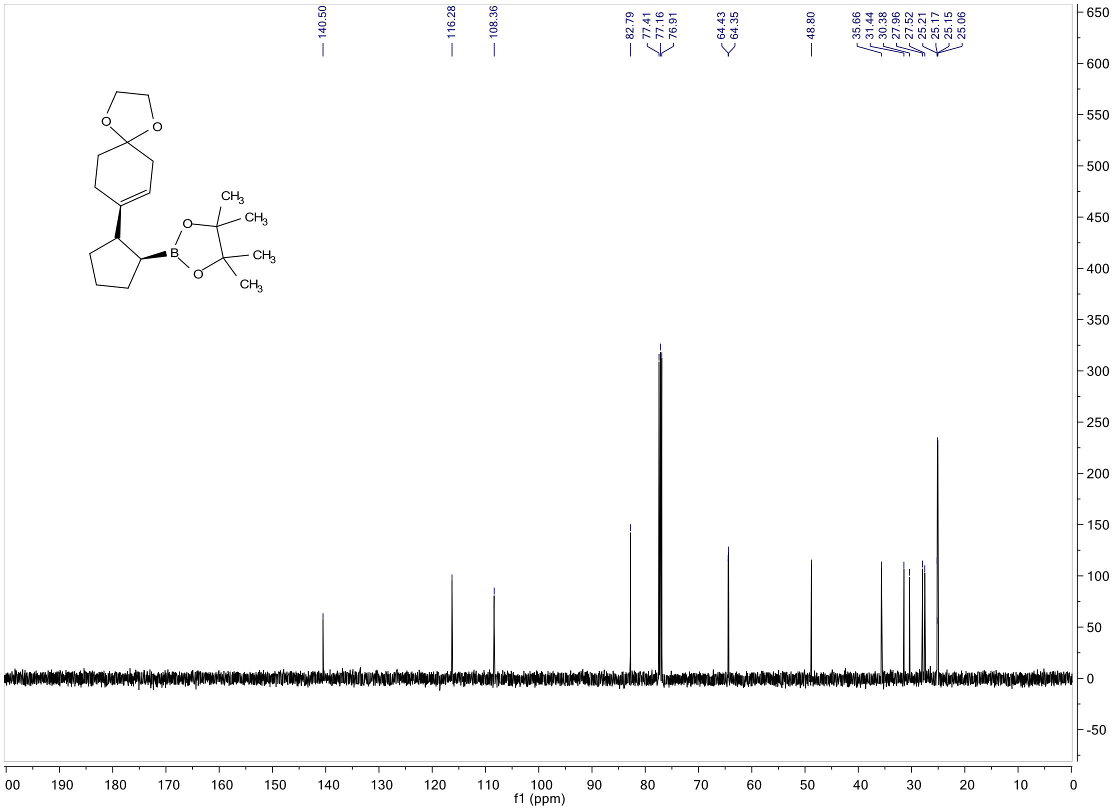

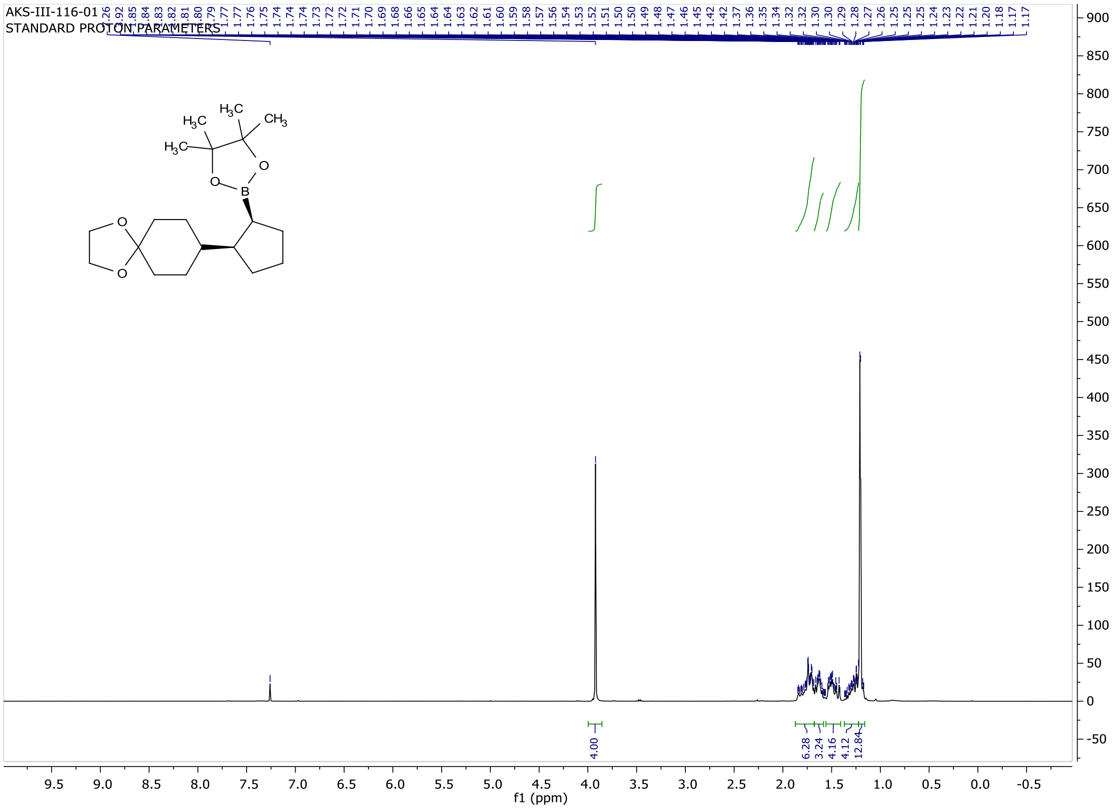
AKS-III-116-01
STANDARD PROTON PARAMETERS
7.26
3.92
1.85 1.84 1.83 1.82 1.81 1.80 1.79 1.77 1.77 1.76 1.75 1.74 1.74 1.74 1.73 1.73 1.72 1.72 1.71 1.70 1.69 1.68 1.66 1.65 1.64 1.63 1.62 1.61 1.60 1.59 1.58 1.57 1.56 1.54 1.53 1.52 1.51 1.50 1.50 1.49 1.48 1.47 1.46 1.45 1.42 1.42 1.37 1.36 1.35 1.34 1.32 1.32 1.30 1.30 1.29 1.28 1.28 1.27 1.26 1.25 1.25 1.24 1.23 1.22 1.21 1.20 1.18 1.17 1.17
4.00
6.28
3.24
4.16
4.12
12.84
f1 (ppm)

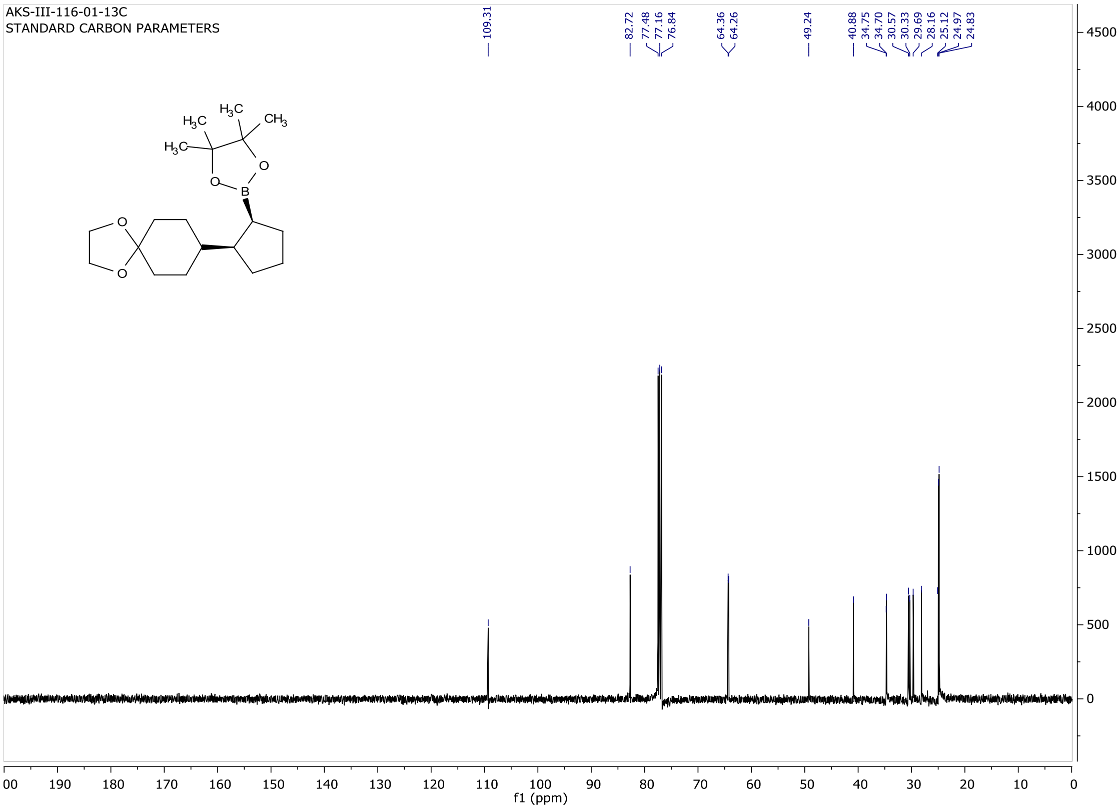
AKS-III-116-01-13C
STANDARD CARBON PARAMETERS
109.31
82.72
77.48
77.16
76.84
64.36
64.26
49.24
40.88
34.75
34.70
30.57
30.33
29.69
28.16
25.12
24.97
24.83
f1 (ppm)

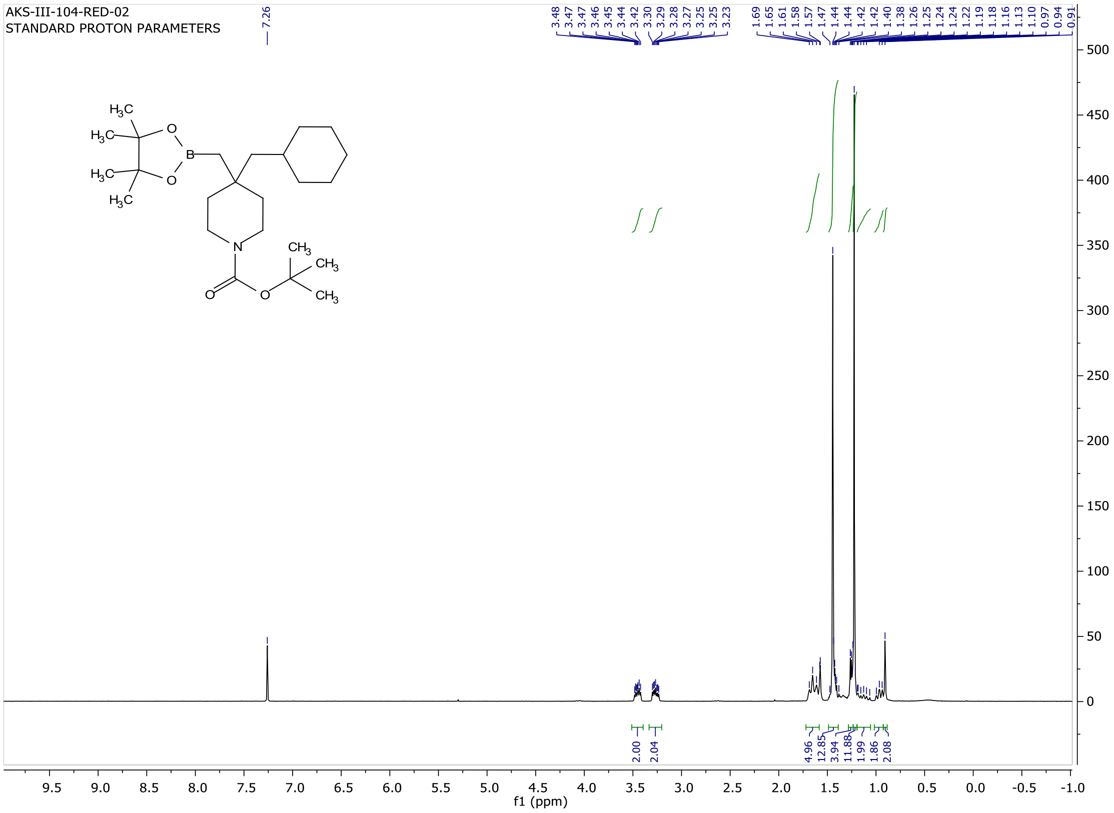
AKS-III-104-RED-02
STANDARD PROTON PARAMETERS
f1 (ppm)

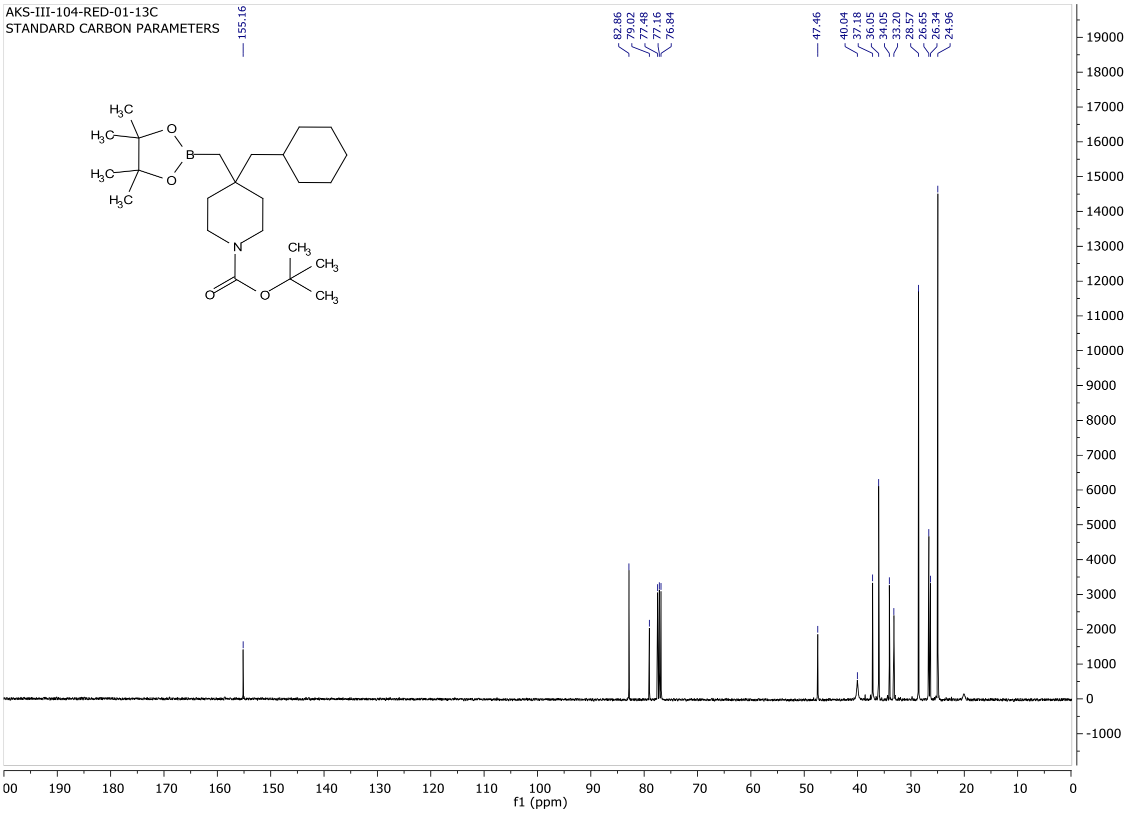
AKS-III-104-RED-01-13C
STANDARD CARBON PARAMETERS
f1 (ppm)

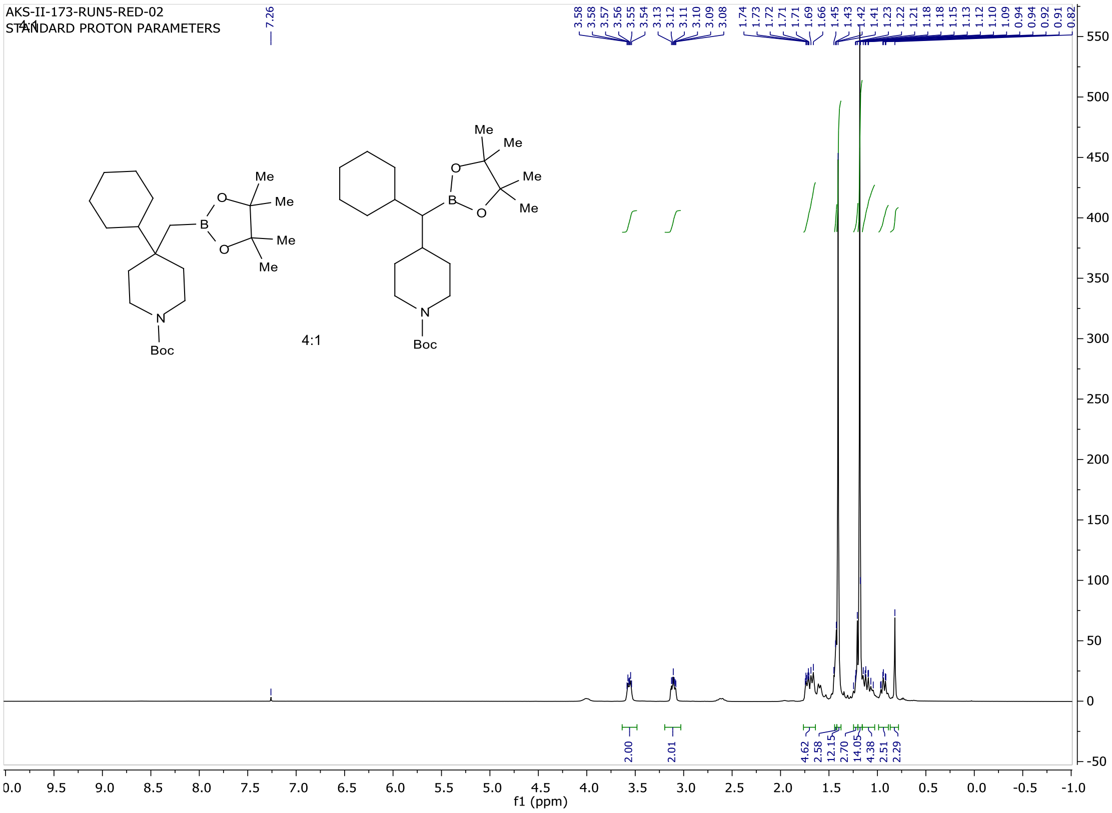
AKS-II-173-RUN5-RED-02
4:1
STANDARD PROTON PARAMETERS
4:1
Boc
Boc
f1 (ppm)

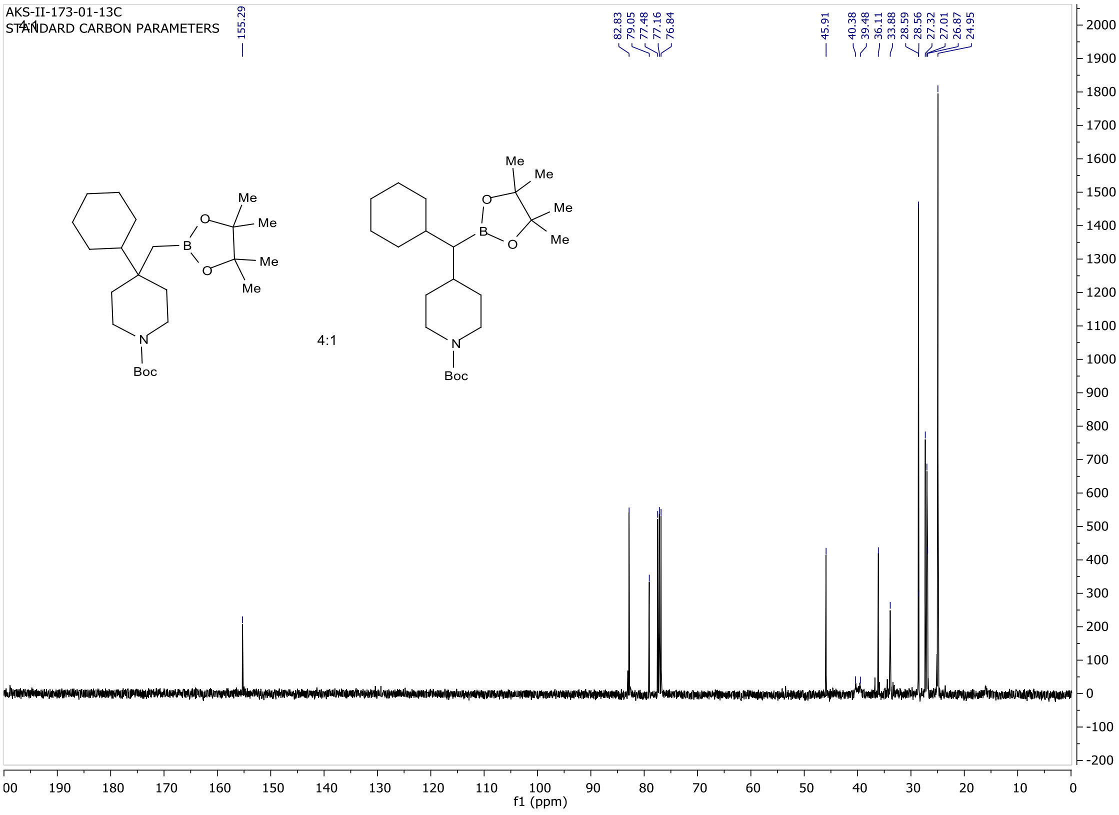
AKS-II-173-01-13C
4:1
STANDARD CARBON PARAMETERS
4:1
Boc
Boc
f1 (ppm)

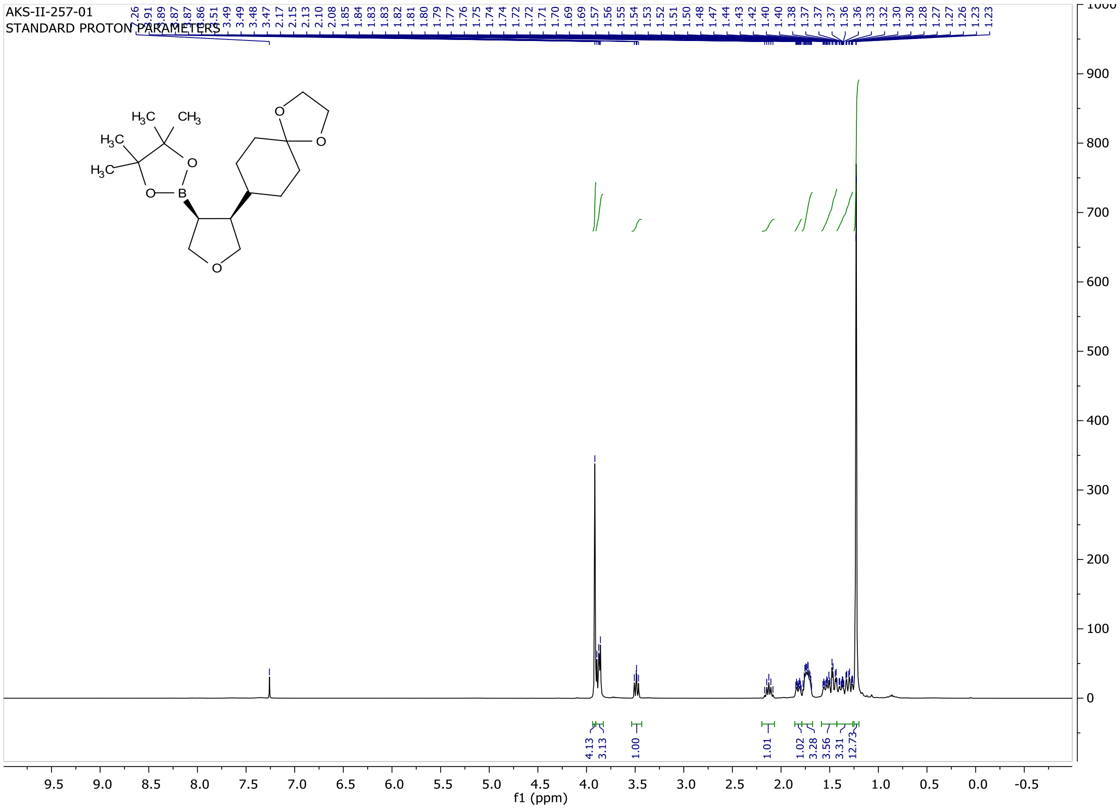
AKS-II-257-01
STANDARD PROTON PARAMETERS
f1 (ppm)

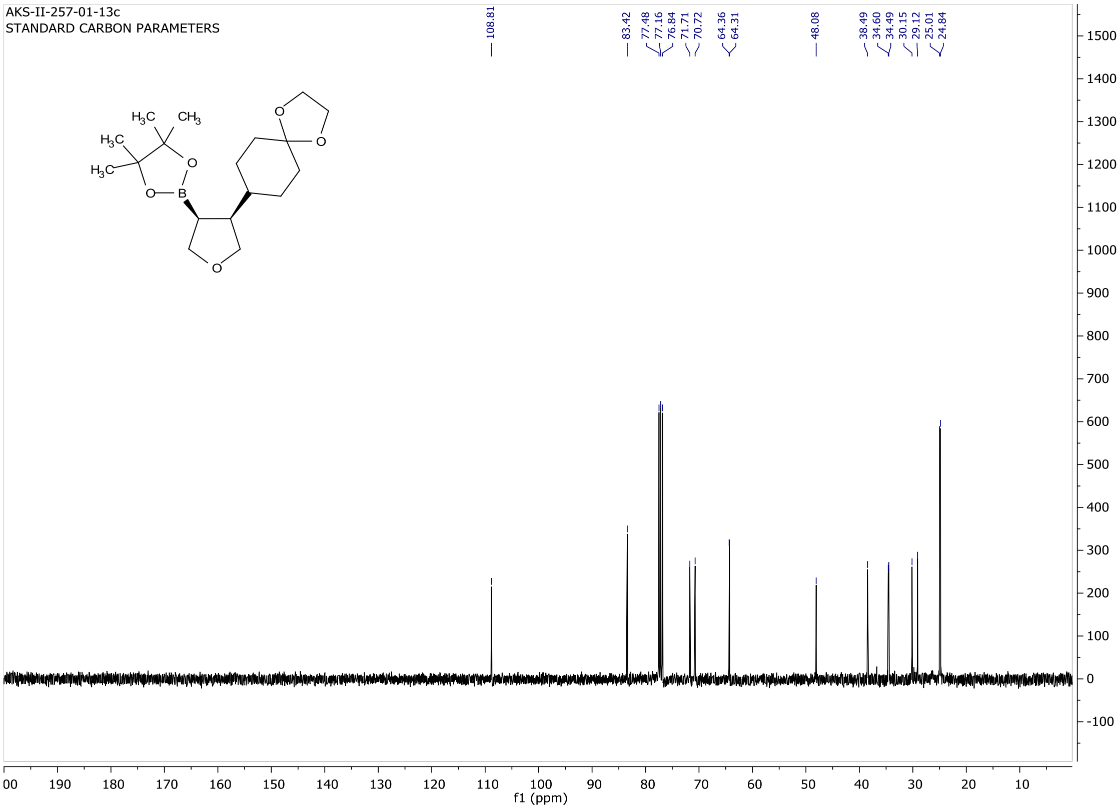
AKS-II-257-01-13c
STANDARD CARBON PARAMETERS
108.81
83.42
77.48
77.16
76.84
71.71
70.72
64.36
64.31
48.08
38.49
34.60
34.49
30.15
29.12
25.01
24.84
f1 (ppm)

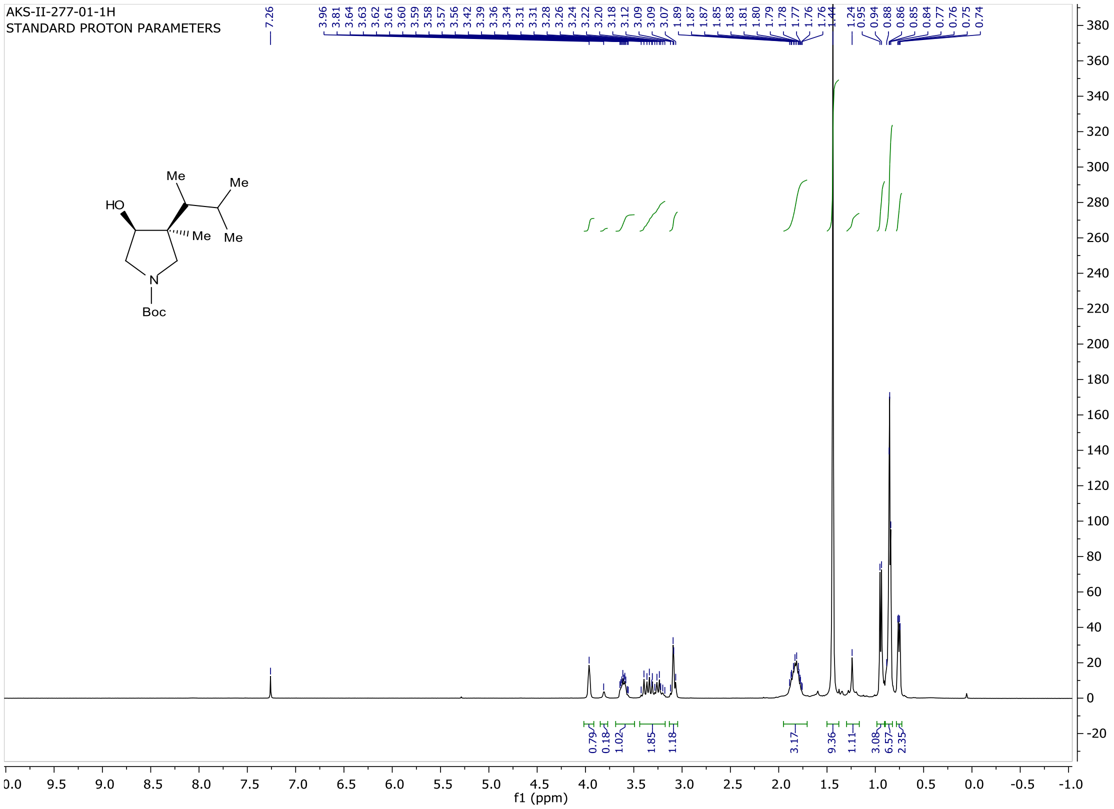
AKS-II-277-01-1H
STANDARD PROTON PARAMETERS
f1 (ppm)

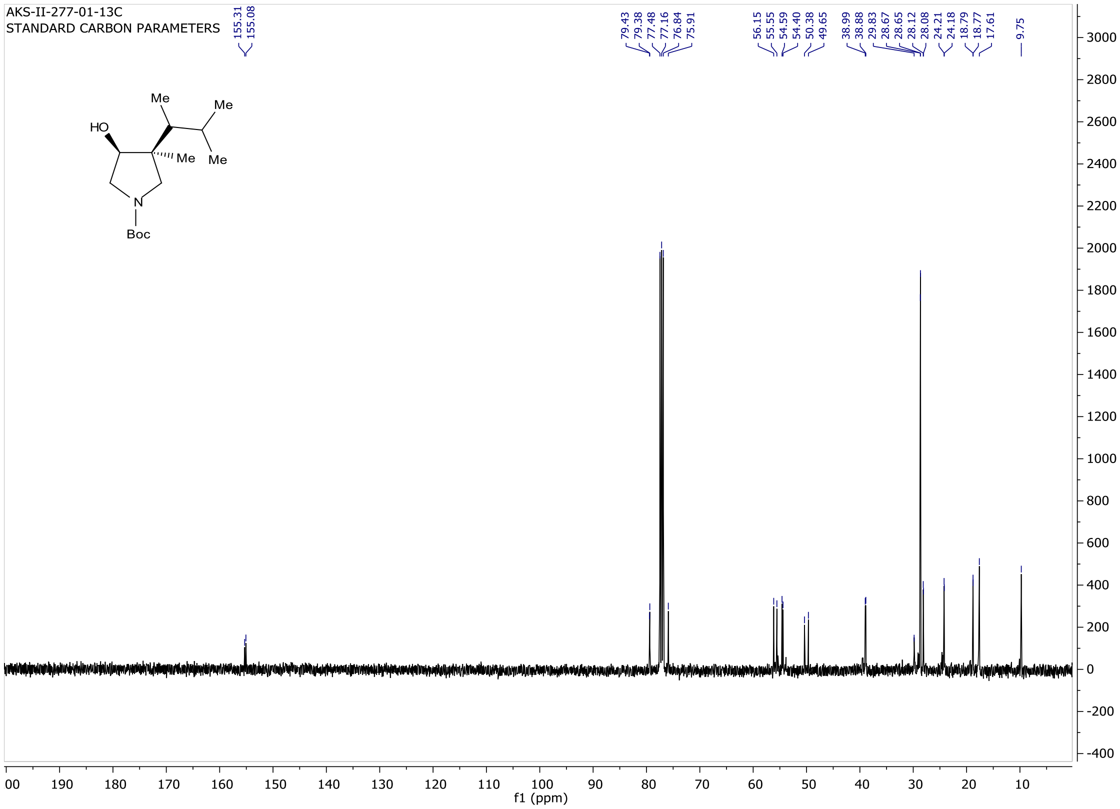
AKS-II-277-01-13C
STANDARD CARBON PARAMETERS
f1 (ppm)

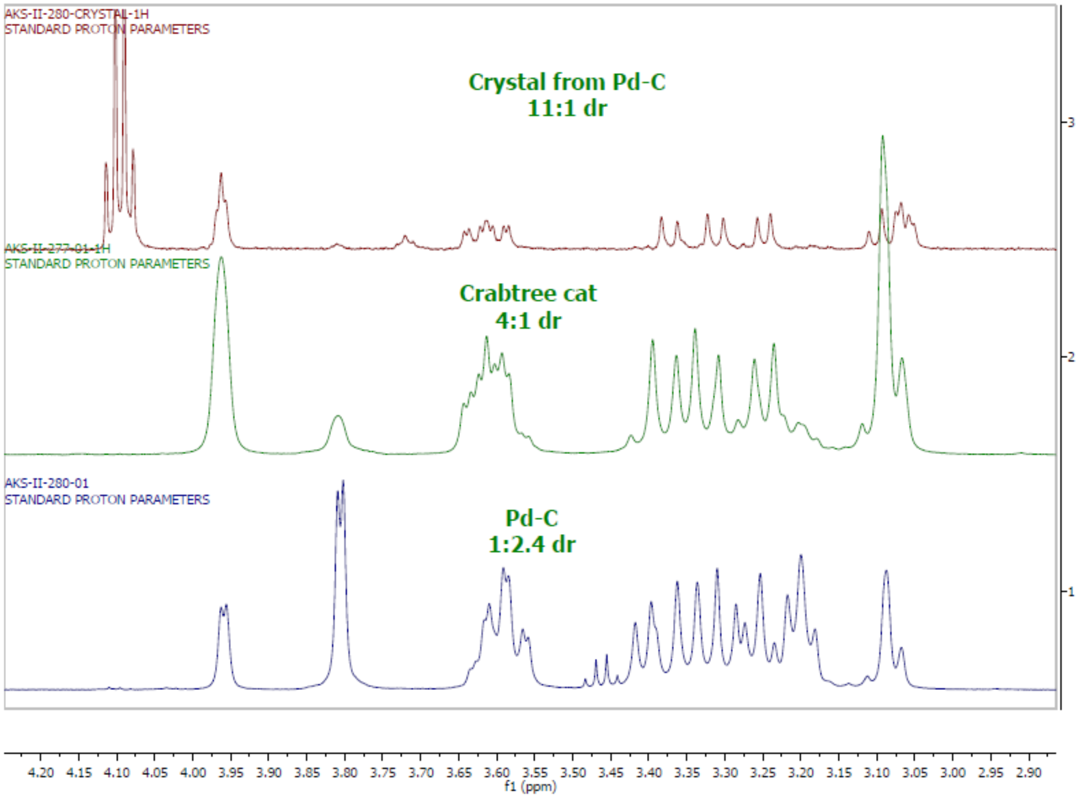
AKS-II-280-CRYSTAL-1H
STANDARD PROTON PARAMETERS
Crystal from Pd-C
11:1 dr
AKS-II-277-01-1H
STANDARD PROTON PARAMETERS
Crabtree cat
4:1 dr
AKS-II-280-01
STANDARD PROTON PARAMETERS
Pd-C
1:2.4 dr
4.20 4.15 4.10 4.05 4.00 3.95 3.90 3.85 3.80 3.75 3.70 3.65 3.60 3.55 3.50 3.45 3.40 3.35 3.30 3.25 3.20 3.15 3.10 3.05 3.00 2.95 2.90
f1 (ppm)

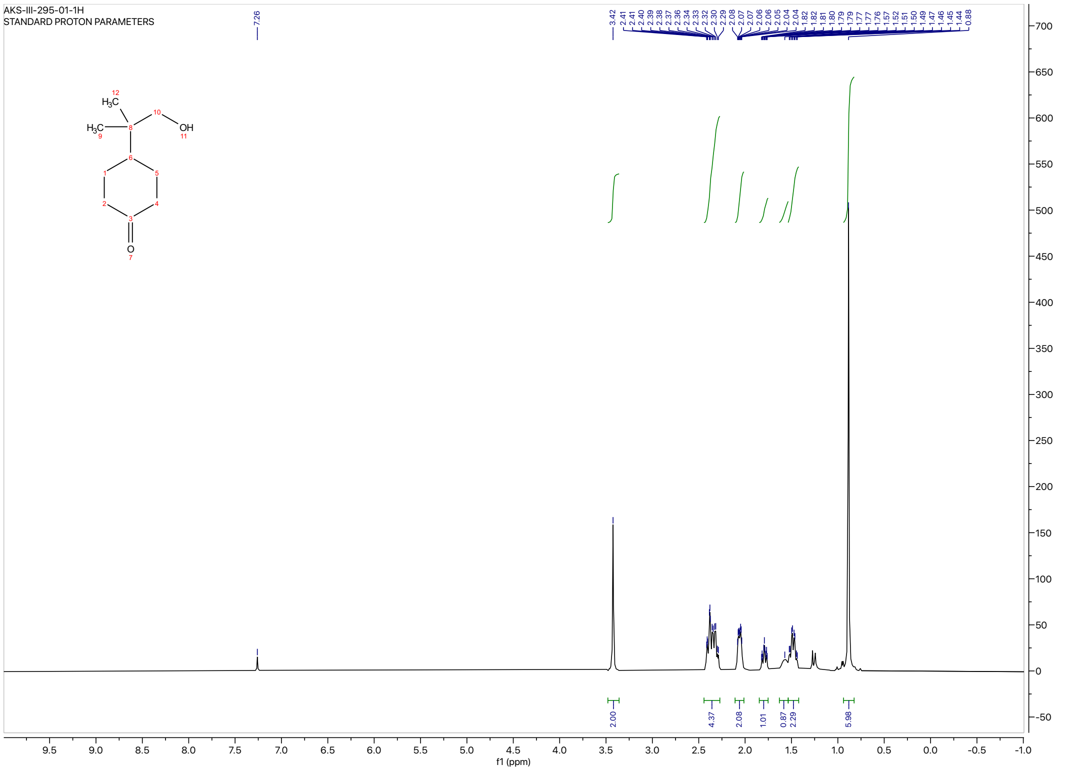
AKS-III-295-01-1H
STANDARD PROTON PARAMETERS
f1 (ppm)

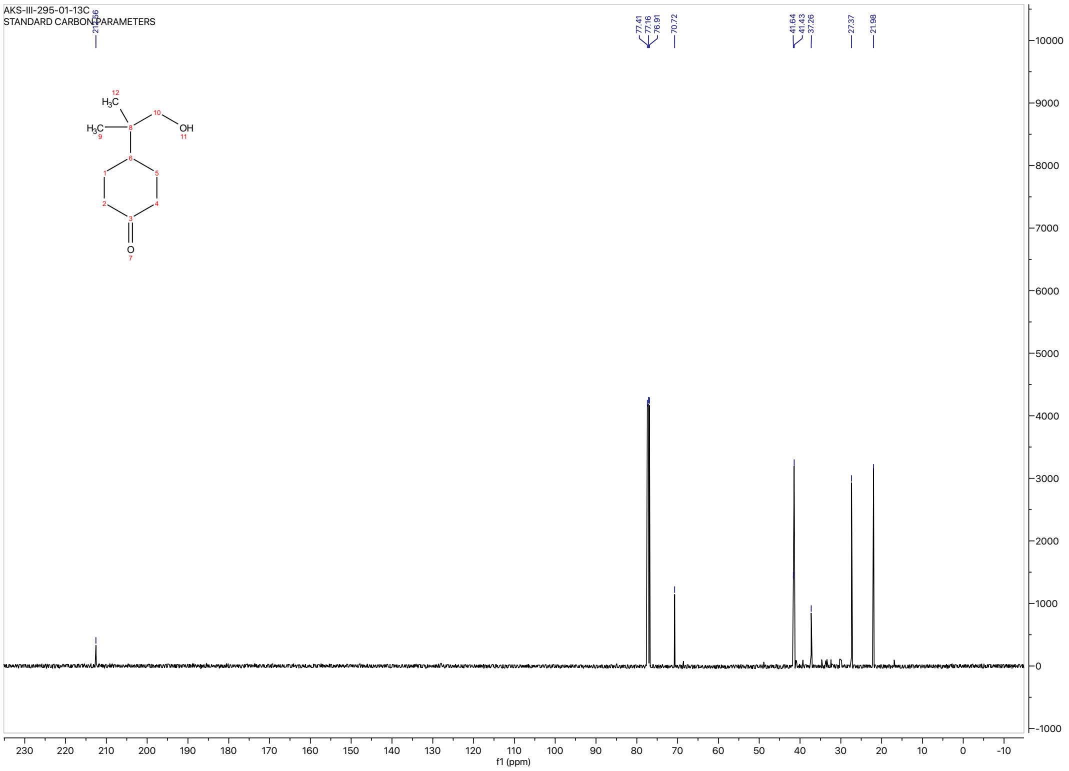
AKS-III-295-01-13C
STANDARD CARBON PARAMETERS
f1 (ppm)

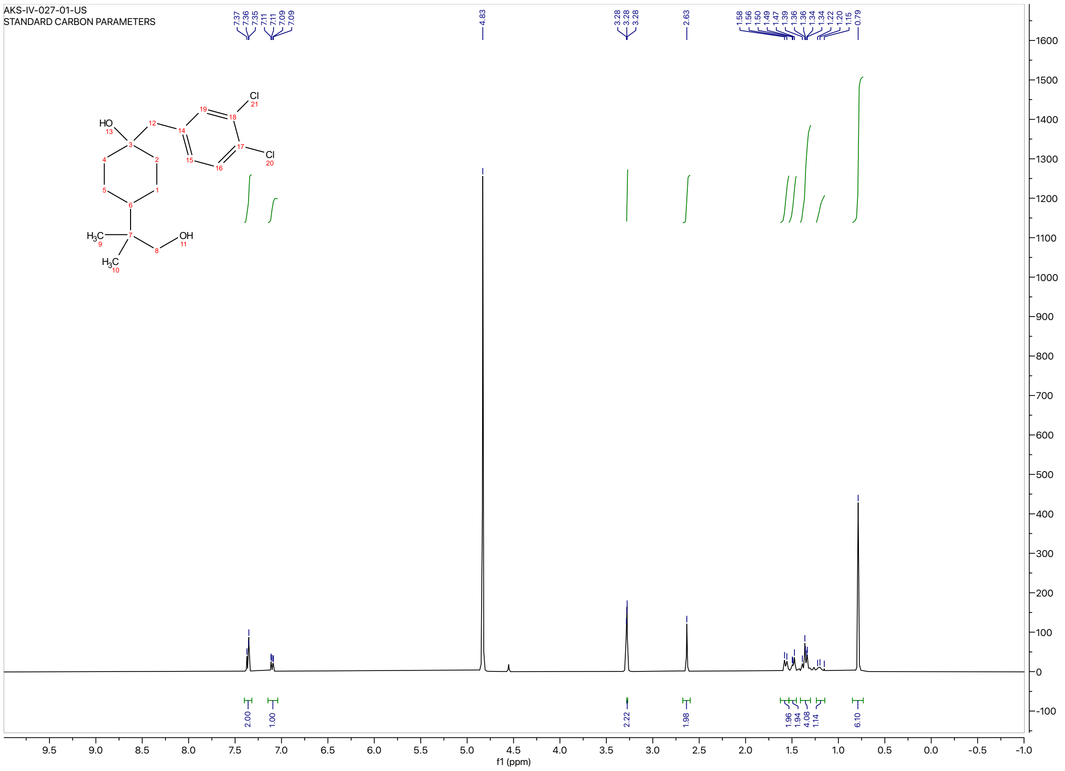
AKS-IV-027-01-US
STANDARD CARBON PARAMETERS
f1 (ppm)

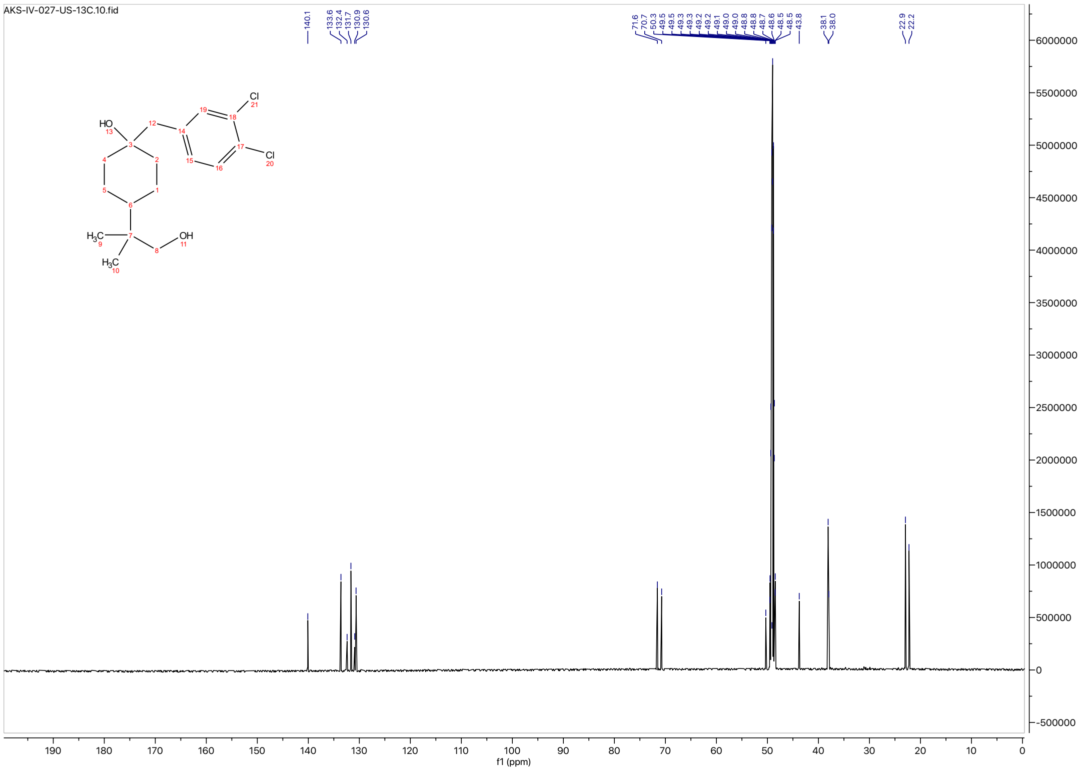
AKS-IV-027-US-13C.10.fid
f1 (ppm)

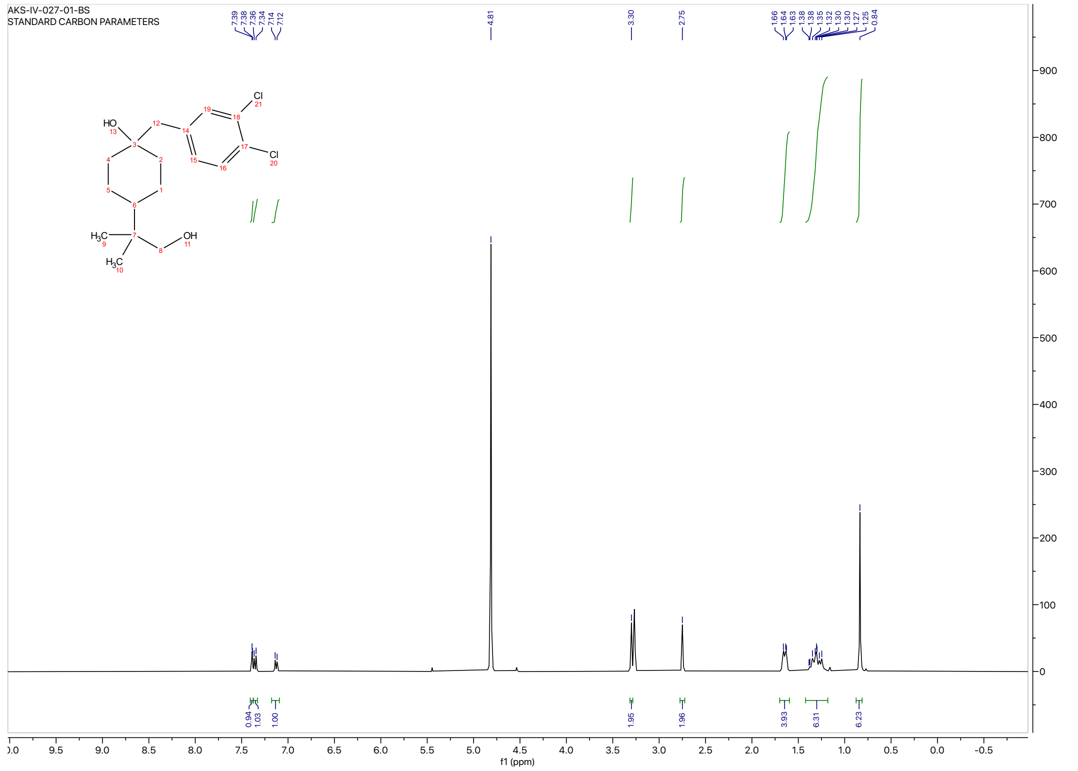

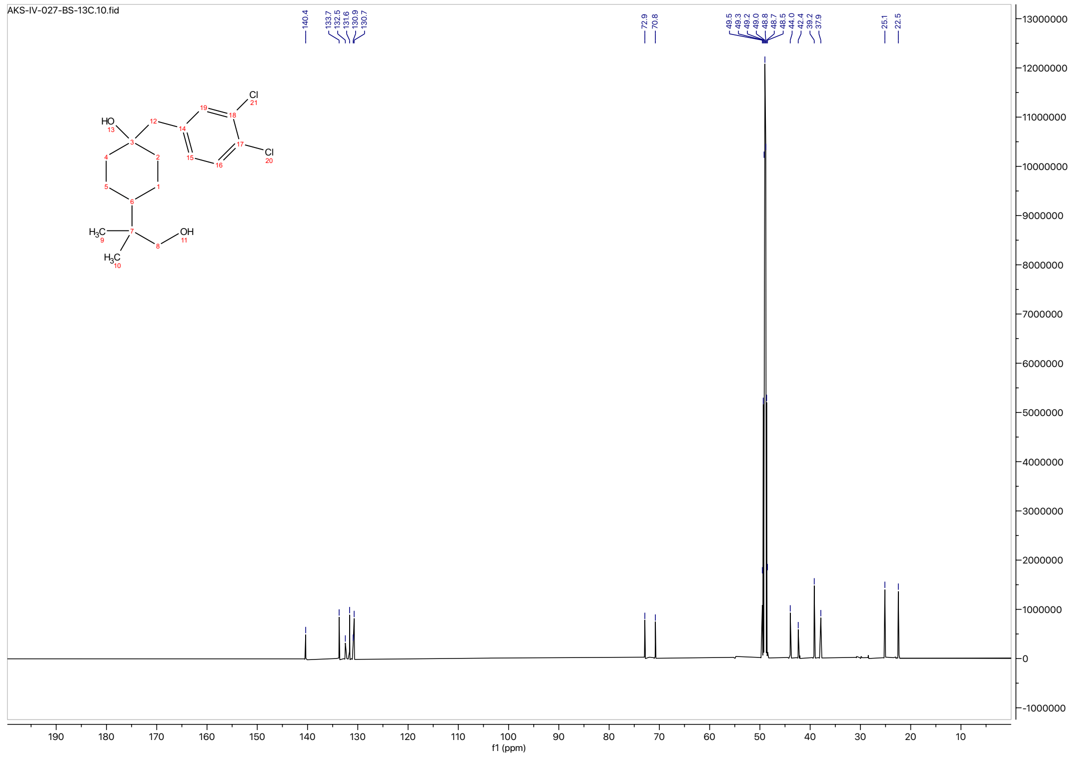

12. References:

1. J. Maibaum, S. Stutz, R. Göschke, P. Rigollier, Y. Yamaguchi, F. Cumin, J. Rahuel, H.-P. Baum, N.-C. Cohen, C. R. Schnell, W. Fuhrer, M. G. Gruetter, W. Schilling and J. M. Wood, *J. Med. Chem.*, 2007, **50**, 4832-4844.
2. S. Liu and J. Zhou, *Chem. Commun.*, 2013, **49**, 11758-11760.
3. S. R. Sardini, A. L. Lambright, G. L. Trammel, H. M. Omer, P. Liu and M. K. Brown, *J. Am. Chem. Soc.*, 2019, **141**, 9391-9400.
4. K. D. Rice, N. Aay, N. K. Anand, C. M. Blazey, O. J. Bowles, J. Bussenius, S. Costanzo, J. K. Curtis, S. C. Defina, L. Dubenko, S. Engst, A. A. Joshi, A. R. Kennedy, A. I. Kim, E. S. Koltun, J. C. Lougheed, J.-C. L. Manalo, J.-F. Martini, J. M. Nuss, C. J. Peto, T. H. Tsang, P. Yu and S. Johnston, *ACS Med. Chem. Lett.*, 2012, **3**, 416-421.
5. D. M. Hodgson, T. J. Miles and J. Witherington, *Tetrahedron*, 2003, **59**, 9729-9742.
6. C. Dallanoce, F. Frigerio, G. Grazioso, C. Matera, G. L. Visconti, M. De Amici, L. Pucci, F. Pistillo, S. Fucile, C. Gotti, F. Clementi and C. De Micheli, *Eur. J. Med. Chem.*, 2011, **46**, 5790-5799.
7. *US patent, US2016/185785. Pat.*
8. R. Beniazza, V. Liautard, C. Poittevin, B. Ovadia, S. Mohammed, F. Robert and Y. Landais, *Chem. – Eur. J.*, 2017, **23**, 2439-2447.
9. J. L. Hofstra, A. H. Cherney, C. M. Ordner and S. E. Reisman, *J. Am. Chem. Soc.*, 2018, **140**, 139-142.
10. S. Wiesler, M. A. Bau, T. Niepel, S. L. Younas, H.-T. Luu and J. Streuff, *Eur. J. Org. Chem.*, 2019, **2019**, 6246-6260.
11. F. Zhan and G. Liang, *Angew. Chem. Int. Ed.*, 2013, **52**, 1266-1269.
12. J. Fei, Z. Wang, Z. Cai, H. Sun and X. Cheng, *Adv. Synth. Catal.*, 2015, **357**, 4063-4068.
13. S. A. Green, T. R. Huffman, R. O. McCourt, V. van der Puyl and R. A. Shenvi, *J. Am. Chem. Soc.*, 2019, **141**, 7709-7714.
14. J. Duan, Y.-F. Du, X. Pang and X.-Z. Shu, *Chem. Sci.*, 2019, **10**, 8706-8712.
15. T. S.-B. Lou, S. W. Bagley and M. C. Willis, *Angew. Chem. Int. Ed.*, 2019, **58**, 18859-18863.
16. P. J. Stang and W. Treptow, *Synthesis*, 1980, **1980**, 283-284.
17. C. Obradors, R. M. Martinez and R. A. Shenvi, *J. Am. Chem. Soc.*, 2016, **138**, 4962-4971.
18. N. B. Zuckerman, A. S. Myers, T. K. Quan, W. M. Bray, R. S. Lokey, G. A. Hartzog and J. P. Konopelski, *Chem. Med. Chem.*, 2012, **7**, 761-765.
